# Synthesis, Biological Evaluation and Mechanism Studies of Deoxytylophorinine and Its Derivatives as Potential Anticancer Agents

**DOI:** 10.1371/journal.pone.0030342

**Published:** 2012-01-19

**Authors:** Haining Lv, Jinhong Ren, Shuanggang Ma, Song Xu, Jing Qu, Zhenjia Liu, Qing Zhou, Xiaoguang Chen, Shishan Yu

**Affiliations:** State Key Laboratory of Bioactive Substance and Function of Natural Medicines, Institute of Materia Medica, Chinese Academy of Medical Sciences and Peking Union Medical College, Beijing, China.; University of Helsinki, Finland

## Abstract

Previous studies indicated that (+)-13a-(*S*)-Deoxytylophorinine (**1**) showed profound anti-cancer activities both *in vitro* and *in vivo* and could penetrate the blood brain barrier to distribute well in brain tissues. CNS toxicity, one of the main factors to hinder the development of phenanthroindolizidines, was not obviously found in **1**. Based on its fascinating activities, thirty-four derivatives were designed, synthesized; their cytotoxic activities *in vitro* were tested to discover more excellent anticancer agents. Considering the distinctive mechanism of **1** and interesting SAR of deoxytylophorinine and its derivatives, the specific impacts of these compounds on cellular progress as cell signaling transduction pathways and cell cycle were proceeded with seven representative compounds. **1** as well as three most potent compounds, **9**, **32**, **33,** and three less active compounds, **12**, **16**, **35,** were selected to proform this study to have a relatively deep view of cancer cell growth-inhibitory characteristics. It was found that the expressions of phospho-Akt, Akt, phospho-ERK, and ERK in A549 cells were greater down-regulated by the potent compounds than by the less active compounds in the Western blot analysis. To the best of our knowledge, this is the first report describing phenanthroindolizidines alkaloids display influence on the crucial cell signaling proteins, ERK. Moreover, the expressions of cyclin A, cyclin D1 and CDK2 proteins depressed more dramatically when the cells were treated with **1**, **9**, **32**, and **33**. Then, these four excellent compounds were subjected to flow cytometric analysis, and an increase in S-phase was observed in A549 cells. Since the molecular level assay results of Western blot for phospho-Akt, Akt, phospho-ERK, ERK, and cyclins were relevant to the potency of compounds in cellular level, we speculated that this series of compounds exhibit anticancer activities through blocking PI3K and MAPK signaling transduction pathways and interfering with the cell cycle progression.

## Introduction

Phenanthroindolizidine alkaloids are pentacyclic natural products isolated mainly from plants belonging to *Cynanchum*, *Pergularia*, *Tylophora* as well as some genera of the *Asclepiadaceae* [1]–[3]. For many years, these natural products and synthetic derivatives attract widespread attention for their extensively therapeutic activities, such as anticancer activity [4]–[8], anti-inflammatory activity [9]–[11], antibacterial activity [12]–[14], and so on. However, the specific biomolecular targets of these compounds on cell growth have not been clearly identified until now. Early studies illustrated that phenanthroindolizidine alkaloids could inhibit RNA, DNA synthesis, and inhibited protein synthesis at the elongation stage of the translation procedure by locating on 40S ribosomal component [15]–[20]. Recently, some possible targets were reported, including metabolic enzymes [21]–[23] and some elements engaged in gene transcription [24], [25]. Moreover, recent research demonstrated that these compounds with similar structures may act on different targets [26]. Although the biological activities of these compounds are affirmative, there are some side effects limiting their application as anticancer drugs, especially CNS toxicity arose in natural tylocrebrine obviously for disorientation and ataxia [27]. And as far as we know, there is not a phenanthroindolizidine alkaloid applied in clinical application up to now. Therefore, it is very pressing to discover novel phenanthroindolizidine alkaloids with profound anticancer activity and reduced CNS toxicity as drug candidates.

(+)-13a-(*S*)-Deoxytylophorinine (**1**), originally isolated from the roots of *Tylophora atrofolliculata* and *Tylophora ovata* in our laboratory (Patent Publication Number: CN101058578A; PCT/CN2010/075083), was found to have profound anti-cancer activities, both *in vitro* and *in vivo* [28]. Liu *et al* [29] discovered that this compound could penetrate the blood brain barrier and distribute in brain tissues without obvious CNS toxicity. Further study confirmed that **1** could interact with DNA and RNA dose-dependently and preferred to intercalate into AT-repeated base pair in double-helical DNA sequences.

Based on the fascinating activities of **1**, thirty-four derivatives were designed and synthesized in our present research. The potential cytotoxic activities of these synthetic compounds against series of human cancer cells in vitro were assessed and the preliminary structure-activity relationships (SAR) were summarized.

**1** could interact with DNA and RNA and concomitantly block the process of transcription to produce the anticancer effects in Liu's research [28], [29]. And high concentrations of **1** could induce cell apoptosis (Figure S1). Previous study indicated that phosphatidylinositol 3-kinase (PI3K) and mitogen-activated protein kinase (MAPK) signaling pathways play a fundamental role in the apoptosis induced by DNA-damaging drugs [30]. Furthermore, only a little information is available regarding the regulation of the PI3K and MAPK signaling transduction pathways in the context of phenanthroindolizidine-induced apoptosis [31]–[34]. Thus, we are inspired to further study the specific impacts of deoxytylophorinine and its derivatives on cell progression as cell signaling transduction pathways and cell cycle. Our study represent a significant step forward in understanding of the cell signaling transduction pathways and cell cycle associated with the apoptosis elicited as the result of exposure to DNA-damaging anticancer agents of deoxytylophorinine and its derivatives.

## Results and Discussion

### Design and synthesis of deoxytylophorinine derivatives

To investigate the influence of the steric propterty at C-13a on cytotoxic activity, enantiomer of **1** ((−)-13a-(*R*)-deoxytylophorinine (**2**)), which was previously isolated from the roots of *Pergularia pallida* plants [35], was synthesized. The amide intermediates were also screened against cancer cell lines to determine the impact of electric property at N-10. With respect to the substituted pattern on phenanthrene ring, derivatives with methoxyl groups in different locations, different number of substitutes, or other substitute such as F in phenanthrene ring were designed. Methoxyl group, amino group, or substituted amino groups were connected to C-14 to reveal the impacts, including the volume of the substitutes at C-14, on cytotoxic activities.

**Figure 1:**
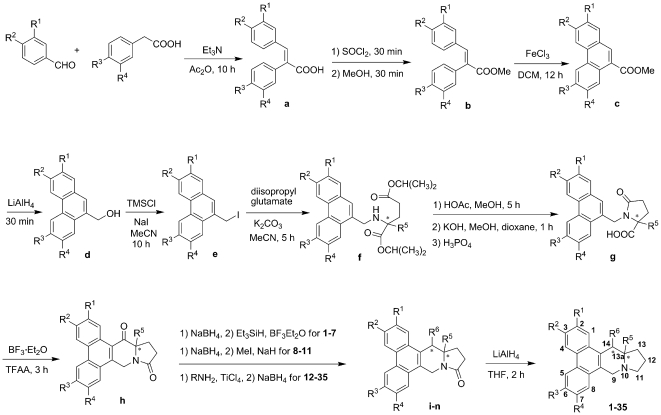
Synthesis of deoxytylophorinine and its derivatives.

Using l-glutamate as chiral building block, **1** was prepared with higher *ee* value than previous reports [36], [37] based on Rapoport's [38] and Ikeda's [39] routes accompanied with some improvement in our synthetic research. As depicted in Figure 1, after condensation, esterification, oxidative cyclization, and reduction [40], [41], the key intermediate **d** was iodized and then condensed in situ with l-glutamate to produce **f**. Subsequently, **g** was synthesized through cyclization and hydrolysis. From **d** to **g**, product in each step can be used directly in the next reaction without purification to facilitate the experiment operation in our research. After Friedel-Crafts acylation and reduction with NaBH_4_, **i** was treated with Et_3_SiH and BF_3_·Et_2_O to give **j**, which was a new strategy to avoiding racemization at C-13a. Finally, the target compound **1** was obtained by reduction with LiAlH_4_ in refluxing THF, with 99% *ee* value and 7% total yield. Thirty-four derivatives were subsequently designed and prepared with this synthetic strategy.

For the synthesis of **2**, same materials and procedures were applied as synthesis of **1** except for the chiral building block of d-glutamate. And five derivatives (**3** [38], **4** [42], **5**, **6** [43], and **7** [43]) with different substitutes at phenanthrene ring of **1** were synthesized with several different substituted benzaldehydes and phenylacetic acids.

14-methoxyl derivatives can be synthesized from the intermediate of **i**. After methylation, the resulting diastereoisomer mixture could be isolated by silica gel column chromatography to afford **k** and **l**. The absolute configuration of C-14 could be verified by ^1^H-NMR. For the intermediate of **k**, H-14 appears as doublet (*J* = 7.0 Hz) at 5.15 ppm, since the dihedral angel between H-13a and H-14 was close to 180° [38]. Thus we confirmed the configuration of C-14 was *R* in **k**. While broad singlet at 5.19 ppm was observed for H-14 in the intermediate of **l**, inferring *S* configuration in this position. This conclusion was further confirmed by NOE measurement of **k** and **l** (Figure S2). The target derivatives **8** (13a*S*, 14*R*) and **9** [44] (13a*S*, 14*S*) were obtained by reduction of **k** and **l** respectively. Their enantiomers **10** (13a*R*, 14*S*) and **11** (13a*R*, 14*R*) could be prepared from the enantiomer of **i** by the same procedures.

Reductive amination was applied to introduce substituted-amino groups at C-14. **h** was reacted with substituted amines to produce the corresponding imines [46], which were subsequently treated with NaBH_4_ to afford diastereoisomer mixtures of **m** and **n**. After isolated from chromatography, **m** and **n** were reduced to corresponding derivatives by LiAlH_4_. Twenty four new derivatives with optically pure properties at C-13a and C-14 were synthesized through this synthetic strategy.

### Biology

Since **1** exerted profound cytoxoic activities *in vitro* [28], its derivatives and five synthetic intermediates (**f**, **g**, **h**, **i**, and **j**) (Table 1) were screened against seven human cancer cell lines in this research to explore their cytotoxic spectra and to discover more potent compounds. Preliminary mechanistic studies, including the influence on cell signaling transduction pathways and cell cycle, were thus performed in order to reveal a more detailed picture on the possible targets.

**Table 1:** Structures of deoxytylophorinine, its intermediate and derivatives.

Compd^a^	R^1^	R^2^	R^3^	R^4^	R^5^	R^6^
**f**	H	OMe	OMe	OMe	H (*S*)	-
**g**	H	OMe	OMe	OMe	H (*S*)	-
**h**	H	OMe	OMe	OMe	H (*S*)	-
**i**	H	OMe	OMe	OMe	H (*S*)	OH
**j**	H	OMe	OMe	OMe	H (*S*)	H
**k**	H	OMe	OMe	OMe	H (*S*)	OMe (*R*)
**l**	H	OMe	OMe	OMe	H (*S*)	OMe (*S*)
**m**	H	OMe	OMe	OMe	H (*S*)	BnNH (*R*)
**n**	H	OMe	OMe	OMe	H (*S*)	BnNH (*S*)
**1**	H	OMe	OMe	OMe	H (*S*)	H
**2**	H	OMe	OMe	OMe	H (*R*)	H
**3**	OMe	OMe	OMe	OMe	H (*S*)	H
**4**	OMe	OMe	OMe	H	H (*S*)	H
**5**	OMe	H	OMe	OMe	H (*S*)	H
**6**	H	F	OMe	OMe	H (*S*)	H
**7**	H	H	OMe	OMe	H (*S*)	H
**8** b	H	OMe	OMe	OMe	H (*S*)	OMe (*R*)
**9**	H	OMe	OMe	OMe	H (*S*)	OMe (*S*)
**10**	H	OMe	OMe	OMe	H (*R*)	OMe (*S*)
**11**	H	OMe	OMe	OMe	H (*R*)	OMe (*R*)
**12** b	H	OMe	OMe	OMe	H (*S*)	BnNH (*R*)
**13** b	H	OMe	OMe	OMe	H (*S*)	BnNH (*S*)
**14**	H	OMe	OMe	OMe	H (*R*)	BnNH (*S*)
**15**	H	OMe	OMe	OMe	H (*R*)	BnNH (*R*)
**16** b	H	OMe	OMe	OMe	H (*S*)	n-PrNH (*R*)
**17** b	H	OMe	OMe	OMe	H (*S*)	n-PrNH (*S*)
**18**	H	OMe	OMe	OMe	H (*R*)	n-PrNH (*S*)
**19**	H	OMe	OMe	OMe	H (*R*)	n-PrNH (*R*)
**20** b	H	OMe	OMe	OMe	H (*S*)	i-PrNH (*R*)
**21** b	H	OMe	OMe	OMe	H (*S*)	i-PrNH (*S*)
**22**	H	OMe	OMe	OMe	H (*R*)	i-PrNH (*S*)
**23**	H	OMe	OMe	OMe	H (*R*)	i-PrNH (*R*)
**24** b	H	OMe	OMe	OMe	H (*S*)	c-PentylNH (*R*)
**25** b	H	OMe	OMe	OMe	H (*S*)	c-PentylNH (*S*)
**26**	H	OMe	OMe	OMe	H (*R*)	c-PentylNH (*S*)
**27**	H	OMe	OMe	OMe	H (*R*)	c-PentylNH (*R*)
**28** b	H	OMe	OMe	OMe	H (*S*)	c-HexylNH (*R*)
**29** b	H	OMe	OMe	OMe	H (*S*)	c-HexylNH (*S*)
**30**	H	OMe	OMe	OMe	H (*R*)	c-HexylNH (*S*)
**31**	H	OMe	OMe	OMe	H (*R*)	c-HexylNH (*R*)
**32** b	H	OMe	OMe	OMe	H (*S*)	NH_2_ (*R*)
**33** b	H	OMe	OMe	OMe	H (*S*)	NH_2_ (*S*)
**34**	H	OMe	OMe	OMe	H (*R*)	NH_2_ (*S*)
**35**	H	OMe	OMe	OMe	H (*R*)	NH_2_ (*R*)

a Compounds **1** [36], [37], **2** [35], **3** [38], **4** [42], **6** [43], **7** [43], **9** [44], and **33** [45] are known, while others are new synthesized.

b These compounds have been claimed in a pending patent (PCT/CN2010/070832).

### Evaluation of deoxytylophorinine derivatives against human cancer cell lines *in vitro* and summary of preliminary SAR study

Two optically pure deoxytylophorinines, their derivatives and five synthetic intermediates were screened for cytotoxicity against seven human tumor cell lines *in vitro*: HCT8 (human colon cancer cell line), U251 (human glioblastoma cancer cell line), HepG2 (human hepatocellular cancer cell line), A549 (human lung adenocarcinoma cell line), A2780 (human ovarian cancer cell line), BGC823 (human gastric cancer cell line) and Capan2 (human pancreatic cancer cell line) by MTT assay and preliminary SAR results were also outlined. HCT8, HepG2, A549, and Capan2 were obtained from ATCC (Virginia, USA) and U251, A2780, and BGC823 were obtained from Cell Culture Center, Institute of Basic Medical Sciences, Chinese Academy of Medical Sciences & Peking Union Medical College (Beijing, China).

As summarized in Table 2, the five synthetic intermediates were inactive in all cell lines, with IC_50_ value more than 10 µM. Interestingly, **j**, the amide derivative of **1**, possessed dramatically decreased potency. The ketone group at C-11 in **j** changes the tertiary amine in the indolizidine nucleus to an amide, decreasing the electron density at nitrogen-atom which may be important in the electrostatic interaction with the target. Such a finding is in agreement with the previous reports that C-9 amide structures of various phenanthroindolizidines were much less potent in cancer cell growth assays [8], [47]. Other synthetic intermediates, **h** and **i**, also confirmed this conclusion and displayed significantly reduced activities.

**Table 2:** Cytotoxic activities of deoxytylophorinine and its derivatives *in vitro.*

	IC_50_ a(µM)						
Compd	HCT8	U251	HepG2	A549	A2780	BGC823	Capan2
**f**	>10^**, ##^	>10^**, ##^	>10^**, ##^	>10^**, ##^	>10^**, ##^	>10^**, ##^	>10^**, ##^
**g**	>10^**, ##^	>10^**, ##^	>10^**, ##^	>10^**, ##^	>10^**, ##^	>10^**, ##^	>10^**, ##^
**h**	>10^**, ##^	>10^**, ##^	>10^**, ##^	>10^**, ##^	>10^**, ##^	>10^**, ##^	>10^**, ##^
**i**	>10^**, ##^	>10^**, ##^	>10^**, ##^	>10^**, ##^	>10^**, ##^	>10^**, ##^	>10^**, ##^
**j**	>10^**, ##^	>10^**, ##^	>10^**, ##^	>10^**, ##^	>10^**, ##^	>10^**, ##^	>10^**, ##^
**1**	0.31±0.19	0.34±0.14	0.13±0.10	0.14±0.08	0.27±0.20	1.17±1.17	0.34±0.19
**2**	1.54±0.86	1.34±0.77	1.08±0.70	1.74±1.08	2.82±0.23^**, ##^	8.54±2.53^*, ##^	1.90±1.22
**3**	0.29±0.13	0.89±0.63	0.75±0.57	4.77±2.85*	>10^**, ##^	9.31±1.19^**, ##^	8.92±1.87^**, ##^
**4**	2.25±1.48	1.42±0.97	0.79±0.21^**, #^	1.27±0.74	5.52±4.00	7.79±2.19^**, ##^	0.61±0.30
**5**	0.42±0.08	1.73±1.00	1.19±1.15	2.71±1.52^*,#^	9.27±0.63^**, ##^	7.30±4.67	>10^**, ##^
**6**	0.22±0.17	0.38±0.15	0.26±0.14	0.27±0.08	0.19±0.14	0.42±0.05	0.92±0.75
**7**	0.25±0.11	0.41±0.17	0.22±0.03	0.42±0.18	>10^**, ##^	0.59±0.19	0.69±0.20
**8**	4.29±0.85^**, ##^	3.14±1.51^*, #^	3.25±0.77^**, ##^	4.57±1.26^**, ##^	8.05±1.99^**, ##^	9.49±0.55^**, ##^	6.70±3.02^*, #^
**9**	0.05±0.01^#^	0.07±0.05^*^	0.15±0.16	0.07±0.03	0.08±0.02^#^	0.08±0.03	0.19±0.21
**10**	0.45±0.08	0.70±0.33^#^	0.44±0.17	2.65±1.52^*^	8.82±2.04^**, ##^	6.58±3.79	2.79±0.99^*^
**11**	3.09±0.98^**, #^	4.69±1.96^*, #^	0.98±0.56	2.09±1.15^*, #^	7.59±2.50^**, ##^	6.77±3.39^#^	1.99±1.48
**12**	1.85±1.21	2.36±1.74	2.52±1.13^*, #^	4.98±1.80^**, #^	3.93±0.94^**, ##^	6.88±2.94^*, #^	4.03±1.97*
**13**	5.59±0.32^**, ##^	3.35±0.97^**, ##^	4.00±0.41^**, ##^	4.84±1.18^**, ##^	8.87±1.96^**, ##^	9.85±0.26^**, ##^	6.67±0.54^**, ##^
**14**	3.17±1.62^*^	3.56±1.19^**, ##^	3.09±0.85^**, ##^	4.52±1.94^*, #^	8.31±2.55^**, ##^	7.44±2.55^*, #^	6.30±1.24^**, #^
**15**	7.77±3.86^*, #^	6.38±3.16^*, #^	9.03±1.69^**, ##^	7.61±2.38^**, ##^	>10^**, ##^	>10^**, ##^	9.14±1.50^**, ##^
**16**	5.35±1.60^**, ##^	2.77±1.59^#^	3.02±0.92^**, ##^	3.71±0.72^**, ##^	8.72±2.21^**, ##^	7.29±3.35^*, #^	3.13±1.16^*^
**17**	5.57±1.05^**, ##^	3.17±0.79^**, ##^	3.87±0.63^**, ##^	4.91±0.84^**, ##^	6.81±2.62^*, #^	9.38±1.08^**, ##^	4.07±1.26^**^
**18**	2.93±1.47^*^	2.13±1.02^*, #^	2.40±0.76^**, ##^	2.83±1.39^*, #^	5.51±3.32	5.07±4.27	9.02±1.03^**, ##^
**19**	7.06±1.72^**, ##^	5.14±0.90^**, ##^	4.10±0.79^**, ##^	5.27±1.21^**, ##^	9.20±1.39^**, ##^	9.50±0.86^**, ##^	6.36±3.33^*^
**20**	4.72±0.36^**, ##^	4.02±0.31^**. ##^	4.88±1.64^**, ##^	4.26±1.03^**, ##^	6.66±1.02^**, ##^	7.95±1.79^**, ##^	5.81±2.43^*^
**21**	4.95±0.40^**, ##^	3.24±0.74^**, ##^	4.27±1.17^**, ##^	4.06±1.33^**, ##^	4.77±0.92^**, ##^	8.26±1.77^**, ##^	5.08±1.49^**, #^
**22**	3.01±0.97^**, #^	2.63±0.63^**, ##^	2.45±0.62^**, ##^	3.34±0.73^**, ##^	3.33±0.83^**, ##^	4.55±2.78	3.67±2.85
**23**	7.69±2.01^**, ##^	8.20±1.05^**, ##^	5.09±0.75^**, ##^	7.33±3.42^*, #^	>10^**, ##^	9.31±1.19^**, ##^	6.30±3.25^*^
**24**	4.17±1.30^**, ##^	3.51±1.83^*, #^	2.34±1.39	3.18±1.57^*, #^	6.67±3.06^*, #^	7.60±2.14^*, ##^	4.10±1.30^**^
**25**	5.68±0.97^**, ##^	3.30±0.69^**, ##^	2.85±0.35^**, ##^	3.91±0.40^**, ##^	5.21±1.08^**, ##^	7.77±2.13^**, ##^	6.44±1.26^**, #^
**26**	5.86±2.31^*, #^	5.61±1.56^**, ##^	4.54±1.30^**, ##^	4.64±0.96^**, ##^	9.25±1.29^**, ##^	>10^**, ##^	7.42±1.66^**, #^
**27**	5.74±1.08^**, ##^	4.62±1.34^**, ##^	3.77±1.31^**, ##^	6.32±3.44^*, #^	6.21±2.73^*, #^	8.51±1.30^**, ##^	8.02±3.42^*, #^
**28**	4.81±0.94^**, ##^	4.51±0.59^**, ##^	3.36±0.83^**, ##^	5.96±3.34^*, ##^	8.46±1.81^**, #^	7.46±2.61^*, #^	8.29±1.57^**, ##^
**29**	3.76±1.51^*, #^	3.23±0.79^**, ##^	3.45±1.38^*, #^	6.05±3.52^*, #^	4.61±0.16^**, ##^	7.42±2.66^*, #^	5.77±2.62^*^
**30**	4.58±0.75^**, ##^	3.82±1.28^**, ##^	3.48±1.16^**, ##^	4.92±1.84^*, #^	4.83±0.34^**, ##^	6.02±3.45	8.14±2.07^**, #^
**31**	5.97±1.25^**, ##^	3.76±0.30^**, ##^	3.40±0.27^**, ##^	5.90±3.43^*, #^	7.86±1.95^**, ##^	6.17±0.14^**, ##^	9.50±0.86^**, ##^
**32**	0.02±0.02^#^	0.05±0.01^*^	0.05±0.01	0.07±0.02	4.56±1.23^**, ##^	0.11±0.11	0.42±0.30
**33**	0.03±0.01^#^	0.04±0.01^*^	0.02±0.01	0.40±0.15	0.05±0.04,^#^	0.26±0.13	0.43±0.21
**34**	7.60±0.52^**, ##^	6.27±1.02^**, ##^	6.75±2.81^*, #^	6.94±2.72^*, #^	9.20±1.39^**, ##^	>10^**, ##^	4.70±0.59^**, #^
**35**	3.97±1.03^**, ##^	3.89±0.00^**, ##^	5.78±3.67	4.61±1.15^**, ##^	6.69±3.43^*, #^	9.05±1.28^**, ##^	3.31±0.70^**^
**Doxorubicin**	0.61±0.25	0.13±0.09	0.23±0.25	0.23±0.16	0.63±0.27	0.97±0.92	1.57±1.45

a IC_50_ values are the test compounds concentration (µM) that inhibited the cell growth by 50%. Data represent the mean values ± standard deviation of three dependent experiments performed in triplicate (*, p<0.05 compared with compound **1**; **, p<0.01 compared with compound **1**; ^#^, p<0.05 compared with Doxorubicin; ^##^, p<0.01 compared with Doxorubicin). These IC_50_ values were all measured for 72 h treatment. IC_50_ values for 24 h treatment were in Table S1.

As substitutes in phenanthrene ring to be concerned, a tempting phenomenon can be seen that **3**, **5** with methoxyl group at C-2 in the phenanthrene ring were higher selective than **1** toward various tumor cells. Additionally, **6** bearing a fluorine atom at the C-3 and **7** without any substitute at this position, exhibited comparative activity to the parent structure.

For the 3, 6, 7-trimethoxyl derivatives, the steric properties of C-13a and the volume of substitutes at C-14 are two crucial factors influencing their potency. As summarized in Table 2, **1** with S configuration at C-13a exerted profound cytotoxic activities against all of the cancer cells *in vitro*, and were about 10-fold more potent than its enantiomer **2**. Coincident results were observed for **9** and **10**, **32** and **35**, **33** [45] and **34**, confirming that the steric propterty of C-13a play a significant role in cytotoxic activities. Bearing methoxyl group at the C-14 with S configuration, **9** showed about a 10-fold increased potency compared to the precursor **1**. Positioning of the amino group at the C-14, no matter what configuration is, leads to a remarkable enhancement of cytotoxic abilities against several cancer cell lines, as **32** and **33** with IC_50_ ranging from 15 to 40 nM were much stronger than their parent structure and the positive control of Doxorubicin. However, when the amino group connected with other substituents, neither linear nor circular, the cytotoxic activities decreased dramatically, as can bee seen in **12–31**. **9**, **32**, and **33** exhibited greater cytotoxic activity than **1**, indicating that bearing small group at C-14 could improve the parent-compound efficacy. Therefore, keeping S configuration at C-13a and bearing small group at C-14 were necessary for the enhancement of their potency.

The above initial SAR results may be helpful for the subsequent structure modification to design novel and potent cytotoxic deoxytylophorinine derivatives.

Since **1** was confirmed to interact with DNA and RNA [28], [29], what will happen to the down-stream cellular processes? Based on previous reports that PI3K and MAPK signaling transduction pathways played a fundamental role in the apoptosis induced by DNA-damaging drugs, we investigated the influence of deoxytylophorinine derivatives on these two signaling transduction pathways. Moreover, PI3K and MAPK signaling transduction pathways have emerged as promising molecular targets in the prevention of cancers, by influencing a large variety of cellular processes, such as cell apoptosis, survival, and cell-cycle regulation [48], [49]. Subsequently, the influence of these compounds on cell-cycle regulation was also studied as objects to explore the antitumor effects and mechanisms. Besides **1**, three most potent compounds **9**, **32**, **33** and three less active compounds **12**, **16**, **35** were chosen to process this research.

### Effects of 1, 9, 12, 16, 32, 33 and 35 on the PI3K and MAPK signaling transduction pathway in A549 cells

**Figure 2:**
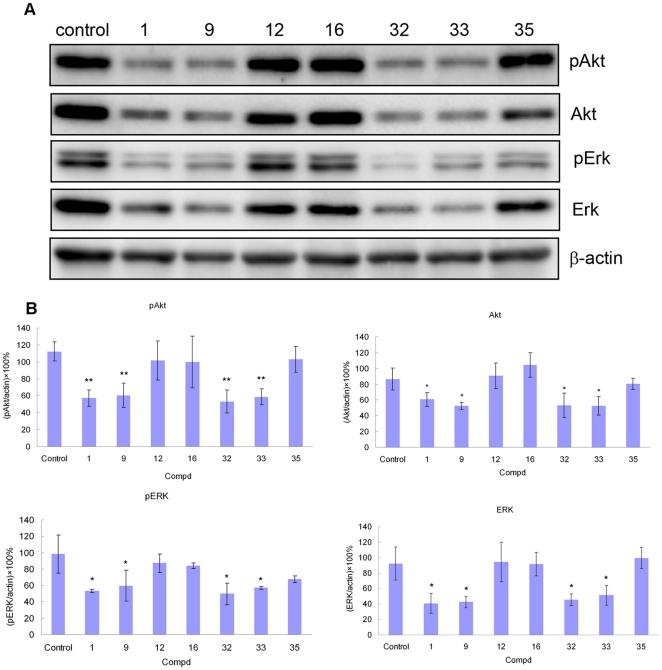
Effects of 1, 9, 12, 16, 32, 33, and 35 on phosphorylated and total proteins of Akt and ERK in A549 cells. A549 cells were untreated or treated with 500 nM of 1, 9, 12, 16, 32, 33, and 35 for 24 h. Following 24 h of recovery, cell lysates were prepared and equal amounts of protein were analyzed by SDS-PAGE. Immunoblots of cellular lysates were analyzed by antibodies of phosphorylated and total proteins of Akt and ERK, with the expression of β-actin as an internal control. A. one of the selected immunoblot analysis results of pAkt, Akt, pERK, ERK and β-actin. B. densitometric analysis results of pAkt, Akt, pERK, and ERK normalized to β-actin expression. Each histogram represents the mean values ± standard deviation of three dependent experiments. (*, p<0.05 compared with control; **, p<0.01 compared with control).

The PI3K signaling pathway is probably the best characterized and most prominent pathway with regard to the transmission of anti-apoptotic signals in cell survival [49], [50]. The MAPK signaling transduction pathway is also known to play crucial roles in cell progressions [48]. The Western blot analysis results indicated that deoxytylophorinine derivatives could influence PI3K signaling pathway, since incubation of A549 cells with 500 nM of potent compounds **1**, **9**, **32**, and **33** could trigger the down-regulation of phospho-Akt and total-Akt compared with that in the control and in less active compounds **12**, **16**, and **35**. As a higher expression of phospho-Akt and Akt is linked to proliferation pathways, this down-regulatory property of tested compounds might contribute to their anticancer action. We also examined whether selected compounds had an inhibition on mitogen-activated protein kinase ERK activity by Western blot assay. As illustrated in Figure 2, the potent compounds **1**, **9**, **32**, and **33** also exerted more notable inhibitory effects on ERK phosphorylation than other groups. Since potent compounds against tested cancer cell lines consistently exhibited intense suppression of Akt and ERK activation than the less active compounds, we can deduce that the potent inhibitory effects of these compounds on the two signaling transduction pathways may be means of their anticancer activities. Thus, we have provided novel insight into the understanding of the underlying molecular mechanisms of these synthesized phenanthroindolizidine alkaloids.

### Effects of 1, 9, 12, 16, 32, 33, and 35 on the cell cyclins in A549 cells

**Figure 3:**
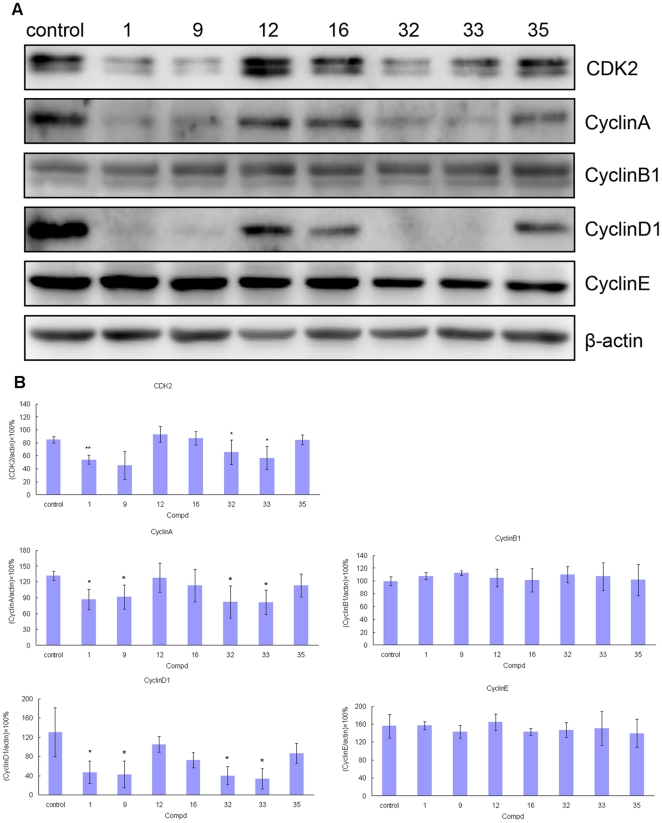
Effects of 1, 9, 12, 16, 32, 33, and 35 on cyclins in A549 cells. A549 cells were untreated or treated with 500 nM of 1, 9, 12, 16, 32, 33, and 35 for 24 h. Following 24 h of recovery, cell lysates were prepared and equal amounts of protein were analyzed by SDS-PAGE. Immunoblots of cellular lysates were analyzed by antibodies of CDK2, cyclin A, cyclin B1, cyclin D1, and cyclin E, with the expression of β-actin as an internal control. A. one of the selected immunoblot analysis results of CDK2, CyclinA, CyclinB1, CyclinD1, CyclinE and β-actin. B. densitometric analysis results of CDK2, CyclinA, CyclinB1, CyclinD1, and CyclinE normalized to β-actin expression. Each histogram represents the mean values ± standard deviation of three dependent experiments. (*, p<0.05 compared with control; **, p<0.01 compared with control).

It is well known that cell proliferation is generally regulated by controlling cell cycle progression through promoting or inhibiting the activities of cyclins/cyclin-dependent kinases (CDKs) complexes or their associated proteins [51]. The seven selected typical compounds (**1**, **9**, **12**, **16**, **32**, **33**, and **35**) were then evaluated for their effects on the regulatory proteins responsible for cell cycle progression, such as cyclin A, cyclin B1, cyclin D1 and cyclin E in A549 cells. **1**, **9**, **32**, and **33** show more remarkable down-regulation of cyclin A, cyclin D1 and CDK2 than the control and the less active compounds **12**, **16**, and **35** (Figure 3), parallel to the results in the cytotoxicity assay, whereas the level of the cyclin B1 and cyclin E did not change significantly. Cyclin D1 overexpression has been observed in a number of human tumors and is associated with poor prognosis and chemoresistance. Therefore, the down-regulatory effect of cyclin D1 also contributes to the antitumor activity of these potent compounds, and this conclusion was in agreement with previous reports by Gao *et al* [26]. These results indicate that these compounds inhibit cell growth through the interruption of some proteins involved in the cell cycle progression.

### Induction of S-phase arrest in A549 cells by 1, 9, 32, and 33

Cell cycle modulators that halt uncontrollable tumor growth are regarded as highly promising therapeutic agents against human cancers. Many natural products exhibited growth inhibitory activities on cancer cells through cell cycle regulation [24], [52]. Based on the results that **1**, **9**, **32**, and **33** were potent compounds at inhibiting cell growth and reducing the expressions of cyclin A, cyclin D1 and CDK2, we further explored the effects of these four compounds on cell cycle distribution by flow cytometric assay.

As revealed by flow cytometric analysis (Table 3), suppression of cell proliferation with 500 nM of those four potent compounds was accompanied by the significant accumulation of cells in the S-phase in A549 cells as compared with the control. For instance, cell populations in the G_0_/G_1_, S, G_2_/M (%) phases were 59.95%, 31.25%, and 8.80% respectively in the control group. However, after exposure to compounds **1**, **9**, **32**, and **33** for 24 h, cell populations in the S-phase were noticeably enhanced by 6%, 14%, 18%, and 11% respectively. Student's *t* test was used to examine the significance of the values. For S-phase in A549 cells, statistical analysis results were: **1** compared with control, P<0.01; **9** compared with control, P<0.05; **32** compared with control, P<0.05; and **33** compared with control, P<0.05. This increase in the proportion of cells in the S-phase was accompanied by a concomitant decreased proportion of cells in the G_0_/G_1_ and G_2_/M phase.

**Table 3:** The effects of 1, 9, 32 and 33 on cell cycle distribution in A549 cells.^a^

	subG_0_/G_1_ (%)	G_0_/G_1_ (%)	S (%)	G_2_/M (%)
**control**	**1.53±0.22**	**59.95±0.21**	**31.25±0.78**	**8.80±0.42**
**1** (500 nM)	5.18±4.41	54.15±0.35^**^	37.40±0.14^**^	8.45±0.21
**9** (500 nM)	3.85±1.48	49.75±5.59	45.80±3.82^*^	4.50±1.84
**32** (500 nM)	2.14±0.25	47.00±3.96^*^	49.80±3.25^*^	3.20±0.71^*^
**33** (500 nM)	1.98±0.21	51.80±0.14^**^	42.80±2.69^*^	5.35±2.76

a These data indicate the percentage of cells in G_0_/G_1_, S, and G_2_/M phases of the cell cycle. Each value is the mean ± SD of three determinations. (*, p<0.05 compared with control; **, p<0.01 compared with control).

The SubG_0_/G_1_ content of DNA is typically indicative of apoptosis. Form the flow cytometric analysis, we can see the proportion of cells in SubG_0_/G_1_ phase didn't differ significantly between the control and the treatment groups with 500 nM of these compounds for 24 h in A549 cells.

**1**, **9**, **32**, and **33** blocked S-G_2_ transition, resulting in S-phase accumulation, thereby delaying the progression of cells through G_2_/M phase in A549 cells. This result is consistent with the expression levels of cyclin A/CDK2 detected by Western blot assay, since this complex is the primary promoter of the G_2_ phase. Thus we speculated that the S phase blockage of cell cycle progression might be another important determinant in the growth inhibition of deoxytylophorinine and its derivatives.

## Materials and Methods

### Synthesis

THF was distilled from sodium and benzophenone, acetonitrile was distilled from 4 Å molecular sieves, and CH_2_Cl_2_ was distilled from P_2_O_5_. All the above distillations were performed under a nitrogen atmosphere. Triethylamine and pyridine were dried, distilled, and kept over KOH. Other solvents and reagents were used directly without any purification. ^1^H-NMR and ^13^C-NMR spectra were collected by using Varian Inova-500 (500 MHz) and Mercury Plus 400 (400 MHz) spectrometers (Figure S3). Abbreviations used in NMR analysis are as follows: s = singlet, d = doublet, dd = double-doublet, ddd = double-double-doublet, t = triplet, m = multiplet, brs = broad singlet, brd = broad doublet, brdd = broad double-doublet. ESI-MS spectra were recorded with Agilent LC/MSD TOF spectrometer. High-resolution mass spectra were obtained with an AccuTOF-CS (JMS-T100CS, JEOL) spectrometer. Optical rotations were collected on a Perkin-Elmer 343 polarimeter. Purities of screening compounds were assessed by HPLC [Xtimate C_18_, 4.6×250 mm, 5 µm] (Figure S4). All of the final products were purified by recrystallization with chemical purities over 95% and *ee* vuales of these compounds were determined by HPLC analysis on a chiral AD-H column [CHIRALPAK AD-H, 4.6×250 mm, 5 µm].

### Stilbene acid (a)

The mixture of homoveratric acid (19.60 g, 0.10 mol), p-anisaldehyde (12 mL, 0.10 mol), triethylamine (16 mL, 0.12 mol) and acetic anhydride (24 mL, 0.12 mol) was heated to reflux for 10 h, then cooled, filtered, and the product was washed with small portions of EtOAc to give yellow solid **a** (22.60 g, 72%). mp.207–208°C. ^1^H-NMR (500 MHz, CDCl_3_): 7.88 (1H, s), 7.06 (2H, d, *J* = 8.5 Hz), 6.71 (2H, d, *J* = 8.5 Hz), 6.76 (1H, brs), 6.81 (1H, brd, *J* = 8.0 Hz), 6.91 (1H, d, *J* = 8.0 Hz), 3.92 (3H, s), 3.81 (3H, s), 3.77 (3H, s).

### Esterification of Stilbene acid (b)

Acid **a** (17.60 g, 0.06 mol) was resolved in SOCl_2_ (15 mL, 0.08 mol) and the reaction mixture was refluxed for 30 min. After evaporation of the solvent, pyridine (4.53 mL, 0.06 mol) was added and then 30 mL of MeOH was added dropwise with stirring in an ice bath for 30 min. The product **b** was obtained after filtration and washed with small portions of MeOH. (14.73 g. 80%). mp 111–112°C. ^1^H-NMR (500 MHz, CDCl_3_): 7.77 (1H, s), 7.03 (2H, d, *J* = 8.5 Hz), 6.69 (2H, d, *J* = 8.5 Hz), 6.90 (1H, d, *J* = 8.0 Hz), 6.78 (1H, brd, *J* = 8.0 Hz), 6.74 (1H, brs), 3.93 (3H, s), 3.80 (3H, s), 3.79 (3H, s), 3.76 (3H, s).

### Methyl 3, 6, 7-Trimethoxyphenanthrene-9-carboxylate (c)

To a solution of **b** (10.17 g, 0.03 mol) in CH_2_Cl_2_ was added anhydrous FeCl_3_ (16.90 g, 0.11 mol) in one portion under an ice bath. After being stirred for 12 h at room temperature, the reaction was quenched with saturated NaHCO_3_ solvent and the organic layer was collected after filtration and partition. Then the organic layer was dried, evaporated and the product was recrystallized from MeOH to give yellow globules. (4.50 g. 44%). mp 151–152°C. ^1^H-NMR (400 MHz, CDCl_3_): 8.61 (1H, s), 8.37 (1H, s), 7.77 (1H, s), 7.71 (1H, d, *J* = 2.5 Hz), 7.78 (1H, d, *J* = 9.0 Hz), 7.16 (1H, dd, *J* = 9.0 Hz, 2.5 Hz), 4.07 (3H, s), 4.06 (3H, s), 3.99 (3H, s), 3.98 (3H, s).

### 9-(Hydroxymethyl)-3, 6, 7-trimethoxyphenanthren (d)

To a solution of LiAlH_4_ (1.00 g, 0.03 mol) in THF was added the solution of **c** (4.25 g, 0.01 mol) at 0°C. After being stirred for 30 min at 20°C, the reaction was terminated with THF/H_2_O (1∶1). The mixture was filtered, dried, and then evaporated. And the resulting white solid was recrystallized from MeOH to give 3.60 g product **d** as white needles with 93% yield. mp 155–156°C. ^1^H-NMR (400 MHz, CDCl_3_): 7.88 (1H, s), 7.58 (1H, s), 7.53 (1H, s), 7.81 (1H, d, *J* = 2.0 Hz), 7.75 (1H, d, *J* = 9.0 Hz), 7.19 (1H, dd, *J* = 9.0 Hz, 2.0 Hz), 5.09 (2H, s), 4.10 (3H, s), 4.05 (3H, s), 4.01 (3H, s).

### (*S*)-(+)-*N*-[(3, 6, 7-trimethoxy-9-phenanthryl)-methyl]pyroglutamic acid (g)

To the solution of **d** (4.26 g, 0.01 mol) and NaI (4.25 g, 0.03 mol) in 250 mL of acetonitrile was added TMSCl (2.44 mL, 0.02 mol) with stirring at room temperature. **e** was precipitated in 10 min and then L-diisopropyl glutamate (6.56 g, 0.03 mol) and K_2_CO_3_ (4.30 g, 0.03 mol) were added. After stirring for another 5 h, the suspension was evaporated, and the residue was partitioned between water and CH_2_Cl_2_ (100 mL×2). The organic layer was separated, dried, and evaporated to give crude **f** as brown oil. Then acetic acid (50 mL) and MeOH (100 mL) were added to the resulting oil and the mixture was stirred at 50°C for 5 h. After evaporation, the resulting residue was dissolved in 1, 4-dioxane (22 mL), MeOH (17 mL) and 2N KOH solution (11 mL, 0.02 mol). Upon completion of addition, the reaction mixture was stirred at room temperature for 1 h, and evaporated, and the residue was partitioned between water and CH_2_Cl_2_ (50 mL×2). The aqueous layer was acidified to pH 4 with H_3_PO_4_. The product of **g** was filtered and recrystallized from MeOH as white needles (3.10 g, 53% over four steps). mp 275–277°C. ^1^H-NMR (400 MHz, CDCl_3_): 7.90 (1H, s), 7.84 (1H, d, *J* = 2.0 Hz), 7.76 (1H, d, *J* = 8.8 Hz), 7.61 (1H, s), 7.49 (1H, s), 7.19 (1H, dd, *J* = 8.8 Hz, 2.0 Hz), 5.61 (1H, d, *J* = 14.4 Hz), 4.35 (1H, d, *J* = 14.4 Hz), 4.11 (3H, s), 4.04 (3H, s), 4.01 (3H, s), 3.87 (1H, m), 2.63 (1H, m), 2.42 (1H, m), 2.08–2.15 (2H, m).

### (*S*)-3, 6, 7-trimethoxyphenanthro[9, 10-*b*]-11, 14-indolizidinedione (h)

To the solution of **g** (3.32 g, 0.01 mol) in trifluoroacetic anhydride (10 mL, 0.07 mol) was added BF_3_·Et_2_O (20 mL, 0.16 mol) and the mixture was stirred for 3 h before poured into 50 mL of saturated NH_4_Cl solution. 200 mL of CH_2_Cl_2_ was added to dissolve the resulting solid. The organic layer was separated and washed with saturated NaHCO_3_ and H_2_O sequentially, dried, evaporated, and washed with EtOAc to give **h** as yellow powder (2.86 g, 90%). mp 249–251°C. [α]20 D +156 (*c* 1.0, CH_2_Cl_2_). ^1^H-NMR (400 MHz, CDCl_3_): 9.30 (1H, d, *J* = 9.2 Hz), 7.87 (1H, s), 7.77 (1H, d, *J* = 2.4 Hz), 7.26 (1H, dd, *J* = 9.2 Hz, 2.4 Hz), 7.18 (1H, s), 5.6 (1H, d, *J* = 18.0 Hz), 4.3 (1H, d, *J* = 18.0 Hz), 4.35 (1H, m), 4.13 (3H, s), 4.06 (3H, s), 4.01 (3H, s), 2.50–2.59 (4H, m).

### (*S*)-3, 6, 7-trimethoxyphenanthro[9, 10-*b*]-11-indolizidinone (j)

To a solution of **h** (0.45 g, 1.13 mmol) in a mixture of CH_2_Cl_2_ (10 mL) and MeOH (10 mL) was added NaBH_4_ (0.10 g, 2.26 mmol). After stirring for 30 min, 20 mL of saturated NH_4_Cl solution was poured into the reactant mixture with stirring. The organic layer was separated, dried, and evaporated to give **i**. The mixture of **i**, Et_3_SiH (2 mL, 12.50 mmol) and BF_3_·Et_2_O (8 mL, 64.80 mmol) was intensively stirred for 6 h at room temperature. 10 mL of saturated NH_4_Cl solution was added slowly to quench the reaction and then 20 mL of CH_2_Cl_2_ was added. The organic layer was dried and evaporated. The residue was washed with hot EtOH to give **j** (0.37 g, 85%). mp.216–218°C. [α]20 D +162 (*c* 0.2, CH_2_Cl_2_). ^1^H-NMR (500 MHz, CDCl_3_): 7.90 (1H, d, *J* = 9.0 Hz), 7.21 (1H, dd, *J* = 9.0 Hz, 2.0 Hz), 7.88 (1H, d, *J* = 2.0 Hz), 7.90 (1H, s), 7.12 (1H, s), 5.25 (1H, d, *J* = 17.0 Hz), 4.50 (1H, d, *J* = 17.0 Hz), 4.11 (3H, s), 4.04 (3H, s), 4.02 (3H, s), 3.88 (1H, m), 3.51 (1H, m), 2.79 (1H, m), 2.52–2.59 (3H, m), 1.98 (1H, m).

### (+)-13a-(*S*)-deoxytylophorinine (1)

To the solution of **j** (0.08 g, 0.21 mmol) in 10 mL of THF was added LiAlH_4_ (0.01 g, 0.26 mmol) under the protection of argon atmosphere. The mixture was heated to reflux and stirred for 2 h in dark. Then the reaction mixture was cooled to room temperature and added THF/H_2_O (1∶1) dropwise to quench the reaction, then filtered, dried, and evaporated. The product of **1** (0.06 g, 71%) was obtained by recrystallization from acetone/MeOH (1∶1) as white needles. mp.219–221°C. [α]20 D +102 (*c* 0.25, CHCl_3_). 99% *ee* [flow rate 1.0 mL/min, 18% isopropanol/hexane and 0.2% Et_3_N, t_R_ (major) = 23.81 min, t_R_ (minor) = 31.22 min]. ESI-MS: 364.2 [M+H]^+^. ^1^H-NMR (500 MHz, CDCl_3_): *δ* 7.92 (1H, d, *J* = 9.0 Hz, H-1), 7.20 (1H, dd, *J* = 9.0 Hz, 2.5 Hz, H-2), 7.87 (1H, d, *J* = 2.5 Hz, H-4), 7.89 (1H, s, H-5), 7.12 (1H, s, H-8), 4.57 (1H, d, *J* = 15.0 Hz, Ha-9), 3.61 (1H, d, *J* = 15.0 Hz, Hb-9), 4.09 (3H, s, CH_3_O-6), 4.04 (3H, s, CH_3_O-7), 4.00 (3H, s, CH_3_O-3), 3.46 (1H, m, Ha-11), 3.38 (1H, dd, *J* = 16.0 Hz, 2.5 Hz, Ha-14), 2.91 (1H, dd, *J* = 16.0 Hz, 13.5 Hz, Hb-14), 2.41–2.47 (2H, m, H-13a, Hb-11), 2.21 (1H, m, Ha-13), 2.02 (1H, m, Ha-12), 1.91 (1H, m, Hb-12), 1.75 (1H, m, Hb-13). ^13^C-NMR (125 MHz, CDCl_3_): *δ* 157.52, 149.38, 148.23, 130.33, 126.97, 125.59, 125.54, 125.27, 125.10, 123.30, 114.74, 104.55, 103.95, 103.14, 60.13, 55.96, 55.88, 55.47, 55.13, 53.93, 33.55, 31.22, 21.59. HRESIMS calcd for [M+H]^+^ C_23_H_26_NO_3_ 364.1913, found 364.1925. The purity was 99.8% determined by HPLC [flow rate 1.0 mL/min, 38% MeCN/H_2_O (0.06 M NH_4_H_2_PO_4_, 0.2% Et_3_N, 2.5% THF)].

### General Procedures for Synthesis of 2, 3, 4, 5, 6, 7

These derivatives were prepared from different substituted phenanthrene-9-carboxylic ester and L- or D-glutamate. The same reaction procedures and conditions were used as the synthesis of (+)-deoxytylophorinine.

### (−)-13a-(*R*)-deoxytylophorinine (2)

7% total yield. White needles (from acetone/MeOH (1∶1)). mp 219–221°C. [α]20 D −114 (*c* 0.25, CHCl_3_). 99% *ee* [flow rate 1.0 mL/min, 18% isopropanol/hexane and 0.2% Et_3_N, t_R_ (major) = 31.30 min, t_R_ (minor) = 24.00 min]. ESI-MS: 364.2 (M+H)^+^. ^1^H-NMR (400 MHz, CDCl_3_): *δ* 7.92 (1H, d, *J* = 9.2 Hz, H-1), 7.21 (1H, dd, *J* = 9.2 Hz, 2.0 Hz, H-2), 7.88 (1H, d, *J* = 2.0 Hz, H-4), 7.90 (1H, s, H-5), 7.12 (1H, s, H-8), 4.62 (1H, d, *J* = 14.8 Hz, Ha-9), 3.71 (1H, d, *J* = 14.8 Hz, Hb-9), 4.09 (3H, s, CH_3_O-6), 4.04 (3H, s CH_3_O-7), 4.00 (3H, s, CH_3_O-3), 3.45 (1H, m, Ha-11), 3.41 (1H, dd, *J* = 16.0 Hz, 2.8 Hz, Ha-14), 2.98 (1H, dd, *J* = 16.0 Hz, 10.8 Hz, Hb −14), 2.52–2.61 (2H, m, H-13a, Hb-11), 2.25 (1H, m, Ha-13), 2.05 (1H, m, Ha-12), 1.94 (1H, m, Hb-12), 1.80 (1H, m, Hb-13). ^13^C-NMR (100 MHz, CDCl_3_): *δ* 157.61, 149.42, 148.30, 130.39, 126.78, 125.42, 125.41, 125.21, 125.11, 123.34, 114.83, 104.55, 103.92, 103.02, 60.15, 55.96, 55.90, 55.48, 54.91, 53.46, 33.03, 31.03, 21.53. HRESIMS calcd for [M+H]^+^ C_23_H_26_NO_3_ 364.1913, found 364.1920. HPLC purity: 99.9% [flow rate 1.0 mL/min, 38% MeCN/H_2_O (0.06 M NH_4_H_2_PO_4_, 0.2% Et_3_N, 2.5% THF)].

### (*S*)-2, 3, 6, 7-tetramethoxyphenanthro[9, 10-*b*]-indolizidine [(+)-Tylophorine] (3)

7% total yield. White needles (from acetone/MeOH (1∶1)). mp 284–286°C. [α]20 D +73 (*c* 0.1, CH_2_Cl_2_). 93% *ee* [flow rate 1.0 mL/min, 26% isopropanol/hexane and 0.2% Et_3_N, t_R_ (major) = 10.28 min, t_R_ (minor) = 13.16 min]. ^1^H-NMR (500 MHz, CDCl_3_): *δ* 7.82 (2H, brs), 7.31 (1H, s), 7.15 (1H, s), 4.11 (6H, s), 4.06 (3H, s), 4.05 (3H, s), 4.63 (1H, d, *J* = 14.5 Hz), 3.66 (1H, d, *J* = 14.5 Hz), 3.48 (1H, brs), 3.37 (1H, brd, *J* = 16.0 Hz), 2.93 (1H, d, *J* = 16.0 Hz, 10.5 Hz), 2.47–2.50 (2H, m), 2.23 (1H, m), 2.05 (1H, m), 1.94 (1H, m), 1.79 (1H, m). HRESIMS calcd for [M+H]^+^ C_24_H_28_NO_4_ 394.2013, found 394.2026. HPLC purity: 98.6% [flow rate 1.0 mL/min, 46% MeCN/H_2_O (0.08 M NH_4_H_2_PO_4_, 0.2% Et_3_N)].

### (*S*)-2, 3, 6-trimethoxyphenanthro[9, 10-*b*]-indolizidine [(+)-Antofine] (4)

6% total yield. White needles (from acetone). mp 215–217°C. [α]20 D +102 (*c* 0.1, CH_2_Cl_2_). 97% *ee* [flow rate 1.0 mL/min, 26% isopropanol/hexane and 0.2% Et_3_N, t_R_ (major) = 14.49 min, t_R_ (minor) = 21.27 min]. ^1^H-NMR (500 MHz, CDCl_3_): *δ* 7.91 (1H, s), 7.90 (1H, d, *J* = 2.0 Hz), 7.80 (1H, d, *J* = 9.0 Hz), 7.20 (1H, dd, *J* = 9.0 Hz, 2.0 Hz), 7.30 (1H, s), 4.70 (1H, d, *J* = 15.0 Hz), 3.70 (1H, d, *J* = 15.0 Hz), 4.10 (3H, s), 4.06 (3H, s), 4.01 (3H, s), 3.47 (1H, brs), 3.34 (1H, brd, *J* = 15.5 Hz), 2.91 (1H, dd, *J* = 15.5 Hz, 10.5 Hz), 2.44–2.53 (2H, m), 2.24 (1H, m), 2.04 (1H, m), 1.93 (1H, m), 1.79 (1H, m). HRESIMS calcd for [M+H]^+^ C_23_H_26_NO_3_ 364.1907, found 364.1918. HPLC purity: 99.1% [flow rate 1.0 mL/min, 46% MeCN/H_2_O (0.08 M NH_4_H_2_PO_4_, 0.2% Et_3_N)].

### (*S*)-2, 6, 7-trimethoxyphenanthro[9, 10-*b*]-indolizidine (5)

5% total yield. White solid (from acetone). mp 198–200°C. [α]20 D +97 (*c* 0.1, CH_2_Cl_2_). 100% *ee* [flow rate 1.0 mL/min, 26% isopropanol/hexane and 0.2% Et_3_N, t_R_ = 25.08 min]. ^1^H-NMR (400 MHz, CDCl_3_): *δ* 8.43 (1H, d, *J* = 9.2 Hz), 7.21 (1H, dd, *J* = 9.2 Hz, 2.8 Hz), 7.31 (1H, d, *J* = 2.8 Hz), 7.90 (1H, s), 7.12 (1H, s), 4.59 (1H, d, *J* = 14.8 Hz), 3.61 (1H, d, *J* = 14.8 Hz), 4.08 (3H, s), 4.03 (3H, s), 3.96 (3H, s), 3.46 (1H, m), 3.31 (1H, dd, *J* = 15.6 Hz, 2.8 Hz), 2.87 (1H, dd, *J* = 15.6 Hz, 10.4 Hz), 2.39–2.46 (2H, m), 2.21 (1H, m), 2.02 (1H, m), 1.91 (1H, m), 1.75 (1H, m). HRESIMS calcd for [M+H]^+^ C_23_H_26_NO_3_ 364.1907, found 364.1916. HPLC purity: 99.2% [flow rate 1.0 mL/min, 46% MeCN/H_2_O (0.08 M NH_4_H_2_PO_4_, 0.2% Et_3_N)].

### (*S*)-3-fluoro-6, 7-dimethoxyphenanthro[9, 10-*b*]-indolizidine (6)

9% total yield. Light yellow solid (from acetone/MeOH (1∶1)). mp 190–192°C. [α]20 D +92 (*c* 0.1, CH_2_Cl_2_). 92% *ee* [flow rate 1.0 mL/min, 26% isopropanol/hexane and 0.2% Et_3_N, t_R_ (major) = 9.29 min, t_R_ (minor) = 13.24 min]. ^1^H-NMR (500 MHz,CDCl_3_): *δ* 8.11 (1H, brd, *J* = 11.5 Hz), 7.98 (1H, dd, *J* = 8.5 Hz, 6.0 Hz), 7.29 (1H, ddd, *J* = 9.0 Hz, 8.5 Hz, 2.0 Hz), 7.85 (1H, s), 7.16 (1H, s), 4.60 (1H, d, *J* = 15.0 Hz), 3.66 (1H, d, *J* = 15.0 Hz), 4.10 (3H, s), 4.06 (3H, s), 3.47 (1H, m), 3.41 (1H, brd, *J* = 16.0 Hz), 2.95 (1H, dd, *J* = 16.0 Hz, 10.5 Hz), 2.45–2.48 (2H, m), 2.24 (1H, m), 2.03 (1H, m), 1.93 (1H, m), 1.76 (1H, m). HRESIMS calcd for [M+H]^+^ C_22_H_23_FNO_2_ 352.1707, found 352.1719. HPLC purity: 98.4% [flow rate 1.0 mL/min, 46% MeCN/H_2_O (0.08 M NH_4_H_2_PO_4_, 0.2% Et_3_N)].

### (*S*)- 6, 7-dimethoxyphenanthro[9, 10-*b*]-indolizidine (7)

8% total yield. White solid (from acetone/MeOH (1∶1)). mp 196–198°C. [α]20 D +112 (*c* 0.1, CH_2_Cl_2_). 90% *ee* [flow rate 1.0 mL/min, 26% isopropanol/hexane and 0.2% Et_3_N, t_R_ (major) = 13.70 min, t_R_ (minor) = 24.50 min]. ^1^H-NMR (400 MHz, CDCl_3_): *δ* 8.53 (1H, d, *J* = 7.6 Hz), 8.03 (1H, s), 8.01 (H, d, *J* = 7.6 Hz), 7.57 (2H, brdd, *J* = 7.6 Hz, 7.6 Hz), 7.16 (1H, s), 4.62 (1H, d, *J* = 14.8 Hz), 3.66 (1H, d, *J* = 14.8 Hz), 4.10 (3H, s), 4.05 (3H, s), 3.43–3.49 (2H, m), 2.97 (1H, dd, *J* = 15.6 Hz, 10.8 Hz), 2.43–2.49 (2H, m), 2.23 (1H, m), 2.03 (1H, m), 1.92 (1H, m), 1.77 (1H, m). HRESIMS calcd for [M+H]^+^ C_22_H_24_NO_2_ 334.1802, found 334.1808. HPLC purity: 99.9% [flow rate 1.0 mL/min, 46% MeCN/H_2_O (0.08 M NH_4_H_2_PO_4_, 0.2% Et_3_N)].

### (13a*S*, 14*R*)-14-methoxy-3, 6, 7-trimethoxyphenanthro[9, 10-*b*]-11-indolizidinone (k) and (13a*S*, 14*S*)-14-methoxy-3, 6, 7-trimethoxyphenanthro[9, 10-*b*] -11-indolizidinone (l)

To the solution of **i** (0.20 g, 0.51 mmol) in 5 mL of THF was added NaH (70%, 0.07 g, 2.04 mmol) under protection of nitrogen gas. The mixture was stirred at room temperature for 30 min and then CH_3_I (0.16 mL, 3.15 mmol) was added. The reaction was continued for another 5 h and quenched by 10 mL of saturated NH_4_Cl solution. The organic layer was washed with water and dried. After evaporation the residue was isolated by silica gel column chromatography eluting with CH_2_Cl_2_/MeOH (50∶1) to give **k** (0.06 g, 29%)and **l** (0.08 g, 39%) respectively.

#### k

^1^H-NMR (500 MHz, CDCl_3_): *δ* 8.21 (1H, d, *J* = 9.0 Hz), 7.22 (1H, dd, *J* = 9.0 Hz, 2.0 Hz), 7.88 (1H, d, *J* = 2.0 Hz), 7.91 (1H, s), 7.21 (1H, s), 5.38 (1H, d, *J* = 17.0 Hz), 4.43 (1H, d, *J* = 17.0 Hz), 5.15 (1H, d, *J* = 7.0 Hz), 4.07 (1H, m), 4.12 (3H, s), 4.06 (3H, s), 4.02 (3H, s), 3.19 (3H, s), 2.66 (1H, m), 2.58 (2H, m), 2.24 (1H, m). NOEs were observed between H-14 (*δ*_H_ 5.15) and H-13 (*δ*_H_ 2.23), CH_3_O-14 (*δ*_H_ 3.19), and H-1 (*δ*_H_ 8.21).

#### l

^1^H-NMR (500 MHz, CDCl_3_): *δ* 8.19 (1H, d, *J* = 9.0 Hz), 7.27 (1H, dd, *J* = 9.0 Hz, 2.0 Hz), 7.92 (1H, d, *J* = 2.0 Hz), 7.93 (1H, s), 7.26 (1H, s), 5.36 (1H, d, *J* = 17.5 Hz), 4.63 (1H, d, *J* = 17.5 Hz), 5.19 (1H, brs), 3.96 (1H, m), 4.12 (3H, s), 4.06 (3H, s), 4.02 (3H, s), 3.16 (3H, s), 2.68 (1H, m), 2.60–2.51 (2H, m), 2.32 (1H, m). NOEs were observed between H-14 (*δ*_H_ 5.19) and CH_3_O-14 (*δ*_H_ 3.16), H-13a (*δ*_H_ 3.96), and H-1 (*δ*_H_ 8.19).

### (13a*S*, 14*R*)-14-methoxy-3, 6, 7-trimethoxyphenanthro[9, 10-*b*]-indolizidine (8)

To the solution of **k** (0.08 g, 0.20 mmol) in 5 mL of THF was added LiAlH_4_ (0.04 g, 1.05 mmol) under nitrogen gas. The mixture was heated to reflux and continued for 2 h in dark and then cooled to room temperature. THF/H_2_O (1∶1) was added slowly to the reactant until no bubble evolved. The mixture was filtered and the filtrate was dried and evaporated. The residue was recrystallized from MeOH to gave **8** (0.06 g, 78%) as white solid. mp 208–210°C (decomposed). [α]20 D +88 (*c* 0.1, CH_2_Cl_2_). 92% *ee* [flow rate 1.0 mL/min, 8% isopropanol/hexane and 0.2% Et_3_N, t_R_ (major) = 10.32 min, t_R_ (minor) = 18.27 min]. ^1^H-NMR (500 MHz, C_5_D_5_N): *δ* 8.50 (1H, d, *J* = 9.0 Hz), 7.42 (1H, brd, *J* = 9.0 Hz), 8.33 (2H, brs), 7.44 (1H, s), 5.26 (1H, d, *J* = 8.0 Hz), 4.68 (1H, d, *J* = 14.5 Hz), 3.71 (1H, d, *J* = 14.5 Hz), 3.98 (3H, s), 3.94 (3H, s), 3.90 (3H, s), 3.34 (3H, s), 3.25 (1H, brt, *J* = 7.5 Hz), 2.68 (1H, m), 2.38–2.33 (2H, m), 1.94–1.84 (2H, m), 1.76 (1H, m). ^13^C-NMR (125 MHz, C_5_D_5_N): *δ* 158.24, 150.57, 150.10 (overlapped), 131.62, 130.03, 128.53, 128.31, 125.68 (×2), 124.95, 115.74, 105.22, 104.99, 104.85, 81.00, 64.85, 56.00, 55.82, 55.41, 54.61, 54.22, 54.11, 31.77, 22.45. HRESIMS calcd for [M+H]^+^ C_24_H_28_NO_4_ 394.2013, found 394.2024. HPLC purity: 99.9% [flow rate 1.0 mL/min, 46% MeCN/H_2_O (0.08 M NH_4_H_2_PO_4_, 0.2% Et_3_N)].

### (13a*S*, 14*S*)-14-methoxy-3, 6, 7-trimethoxyphenanthro[9, 10-*b*]-indolizidine (9)

The same reaction procedures and conditions were involved as the synthesis of **8**. 69% yield. White globules (from MeOH). mp 173–175°C. [α]20 D +73 (*c* 0.1, CH_2_Cl_2_). 100% *ee* [flow rate 1.0 mL/min, 26% isopropanol/hexane and 0.2% Et_3_N, t_R_ = 17.57 min]. ^1^H-NMR (500 MHz, C_5_D_5_N): *δ* 8.63 (1H, d, *J* = 9.0 Hz), 7.46 (1H, brd, *J* = 9.0 Hz), 8.38 (2H, brs), 7.49 (1H, s), 5.28 (1H, brs), 4.86 (1H, d, *J* = 15.0 Hz), 3.54 (1H, d, *J* = 15.0 Hz), 4.01 (3H, s), 3.96 (3H, s), 3.92 (3H, s), 3.45 (3H, s), 3.33 (1H, brt, *J* = 7.5 Hz), 2.45–2.49 (2H, m), 2.21 (1H, m), 1.90–1.93 (2H, m), 1.74 (1H, m). ^13^C-NMR (125 MHz, C_5_D_5_N): *δ* 158.54, 150.59, 150.10 (overlapped), 131.43, 130.16, 127.18, 126.42, 125.74, 125.44, 125.32, 116.21, 105.25, 104.94 (×2), 71.10, 65.70, 56.03, 55.85, 55.48 (×2), 54.57, 54.41, 24.67, 22.13. HRESIMS calcd for [M+H]^+^ C_24_H_28_NO_4_ 394.2013, found 394.2024. HPLC purity: 99.9% [flow rate 1.0 mL/min, 46% MeCN/H_2_O (0.08 M NH_4_H_2_PO_4_, 0.2% Et_3_N)].

### (13a*R*, 14*S*)-14-methoxy-3, 6, 7-trimethoxyphenanthro[9, 10-*b*]-indolizidine (10)

The same reaction procedures and conditions were involved as the synthesis of **8**. 66% yield. White solid (from MeOH). mp 208–210°C (decomposed). [α]20 D −86 (*c* 0.1, CH_2_Cl_2_). 99% *ee* [flow rate 1.0 mL/min, 8% isopropanol/hexane and 0.2% Et_3_N, t_R_ (major) = 18.38 min, t_R_ (minor) = 10.35 min]. ^1^H-NMR (500 MHz, C_5_D_5_N): *δ* 8.50 (1H, d, *J* = 9.0 Hz), 7.42 (1H, brd, *J* = 9.0 Hz), 8.33 (2H, brs), 7.43 (1H, s), 5.26 (1H, d, *J* = 8.0 Hz), 4.68 (1H, d, *J* = 14.5 Hz), 3.71 (1H, d, *J* = 14.5 Hz), 3.98 (3H, s), 3.94 (3H, s), 3.90 (3H, s), 3.34 (3H, s), 3.25 (1H, brt, *J* = 8.0 Hz), 2.69 (1H, m), 2.33–2.37 (2H, m), 1.84–1.94 (2H, m), 1.76 (1H, m). HRESIMS calcd for [M+H]^+^ C_24_H_28_NO_4_ 394.2013, found 394.2020. HPLC purity: 99.9% [flow rate 1.0 mL/min, 46% MeCN/H_2_O (0.08 M NH_4_H_2_PO_4_, 0.2% Et_3_N)].

### (13a*R*, 14*R*)-14-methoxy-3, 6, 7-trimethoxyphenanthro[9, 10-*b*]-indolizidine (11)

The same reaction procedures and conditions were involved as the synthesis of **8**. 72% yield. White globules (from MeOH). mp 173–175°C. [α]20 D −77 (*c* 0.1, CH_2_Cl_2_). 94% *ee* [flow rate 1.0 mL/min, 26% isopropanol/hexane and 0.2% Et_3_N, t_R_ (major) = 23.34 min, t_R_ (minor) = 18.16 min]. ^1^H-NMR (500 MHz, C_5_D_5_N): *δ* 8.63 (1H, d, *J* = 9.0 Hz), 7.46 (1H, dd, *J* = 9.0 Hz, 2.5 Hz), 8.39 (1H, s), 8.38 (1H, d, 2.5 Hz), 7.50 (1H, s), 5.29 (1H, brs), 4.86 (1H, d, *J* = 15.0 Hz), 3.54 (1H, d, *J* = 15.0 Hz), 4.01 (3H, s), 3.96 (3H, s), 3.92 (3H, s), 3.45 (3H, s), 3.33 (1H, brt, *J* = 7.5 Hz), 2.45–2.50 (2H, m), 2.21 (1H, m), 1.90–1.93 (2H, m), 1.75 (1H, m). HRESIMS calcd for [M+H]^+^ C_24_H_28_NO_4_ 394.2013, found 394.2021. HPLC purity: 99.6% [flow rate 1.0 mL/min, 46% MeCN/H_2_O (0.08 M NH_4_H_2_PO_4_, 0.2% Et_3_N)].

### General procedures for the preparation of 14-alkylamino substituted derivatives

The solution of **h** (0.50 mmol) in 10 mL of dry CH_2_Cl_2_ was cooled to −20°C. To this solution was added amine (0.1 mL) and TiCl_4_ (0.25 mmol) sequently under nitrogen gas. The mixture was stirred and warmed slowly to room temperature in 2 h. The reaction was continued for another 24 h and filtered. The filtrate was concentrated to 5 mL and added 5 mL of MeOH. NaBH_4_ (0.10 g) was added to the resulting solution and stirred for 30 min, then quenched with saturated NH_4_Cl solution. The organic layer was dried and evaporated. The residue was subjected to silica gel column chromatography eluting by CH_2_Cl_2_/MeOH (50∶1) to give **m** and **n** respectively. Reduction of **m** and **n** by LiAlH_4_ afforded target compounds. These compounds could be purified by recrystallization from MeOH or acetone/H_2_O (1∶1) mixture respectively.

### (13a*S*, 14*R*)-14-benzylamino-3, 6, 7-trimethoxyphenanthro[9, 10-*b*]-11-indolizidinone (m)

58% yield. White solid (from MeOH). ^1^H-NMR (500 MHz, CDCl_3_): *δ* 8.01 (1H, d, *J* = 9.0 Hz), 7.88 (1H, m), 7.89 (1H, s), 7.28–7.17 (7H, m), 5.45 (1H, d, *J* = 16.5 Hz), 4.37 (1H, d, *J* = 16.5 Hz), 4.55 (1H, d, *J* = 6.5 Hz), 4.09 (1H, overlapped), 4.11 (3H, s), 4.06 (3H, s), 4.02 (3H, s), 3.77 (1H, d, *J* = 12.5 Hz), 3.63 (1H, d, *J* = 12.5 Hz) 2.70 (1H, m), 2.56–2.60 (2H, m), 2.18 (1H, m).

### (13a*S*, 14*S*)-14-benzylamino-3, 6, 7-trimethoxyphenanthro[9, 10-*b*]-11-indolizidinone (n)

39% yield. White solid (from MeOH). ^1^H-NMR (500 MHz, CDCl_3_): *δ* 8.05 (1H, d, *J* = 9.5 Hz), 7.93 (2H, s), 7.28–7.19 (7H, m), 5.34 (1H, d, *J* = 18.0 Hz), 4.65 (1H, d, *J* = 18.0 Hz), 4.53 (1H, brs), 3.99 (1H, m), 4.12 (3H, s), 4.06 (3H, s), 4.05 (3H, s), 3.91 (1H, d, *J* = 12.5 Hz), 3.82 (1H, d, *J* = 12.5 Hz) 2.80 (1H, m), 2.55–2.61 (2H, m), 2.31 (1H, m).

### (13a*S*, 14*R*)-14-benzylamino-3, 6, 7-trimethoxyphenanthro[9, 10-*b*]-indolizidine (12)

82% yield. Light yellow cluster crystals (from MeOH). mp 192–194°C. [α]20 D +33 (*c* 0.1, CH_2_Cl_2_). 100% *ee* [flow rate 1.0 mL/min, 1% isopropanol/hexane and 0.2% Et_3_N, t_R_ = 67.62 min]. ^1^H-NMR (500 MHz, C_5_D_5_N): *δ* 8.67 (1H, d, *J* = 9.0 Hz), 7.43 (1H, dd, *J* = 9.0 Hz, 2.0 Hz), 8.33 (1H, d, *J* = 2.0 Hz), 8.35 (1H, s), 7.48 (1H, s), 7.47 (2H, d, *J* = 8.5 Hz), 7.30 (2H, t, *J* = 8.5 Hz), 7.22 (1H, t, *J* = 8.5 Hz), 4.73 (1H, d, *J* = 6.5 Hz), 4.70 (1H, d, *J* = 15.0 Hz), 3.76 (1H, d, *J* = 15.0 Hz), 3.98 (3H, s), 3.95 (3H, s), 3.90 (3H, s), 3.97 (1H, overlapped), 3.82 (1H, d, *J* = 12.5 Hz), 3.30 (1H, brt, *J* = 7.0 Hz), 2.70 (1H, m), 2.37–2.45 (2H, m), 1.88–1.92 (2H, m), 1.79 (1H, m). ^13^C-NMR (125 MHz, C_5_D_5_N): *δ* 158.17, 150.60, 150.10 (overlapped), 142.06, 131.84, 130.51, 129.34 (×4), 128.57, 128.36, 127.04, 126.07, 125.58, 124.59, 115.40, 105.31, 104.88 (×2), 68.07, 61.09, 56.06, 55.85, 55.40, 54.79, 54.17, 50.77, 32.29, 22.50. HRESIMS calcd for [M+H]^+^ C_30_H_33_N_2_O_3_ 469.2486, found 469.2502. HPLC purity: 98.2% [flow rate 1.0 mL/min, 46% MeCN/H_2_O (0.08 M NH_4_H_2_PO_4_, 0.2% Et_3_N)].

### (13a*S*, 14*S*)-14-benzylamino-3, 6, 7-trimethoxyphenanthro[9, 10-*b*]-indolizidine (13)

84% yield. Light yellow cluster crystals (from MeOH). mp 147–149°C. [α]20 D +46 (*c* 0.1, CH_2_Cl_2_). 100% *ee* [flow rate 1.0 mL/min, 26% isopropanol/hexane and 0.2% Et_3_N, t_R_ = 13.96 min]. ^1^H-NMR (500 MHz, C_5_D_5_N): *δ* 8.51 (1H, d, *J* = 9.0 Hz), 7.46 (1H, dd, overlapped), 8.39 (2H, brs), 7.47 (1H, s), 7.44 (2H, d, *J* = 7.5 Hz), 7.28 (2H, t, *J* = 7.5 Hz), 7.21 (1H, t, overlapped), 4.85 (1H, d, *J* = 15.0 Hz), 3.70 (1H, d, *J* = 15.0 Hz), 4.63 (1H, brs), 4.27 (1H, d, *J* = 14.0 Hz), 4.04 (1H, d, *J* = 14.0 Hz), 4.00 (3H, s), 3.94 (3H, s), 3.93 (3H, s), 3.37 (1H, brt, *J* = 7.5 Hz), 2.66 (1H, m), 2.46–2.54 (2H, m), 2.34 (1H, m), 1.85–1.96 (2H, m), 1.78 (1H, m). ^13^C-NMR (125 MHz, C_5_D_5_N): *δ* 158.34, 150.60, 150.10 (overlapped), 142.79, 131.53, 131.28, 128.51 (×2), 128.43 (×2), 127.17, 126.95, 126.87, 126.26, 125.99, 124.73, 115.75, 105.33, 104.98, 104.75, 65.62, 56.06, 55.84, 55.61, 55.47, 54.71, 53.24, 52.61, 26.07, 22.65. HRESIMS calcd for [M+Na]^+^ C_30_H_32_N_2_NaO_3_ 491.2311, found 491.2243. HPLC purity: 98.7% [flow rate 1.0 mL/min, 46% MeCN/H_2_O (0.08 M NH_4_H_2_PO_4_, 0.2% Et_3_N)].

### (13a*R*, 14*S*)-14-benzylamino-3, 6, 7-trimethoxyphenanthro[9, 10-*b*]-indolizidine (14)

79% yield. Light yellow cluster crystals (from MeOH). mp 192–194°C. [α]20 D −32 (*c* 0.1, CH_2_Cl_2_). 100% *ee* [flow rate 1.0 mL/min, 1% isopropanol/hexane and 0.2% Et_3_N, t_R_ = 62.37 min]. ^1^H-NMR (500 MHz, C_5_D_5_N): *δ* 8.67 (1H, d, *J* = 9.0 Hz), 7.43 (1H, brd, *J* = 9.0 Hz), 8.33 (1H, brs), 8.35 (1H, s), 8.48 (1H, s),7.47 (2H, d, *J* = 7.5 Hz), 7.30 (2H, t, *J* = 7.5 Hz), 7.22 (1H, t, *J* = 7.5 Hz), 4.73 (1H, d, *J* = 7.0 Hz), 4.70 (1H, d, *J* = 14.5 Hz), 3.76 (1H, d, *J* = 14.5 Hz), 3.98 (3H, s), 3.95 (3H, s), 3.90 (3H, s), 3.97 (1H, overlapped), 3.82 (1H, d, *J* = 13.0 Hz), 3.30 (1H, brt, *J* = 7.5 Hz), 2.70 (1H, m), 2.37–2.45 (2H, m), 1.88–1.92 (2H, m), 1.81 (1H, m). HRESIMS calcd for [M+H]^+^ C_30_H_33_N_2_O_3_ 469.2486, found 469.2498. HPLC purity: 99.0% [flow rate 1.0 mL/min, 46% MeCN/H_2_O (0.08 M NH_4_H_2_PO_4_, 0.2% Et_3_N)].

### (13a*R*, 14*R*)-14-benzylamino-3, 6, 7-trimethoxyphenanthro[9, 10-*b*]-indolizidine (15)

80% yield. Light yellow cluster crystals (from MeOH). mp 147–149°C. [α]20 D −47 (*c* 0.1, CH_2_Cl_2_). 100% *ee* [flow rate 1.0 mL/min, 26% isopropanol/hexane and 0.2% Et_3_N, t_R_ = 16.62 min]. ^1^H-NMR (500 MHz, C_5_D_5_N): *δ* 8.51 (1H, d, *J* = 9.0 Hz), 7.45 (1H, dd, overlapped), 8.40 (2H, brs), 7.48 (1H, s), 7.44 (2H, d, *J* = 7.5 Hz), 7.27 (2H, t, *J* = 7.5 Hz), 7.21 (1H, overlapped), 4.86 (1H, d, *J* = 15.0 Hz), 3.70 (1H, d, *J* = 15.0 Hz), 4.63 (1H, brs), 4.27 (1H, d, *J* = 14.0 Hz), 4.04 (1H, d, *J* = 14.0 Hz), 4.00 (3H, s), 3.94 (3H, s), 3.93 (3H, s), 3.37 (1H, brt, *J* = 7.5 Hz), 2.69 (1H, m), 2.46–2.54 (2H, m), 2.33 (1H, m), 1.85–1.96 (2H, m), 1.77 (1H, m). HRESIMS calcd for [M+H]^+^ C_30_H_33_N_2_O_3_ 469.2486, found 469.2502. HPLC purity: 99.8% [flow rate 1.0 mL/min, 46% MeCN/H_2_O (0.08 M NH_4_H_2_PO_4_, 0.2% Et_3_N)].

### (13a*S*, 14*R*)-14-propylamino-3, 6, 7-trimethoxyphenanthro[9, 10-*b*]-indolizidine (16)

69% yield. Light yellow needles (from MeOH). mp 129–131°C. [α]20 D +50 (*c* 0.1, CH_2_Cl_2_). 98% *ee* [flow rate 1.0 mL/min, 8% isopropanol/hexane and 0.2% Et_3_N, t_R_ (major) = 9.34 min, t_R_ (minor) = 17.18 min]. ^1^H-NMR (500 MHz, C_5_D_5_N): *δ* 8.59 (1H, d, *J* = 9.0 Hz), 7.44 (1H, dd, *J* = 9.0 Hz, 2.0 Hz), 8.33 (1H, d, *J* = 2.0 Hz), 8.34 (1H, s), 7.46 (1H, s), 4.69 (1H, d, *J* = 15.0 Hz), 3.71 (1H, d, *J* = 15.0 Hz), 4.55 (1H, d, *J* = 7.0 Hz), 3.98 (3H, s), 3.95 (3H, s), 3.91 (3H, s), 3.29 (1H, brt, *J* = 7.5 Hz), 2.69 (1H, m), 2.56–2.62 (2H, m), 2.34–2.39 (2H, m), 1.85–1.89 (2H, m), 1.77–1.79 (2H, m), 1.39–1.44 (2H, m), 0.83 (3H, t, *J* = 7.5 Hz). ^13^C-NMR (125 MHz, C_5_D_5_N): *δ* 158.13, 150.60, 150.10 (overlapped), 131.83, 130.99, 129.04, 128.35, 126.11, 125.69, 124.51, 115.33, 105.32, 104.90 (×2), 68.49, 61.32, 56.05, 55.85, 55.41, 54.77, 54.22, 48.81, 32.34, 24.48, 22.49, 12.18. HRESIMS calcd for [M+H]^+^ C_26_H_33_N_2_O_3_ 421.2485, found 421.2489. HPLC purity: 99.6% [flow rate 1.0 mL/min, 46% MeCN/H_2_O (0.08 M NH_4_H_2_PO_4_, 0.2% Et_3_N)].

### (13a*S*, 14*S*)-14-propylamino-3, 6, 7-trimethoxyphenanthro[9, 10-*b*]-indolizidine (17)

65% yield. Light yellow cluster crystals (from acetone/H_2_O (1∶1)). mp 137–139°C. [α]20 D +68 (*c* 0.1, CH_2_Cl_2_). 98% *ee* [flow rate 1.0 mL/min, 8% isopropanol/hexane and 0.2% Et_3_N, t_R_ (major) = 20.37 min, t_R_ (minor) = 38.33 min]. ^1^H-NMR (500 MHz, C_5_D_5_N): *δ* 8.51 (1H, d, *J* = 9.0 Hz), 7.51 (1H, dd, *J* = 9.0 Hz, 2.5 Hz), 8.38 (1H, d, *J* = 2.5 Hz), 8.37 (1H, s), 7.46 (1H, s), 4.86 (1H, d, *J* = 15.0 Hz), 3.67 (1H, d, *J* = 15.0 Hz), 4.47 (1H, brs), 3.99 (3H, s), 3.94 (3H, s), 3.93 (3H, s), 3.38 (1H, brt, *J* = 7.5 Hz), 3.03 (1H, m), 2.87 (1H, m), 2.64 (1H, m), 2.31–2.40 (2H, m), 1.86–1.95 (2H, m), 1.76 (1H, m), 1.41–1.45 (2H, m), 0.81 (3H, t, *J* = 7.5 Hz). ^13^C-NMR (125 MHz, C_5_D_5_N): *δ* 158.29, 150.57, 150.10 (overlapped), 131.93, 131.55, 127.11, 126.45, 126.29, 126.00, 124.62, 115.70, 105.33, 105.01, 104.73, 65.67, 56.04, 55.82, 55.62, 55.47, 54.76, 53.86, 51.41, 26.06, 24.89, 22.57, 12.13. HRESIMS calcd for [M+Na]^+^ C_26_H_32_N_2_NaO_3_ 443.2311, found 443.2308. HPLC purity: 99.8% [flow rate 1.0 mL/min, 46% MeCN/H_2_O (0.08 M NH_4_H_2_PO_4_, 0.2% Et_3_N)].

### (13a*R*, 14*S*)-14-propylamino-3, 6, 7-trimethoxyphenanthro[9, 10-*b*]-indolizidine (18)

**7**1% yield. Light yellow needles (from MeOH). mp 129–131°C. [α]20 D −50 (*c* 0.1, CH_2_Cl_2_). 100% *ee* [flow rate 1.0 mL/min, 8% isopropanol/hexane and 0.2% Et_3_N, t_R_ = 17.43 min]. ^1^H-NMR (500 MHz, C_5_D_5_N): *δ* 8.59 (1H, d, *J* = 9.0 Hz), 7.44 (1H, dd, *J* = 9.0 Hz, 2.0 Hz), 8.33 (1H, d, *J* = 2.0 Hz), 8.34 (1H, s), 7.46 (1H, s), 4.68 (1H, d, *J* = 15.0 Hz), 3.72 (1H, d, *J* = 15.0 Hz), 4.55 (1H, d, *J* = 7.0 Hz), 3.98 (3H, s), 3.95 (3H, s), 3.91 (3H, s), 3.28 (1H, brt, *J* = 7.5 Hz), 2.69 (1H, m), 2.56–2.62 (2H, m), 2.34–2.39 (2H, m), 1.85–1.89 (2H, m), 1.78 (1H, m), 1.40–1.44 (2H, m), 0.83 (3H, t, *J* = 7.5 Hz). HRESIMS calcd for [M+H]^+^ C_26_H_33_N_2_O_3_ 421.2486, found 421.2494. HPLC purity: 99.1% [flow rate 1.0 mL/min, 46% MeCN/H_2_O (0.08 M NH_4_H_2_PO_4_, 0.2% Et_3_N)].

### (13a*R*, 14*R*)-14-propylamino-3, 6, 7-trimethoxyphenanthro[9, 10-*b*]-indolizidine (19)

66% yield. Light yellow cluster crystals (from acetone/H_2_O (1∶1)). mp 137–139°C. [α]20 D −68 (*c* 0.1, CH_2_Cl_2_). 99% *ee* [flow rate 1.0 mL/min, 8% isopropanol/hexane and 0.2% Et_3_N, t_R_ (major) = 38.29 min, t_R_ (minor) = 20.97 min]. ^1^H-NMR (500 MHz, C_5_D_5_N): *δ* 8.51 (1H, d, *J* = 9.0 Hz), 7.51 (1H, dd, *J* = 9.0 Hz, 2.5 Hz), 8.38 (1H, d, *J* = 2.5 Hz), 8.37 (1H, s), 7.46 (1H, s), 4.86 (1H, d, *J* = 15.0 Hz), 3.67 (1H, d, *J* = 15.0 Hz), 4.47 (1H, brs), 3.99 (3H, s), 3.94 (3H, s), 3.93 (3H, s), 3.38 (1H, brt, *J* = 8.0 Hz), 3.03 (1H, m), 2.86 (1H, m), 2.63 (1H, m), 2.31–2.40 (2H, m), 1.86–1.95 (2H, m), 1.78 (1H, m), 1.39–1.46 (2H, m), 0.81 (3H, t, *J* = 7.5 Hz). HRESIMS calcd for [M+H]^+^ C_26_H_32_N_2_NaO_3_ 421.2486, found 421.2489. HPLC purity: 99.5% [flow rate 1.0 mL/min, 46% MeCN/H_2_O (0.08 M NH_4_H_2_PO_4_, 0.2% Et_3_N)].

### (13a*S*, 14*R*)-14-isopropylamino-3, 6, 7-trimethoxyphenanthro[9, 10-*b*]-indolizidine (20)

83% yield. White solid (from MeOH). mp 117–119°C. [α]20 D +47 (*c* 0.1, CH_2_Cl_2_). 98% *ee* [flow rate 1.0 mL/min, 8% isopropanol/hexane and 0.2% Et_3_N, t_R_ (major) = 7.58 min, t_R_ (minor) = 6.55 min]. ^1^H-NMR (500 MHz, C_5_D_5_N): *δ* 8.69 (1H, d, *J* = 9.0 Hz), 7.45 (1H, dd, *J* = 9.0 Hz, 2.0 Hz), 8.33 (1H, d, *J* = 2.0 Hz), 8.34 (1H, s), 7.46 (1H, s), 4.75 (1H, d, *J* = 7.0 Hz), 4.65 (1H, d, *J* = 14.5 Hz), 3.69 (1H, d, *J* = 14.5 Hz), 3.98 (3H, s), 3.95 (3H, s), 3.90 (3H, s), 3.16–3.25 (2H, m), 2.51 (1H, m), 2.31–2.39 (2H, m), 1.74–1.92 (3H, m), 1.27 (3H, d, *J* = 6.0 Hz), 0.96 (3H, d, *J* = 6.0 Hz). ^13^C-NMR (125 MHz, C_5_D_5_N): *δ* 158.02, 150.57, 150.10 (overlapped), 131.85, 131.31, 128.99, 128.85, 126.17, 125.77, 124.48, 115.11, 105.27, 104.87, 104.76, 70.00, 57.66, 56.03, 55.85, 55.42, 54.40, 53.61, 45.74, 32.96, 25.30, 22.59, 22.19. HRESIMS calcd for [M+H]^+^ C_26_H_33_N_2_O_3_ 421.2486, found 421.2496. HPLC purity: 97.7% [flow rate 1.0 mL/min, 46% MeCN/H_2_O (0.08 M NH_4_H_2_PO_4_, 0.2% Et_3_N)].

### (13a*S*, 14*S*)-14-isopropylamino-3, 6, 7-trimethoxyphenanthro[9, 10-*b*]-indolizidine (21)

71% yield. Light yellow amorphous solid (from acetone/H_2_O (1∶1)). mp 149–151°C. [α]20 D +65 (*c* 0.1, CH_2_Cl_2_). 100% *ee* [flow rate 1.0 mL/min, 8% isopropanol/hexane and 0.2% Et_3_N, t_R_ = 17.34 min]. ^1^H-NMR (500 MHz, C_5_D_5_N): *δ* 8.58 (1H, d, *J* = 9.0 Hz), 7.51 (1H, dd, *J* = 9.0 Hz, 2.0 Hz), 8.38 (1H, d, *J* = 2.0 Hz), 8.36 (1H, s), 7.46 (1H, s), 4.88 (1H, d, *J* = 15.0 Hz), 3.71 (1H, d, *J* = 15.0 Hz), 4.64 (1H, brs), 3.99 (3H, s), 3.94 (3H, s), 3.93 (3H, s), 3.33–3.38 (2H, m), 2.66 (1H, m), 2.49 (1H, m), 2.33 (1H, m), 1.77–1.92 (3H, m), 1.09 (3H, d, *J* = 6.0 Hz), 0.99 (3H, d, *J* = 6.0 Hz). ^13^C-NMR (125 MHz, C_5_D_5_N): *δ* 158.30, 150.60, 150.10 (overlapped), 133.32, 131.46, 127.08, 126.31, 126.27, 126.23, 124.55, 115.41, 105.31, 104.99, 104.68, 65.57, 56.04, 55.82, 55.75, 55.49, 54.61, 50.56, 45.83, 26.19, 25.19, 24.38, 22.87. HRESIMS calcd for [M+H]^+^ C_26_H_33_N_2_O_3_ 421.2486, found 421.2500. HPLC purity: 99.1% [flow rate 1.0 mL/min, 46% MeCN/H_2_O (0.08 M NH_4_H_2_PO_4_, 0.2% Et_3_N)].

### (13a*R*, 14*S*)-14-isopropylamino-3, 6, 7-trimethoxyphenanthro[9, 10-*b*]-indolizidine (22)

77% yield. White solid (from MeOH). mp 117–119°C. [α]20 D −43 (*c* 0.1, CH_2_Cl_2_). 99% *ee* [flow rate 1.0 mL/min, 8% isopropanol/hexane and 0.2% Et_3_N, t_R_ (major) = 6.48 min, t_R_ (minor) = 7.44 min]. ^1^H-NMR (500 MHz, C_5_D_5_N): *δ* 8.69 (1H, overlapped), 7.45 (1H, dd, overlapped), 8.33 (1H, d, *J* = 2.0 Hz), 8.34 (1H, s), 7.46 (1H, s), 4.74 (1H, d, *J* = 7.0 Hz), 4.65 (1H, d, *J* = 14.5 Hz), 3.69 (1H, d, *J* = 14.5 Hz), 3.98 (3H, s), 3.95 (3H, s), 3.91 (3H, s), 3.25–3.16 (2H, m), 2.50 (1H, m), 2.34–2.37 (2H, m), 1.78–1.88 (3H, m), 1.27 (3H, d, *J* = 6.0 Hz), 0.96 (3H, d, *J* = 6.0 Hz). HRESIMS calcd for [M+H]^+^ C_26_H_33_N_2_O_3_ 421.2486. found 421.2492. HPLC purity: 99.3% [flow rate 1.0 mL/min, 46% MeCN/H_2_O (0.08 M NH_4_H_2_PO_4_, 0.2% Et_3_N)].

### (13a*R*, 14*R*)-14-isopropylamino-3, 6, 7-trimethoxyphenanthro[9, 10-*b*]-indolizidine (23)

73% yield. Light yellow amorphous solid (from acetone/H_2_O (1∶1)). mp 149–151°C. [α]20 D −65 (*c* 0.1, CH_2_Cl_2_). 99% *ee* [flow rate 1.0 mL/min, 8% isopropanol/hexane and 0.2% Et_3_N, t_R_ (major) = 35.90 min, t_R_ (minor) = 17.83 min]. ^1^H-NMR (500 MHz, C_5_D_5_N): *δ* 8.58 (1H, d, *J* = 9.0 Hz), 7.51 (1H, dd, *J* = 9.0 Hz, 2.0 Hz), 8.38 (1H, d, *J* = 2.0 Hz), 8.36 (1H, s), 7.46 (1H, s), 4.88 (1H, d, *J* = 15.5 Hz), 3.71 (1H, d, *J* = 15.5 Hz), 4.64 (1H, brs), 3.99 (3H, s), 3.94 (3H, s), 3.93 (3H, s), 3.32–3.38 (2H, m), 2.66 (1H, m), 2.49 (1H, m), 2.33 (1H, m), 1.76–1.94 (3H, m), 1.09 (3H, d, *J* = 6.0 Hz), 0.99 (3H, d, *J* = 6.0 Hz). HRESIMS calcd for [M+H]^+^ C_26_H_33_N_2_O_3_ 421.2486. found 421.2488. HPLC purity: 98.3% [flow rate 1.0 mL/min, 46% MeCN/H_2_O (0.08 M NH_4_H_2_PO_4_, 0.2% Et_3_N)].

### (13a*S*, 14*R*)-14-cyclopentylamino-3, 6, 7-trimethoxyphenanthro[9, 10-*b*]-indolizidine (24)

78% yield. White needles (from MeOH). mp 150–152°C. [α]20 D +45 (*c* 0.1, CH_2_Cl_2_). 99% *ee* [flow rate 1.0 mL/min, 8% isopropanol/hexane and 0.2% Et_3_N, t_R_ (major) = 7.37 min, t_R_ (minor) = 6.23 min]. ^1^H-NMR (500 MHz, C_5_D_5_N): *δ* 8.68 (1H, d, *J* = 9.0 Hz), 7.46 (1H, dd, *J* = 9.0 Hz, 2.5 Hz), 8.33 (1H, d, *J* = 2.5 Hz), 8.34 (1H, s), 7.47 (1H, s), 4.68 (1H, overlapped), 4.67 (1H, d, *J* = 14 Hz), 3.72 (1H, d, *J* = 14 Hz), 3.98 (3H, s), 3.95 (3H, s), 3.91 (3H, s), 3.38 (1H, m), 3.26 (1H, brt, *J* = 7.5 Hz), 2.56 (1H, m), 2.35–2.39 (2H, m), 1.71–1.92 (6H, m), 1.49–1.63 (4H, m), 1.36 (1H, m), 1.21 (1H, m). ^13^C-NMR (125 MHz, C_5_D_5_N): *δ* 158.04, 150.58, 150.10 (overlapped), 131.85, 131.36, 129.00, 128.92, 126.18, 125.81, 124.49, 115.06, 105.29, 104.87, 104.73, 69.43, 59.19, 57.02, 56.03, 55.85, 55.42, 54.59, 53.79, 35.30, 32.86 (×2), 24.34, 23.99, 22.65. HRESIMS calcd for [M+H]^+^ C_28_H_35_N_2_O_3_ 447.2642, found 447.2650. HPLC purity: 99.0% [flow rate 1.0 mL/min, 46% MeCN/H_2_O (0.08 M NH_4_H_2_PO_4_, 0.2% Et_3_N)].

### (13a*S*, 14*S*)-14-cyclopentylamino-3, 6, 7-trimethoxyphenanthro [9, 10-*b*] -indolizidine (25)

74% yield. Light yellow solid (from acetone/H_2_O (1∶1)). mp 112–114°C. [α]20 D +73 (*c* 0.1, CH_2_Cl_2_). 99% *ee* [flow rate 1.0 mL/min, 8% isopropanol/hexane and 0.2% Et_3_N, t_R_ (major) = 16.13 min, t_R_ (minor) = 40.03 min]. ^1^H-NMR (500 MHz, C_5_D_5_N): *δ* 8.58 (1H, d, *J* = 9.0 Hz), 7.53 (1H, dd, *J* = 9.0Hz, 2.0 Hz), 8.39 (1H, d, *J* = 2.0 Hz), 8.37 (1H, s), 7.46 (1H, s), 4.88 (1H, d, *J* = 15.5 Hz), 3.72 (1H, d, *J* = 15.5 Hz), 4.58 (1H, brs), 3.99 (3H, s), 3.94 (3H, s), 3.93 (3H, s), 3.52 (1H, m), 3.36 (1H, brt, *J* = 7.5 Hz), 2.67 (1H, m), 2.49 (1H, m), 2.35 (1H, m), 1.83–1.96 (2H, m), 1.71–1.80 (2H, m), 1.50–1.68 (3H, m), 1.43–1.24 (5H, m). ^13^C-NMR (125 MHz, C_5_D_5_N): *δ* 158.20, 150.48, 150.10 (overlapped), 133.08, 131.27, 127.11, 126.28, 126.19, 126.08, 124.44, 115.29, 105.20, 104.82, 104.56, 65.36, 57.35, 55.94, 55.71, 55.64, 55.38, 54.48, 51.45, 35.56, 34.27, 25.94, 24.38, 24.17, 22.77. HRESIMS calcd for [M+H]^+^ C_28_H_35_N_2_O_3_ 447.2642, found 447.2653. HPLC purity: 98.9% [flow rate 1.0 mL/min, 46% MeCN/H_2_O (0.08 M NH_4_H_2_PO_4_, 0.2% Et_3_N)].

### (13a*R*, 14*S*)-14-cyclopentylamino-3, 6, 7-trimethoxyphenanthro[9, 10-*b*]-indolizidine (26)

81% yield. White needles (from MeOH). mp 150–152°C. [α]20 D −44 (*c* 0.1, CH_2_Cl_2_). 99% *ee* [flow rate 1.0 mL/min, 8% isopropanol/hexane and 0.2% Et_3_N, t_R_ (major) = 6.06 min, t_R_ (minor) = 7.68 min]. ^1^H-NMR (500 MHz, C_5_D_5_N): *δ* 8.68 (1H, d, *J* = 9.0 Hz), 7.46 (1H, overlapped), 8.32 (1H, brs), 8.34 (1H, s), 7.47 (1H, s), 4.68 (1H, overlapped), 4.67 (1H, d, *J* = 14.5 Hz), 3.72 (1H, d, *J* = 14.5 Hz), 3.98 (3H, s), 3.95 (3H, s), 3.91 (3H, s), 3.38 (1H, m), 3.26 (1H, brt, *J* = 7.5 Hz), 2.57 (1H, m), 2.38–2.40 (2H, m), 1.70–1.88 (6H, m), 1.49–1.64 (4H, m), 1.37 (1H, m), 1.21 (1H, m). HRESIMS calcd for [M+H]^+^ C_28_H_35_N_2_O_3_ 447.2642, found 447.2650. HPLC purity: 98.5% [flow rate 1.0 mL/min, 46% MeCN/H_2_O (0.08 M NH_4_H_2_PO_4_, 0.2% Et_3_N)].

### (13a*R*, 14*R*)-14-cyclopentylamino-3, 6, 7-trimethoxyphenanthro[9, 10-*b*]-indolizidine (27)

67% yield. Light yellow solid (from acetone/H_2_O (1∶1)). mp 112–114°C. [α]20 D −73 (*c* 0.1, CH_2_Cl_2_). 100% *ee* [flow rate 1.0 mL/min, 8% isopropanol/hexane and 0.2% Et_3_N, t_R_ = 40.11 min]. ^1^H-NMR (500 MHz, C_5_D_5_N): *δ* 8.58 (1H, d, *J* = 9.0 Hz), 7.53 (1H, overlapped), 8.39 (1H, d, *J* = 2.0 Hz), 8.37 (1H, s), 7.46 (1H, s), 4.88 (1H, d, *J* = 15.5 Hz), 3.72 (1H, d, *J* = 15.5 Hz), 4.58 (1H, brs), 3.99 (3H, s), 3.94 (3H, s), 3.93 (3H, s), 3.53 (1H, brs), 3.36 (1H, brs), 2.67 (1H, m), 2.49 (1H, m), 2.34 (1H, m), 1.88–1.93 (3H, m), 1.74–1.77 (2H, m), 1.54–1.63 (4H, m), 1.27–1.41 (3H, m). HRESIMS calcd for [M+H]^+^ C_28_H_35_N_2_O_3_ 447.2642, found 447.2655. HPLC purity: 99.7% [flow rate 1.0 mL/min, 46% MeCN/H_2_O (0.08 M NH_4_H_2_PO_4_, 0.2% Et_3_N)].

### (13a*S*, 14*R*)-14-cyclohexylamino-3, 6, 7-trimethoxyphenanthro[9, 10-*b*]-indolizidine (28)

82% yield. White needles (from MeOH). mp 163–165°C. [α]20 D +57 (*c* 0.1, CH_2_Cl_2_). 99% *ee* [flow rate 1.0 mL/min, 8% isopropanol/hexane and 0.2% Et_3_N, t_R_ (major) = 7.07 min, t_R_ (minor) = 5.49 min]. ^1^H-NMR (500 MHz, C_5_D_5_N): *δ* 8.71 (1H, overlapped), 7.49 (1H, brd, *J* = 9.0 Hz), 8.33 (1H, brs), 8.34 (1H, s), 7.46 (1H, s), 4.81 (1H, brs), 4.66 (1H, d, *J* = 14.5 Hz), 3.71 (1H, d, *J* = 14.5 Hz), 3.98 (3H, s), 3.95 (3H, s), 3.91 (3H, s), 3.25 (1H, brs), 2.86 (1H, m), 2.54 (1H, m), 2.31–2.39 (3H, m), 1.88–1.91 (2H, m), 1.75–1.78 (2H, m), 1.50–1.65 (4H, m), 1.17–1.34 (3H, m), 1.08–1.12 (2H, m). ^13^C-NMR (125 MHz, C_5_D_5_N): *δ* 158.03, 150.55, 150.10 (overlapped), 131.86, 131.38, 128.97, 128.84, 126.18, 125.75, 124.47, 115.13, 105.27, 104.87, 104.77, 69.99, 57.24, 56.03, 55.84, 55.42, 54.44, 54.05, 53.63, 35.84, 33.30, 32.91, 26.52, 25.58, 25.30, 22.60. HRESIMS calcd for [M+H]^+^ C_29_H_37_N_2_O_3_ 461.2799, found 461.2807. HPLC purity: 98.0% [flow rate 1.0 mL/min, 46% MeCN/H_2_O (0.08 M NH_4_H_2_PO_4_, 0.2% Et_3_N)].

### (13a*S*, 14*S*)-14-cyclohexylamino-3, 6, 7-trimethoxyphenanthro[9, 10-*b*]-indolizidine (29)

79% yield. Light yellow solid (from acetone/H_2_O (1∶1)). mp 106–108°C. [α]20 D +87 (*c* 0.1, CH_2_Cl_2_). 100% *ee* [flow rate 1.0 mL/min, 8% isopropanol/hexane and 0.2% Et_3_N, t_R_ = 15.52 min]. ^1^H-NMR (500 MHz, C_5_D_5_N): *δ* 8.59 (1H, d, *J* = 9.0 Hz), 7.54 (1H, overlapped), 8.37 (1H, brs), 8.35 (1H, s), 7.46 (1H, s), 4.90 (1H, d, *J* = 15.0 Hz), 3.72 (1H, d, *J* = 15.0 Hz), 4.67 (1H, brs), 3.98 (3H, s), 3.94 (3H, s), 3.93 (3H, s), 3.38 (1H, brt, *J* = 8.0 Hz), 3.00 (1H, m), 2.67 (1H, m), 2.49 (1H, m), 2.35 (1H, m), 1.73–2.00 (6H, m), 1.63 (1H, m), 1.53 (1H, m), 1.41 (1H, m), 1.13–1.21 (3H, m), 1.04–1.07 (2H, m). ^13^C-NMR (125 MHz, C_5_D_5_N): *δ* 158.30, 150.58, 150.10 (overlapped), 133.39, 131.48, 127.08, 126.34, 126.26, 126.21, 124.56, 115.42, 105.30, 104.91, 104.66, 65.66, 56.03, 55.81, 55.78, 55.47, 54.69, 54.05, 50.29, 35.65, 35.14, 26.48, 26.30, 25.27, 25.18, 22.91. HRESIMS calcd for [M+H]^+^ C_29_H_37_N_2_O_3_ 461.2799, found 461.2811. HPLC purity: 99.5% [flow rate 1.0 mL/min, 46% MeCN/H_2_O (0.08 M NH_4_H_2_PO_4_, 0.2% Et_3_N)].

### (13a*R*, 14*S*)-14-cyclohexylamino-3, 6, 7-trimethoxyphenanthro[9, 10-*b*]-indolizidine (30)

78% yield. White needles (from MeOH). mp 163–165°C. [α]20 D −55 (*c* 0.1, CH_2_Cl_2_). 99% *ee* [flow rate 1.0 mL/min, 8% isopropanol/hexane and 0.2% Et_3_N, t_R_ (major) = 5.43 min, t_R_ (minor) = 7.16 min]. ^1^H-NMR (500 MHz, C_5_D_5_N): *δ* 8.71 (1H, overlapped), 7.49 (1H, brd, *J* = 9.0 Hz), 8.34 (1H, brs), 8.33 (1H, s), 7.46 (1H, s), 4.81 (1H, d, *J* = 4.0 Hz), 4.66 (1H, d, *J* = 14.5 Hz), 3.71 (1H, d, *J* = 14.5 Hz), 3.98 (3H, s), 3.95 (3H, s), 3.91 (3H, s), 3.25 (1H, brs), 2.85 (1H, m), 2.54 (1H, m), 2.31–2.39 (3H, m), 1.1.89–1.90 (2H, m), 1.76–1.81 (2H, m), 1.50–1.65 (4H, m), 1.17–1.31 (3H, m), 1.08–1.12 (2H, m). HRESIMS calcd for [M+H]^+^ C_29_H_37_N_2_O_3_ 461.2799, found 461.2814. HPLC purity: 98.1% [flow rate 1.0 mL/min, 46% MeCN/H_2_O (0.08 M NH_4_H_2_PO_4_, 0.2% Et_3_N)].

### (13a*R*, 14*R*)-14-cyclohexylamino-3, 6, 7-trimethoxyphenanthro[9, 10-*b*]-indolizidine (31)

76% yield. Light yellow solid (from acetone/H_2_O (1∶1)). mp 106–108°C. [α]20 D −86 (*c* 0.1, CH_2_Cl_2_). 99% *ee* [flow rate 1.0 mL/min, 8% isopropanol/hexane and 0.2% Et_3_N, t_R_ (major) = 30.72 min, t_R_ (minor) = 15.92 min]. ^1^H-NMR (500 MHz, C_5_D_5_N): *δ* 8.60 (1H, d, *J* = 9.0 Hz), 7.55 (1H, overlapped), 8.39 (1H, brs), 8.37 (1H, s), 7.48 (1H, s), 4.91 (1H, d, *J* = 15.0 Hz), 3.73 (1H, d, *J* = 15.0 Hz), 4.69 (1H, brs), 4.00 (3H, s), 3.96 (3H, s), 3.95 (3H, s), 3.40 (1H, brt, *J* = 8.0 Hz), 3.01 (1H, m), 2.69 (1H, m), 2.53 (1H, m), 2.37 (1H, m), 1.75–2.02 (6H, m), 1.65 (1H, m), 1.56 (1H, m), 1.42 (1H, m), 1.17–1.22 (3H, m), 1.05–1.09 (2H, m). HRESIMS calcd for [M+H]^+^ C_29_H_37_N_2_O_3_ 461.2799, found 461.2813. HPLC purity: 99.8% [flow rate 1.0 mL/min, 46% MeCN/H_2_O (0.08 M NH_4_H_2_PO_4_, 0.2% Et_3_N)].

### (13a*S*, 14*R*)-14-amino-3, 6, 7-trimethoxyphenanthro[9, 10-*b*]-indolizidine (32)

To the solution of **12** (0.1 g) in 10 mL of EtOH and 0.5 mL of concentrated hydrochloric acid was added 10% Pd/C (0.1 g). The mixture was stirred under H_2_ at 25 psi for 24 h and then filtered. The catalyst was washed with 10 mL of water. The filtrate was combined and evaporated. The residue was dissolved in 5 mL of water and alkalified to pH 10 with 10% NaOH. The resulting precipitate was filtered and washed with water. Recrystallized from acetone/H_2_O (1∶2) gave the product as light yellow solid (66 mg, 82%). mp 166–168°C (decomposed). [α]20 D +53 (*c* 0.1, CH_2_Cl_2_). 99% *ee* [flow rate 1.0 mL/min, 8% isopropanol/hexane and 0.2% Et_3_N, t_R_ (major) = 20.45 min, t_R_ (minor) = 38.40 min]. ^1^H-NMR (500 MHz, C_5_D_5_N): *δ* 8.70 (1H, overlapped), 7.40 (1H, dd, *J* = 9.5 Hz, 2.0 Hz), 8.34 (2H, brs), 7.44 (1H, s), 4.67 (1H, d, *J* = 15.0 Hz), 3.67 (1H, d, *J* = 15.0 Hz), 4.62 (1H, d, *J* = 7.5 Hz), 3.99 (3H, s), 3.95 (3H, s), 3.90 (3H, s), 3.31 (1H, brt, *J* = 7.5 Hz), 2.29–2.43 (3H, m), 1.98–2.10 (2H, m), 1.87–1.88 (2H, m), 1.77 (1H, m). ^13^C-NMR (125 MHz, C_5_D_5_N): *δ* 157.84, 150.41, 150.10 (overlapped), 133.03, 131.88, 128.63, 127.49, 125.90, 125.28, 124.25, 115.07, 105.09, 104.92, 104.69, 71.35, 55.85, 55.65, 55.37, 55.23, 55.12, 54.66, 30.67, 22.03. HRESIMS calcd for [M+H]^+^ C_23_H_27_N_2_O_3_ 379.2016, found 379.2019. HPLC purity: 99.7% [flow rate 1.0 mL/min, 46% MeCN/H_2_O (0.08 M NH_4_H_2_PO_4_, 0.2% Et_3_N)].

### (13a*S*, 14*S*)-14-amino-3, 6, 7-trimethoxyphenanthro[9, 10-*b*]-indolizidine (33)

**33** was synthesized under the same reaction condition as **32** from **13**. The product was obtained as light yellow solid (from acetone/H_2_O (1∶2)). 80% yield. mp 133–135°C. [α]20 D +154 (*c* 0.1, CH_2_Cl_2_). 100% *ee* [flow rate 1.0 mL/min, 26% isopropanol/hexane and 0.2% Et_3_N, t_R_ = 31.26 min]. ^1^H-NMR (500 MHz, C_5_D_5_N): *δ* 8.53 (1H, d, *J* = 9.0 Hz), 7.45 (1H, brd, *J* = 9.0 Hz), 8.36 (2H, brs), 7.42 (1H, s), 4.76 (1H, d, *J* = 15.0 Hz), 3.68 (1H, d, *J* = 15.0 Hz), 4.47 (1H, brs), 3.99 (3H, s), 3.93 (3H, s), 3.92 (3H, s), 3.32(1H, brt, *J* = 7.5 Hz), 2.58 (1H, m), 2.30–2.36 (2H, m), 2.12–2.16 (2H, m), 1.76–1.86 (3H, m). ^13^C-NMR (125 MHz, C_5_D_5_N): *δ* 158.29, 150.54, 150.10 (overlapped), 133.54, 131.72, 126.93, 125.97, 125.80, 125.71, 124.54, 115.95, 105.28, 105.00, 104.66, 65.11, 56.00, 55.79, 55.74, 55.47, 54.80, 48.41, 25.17, 22.92. HRESIMS calcd for [M+H]^+^ C_23_H_27_N_2_O_3_ 379.2016, found 379.2022. HPLC purity: 99.7% [flow rate 1.0 mL/min, 46% MeCN/H_2_O (0.08 M NH_4_H_2_PO_4_, 0.2% Et_3_N)].

### (13a*R*, 14*S*)-14-amino-3, 6, 7-trimethoxyphenanthro[9, 10-*b*]-indolizidine (34)

83% yield. Light yellow solid (from acetone/H_2_O (1∶2)). mp 166–168°C (decomposed). [α]20 D −53 (*c* 0.1, CH_2_Cl_2_). 99% *ee* [flow rate 1.0 mL/min, 8% isopropanol/hexane and 0.2% Et_3_N, t_R_ (major) = 38.36 min, t_R_ (minor) = 21.01 min]. ^1^H-NMR (500 MHz, C_5_D_5_N): *δ* 8.70 (1H, overlapped), 7.40 (1H, brd, *J* = 9.0 Hz), 8.34 (2H, brs), 7.43 (1H, s), 4.67 (1H, d, *J* = 14.0 Hz), 3.66 (1H, d, *J* = 14.0 Hz), 4.62 (1H, d, *J* = 6.5 Hz), 3.99 (3H, s), 3.95 (3H, s), 3.90 (3H, s), 3.31 (1H, brs), 2.31–2.41 (3H, m), 1.99–2.05 (2H, m), 1.86–1.90 (2H, m), 1.74 (1H, m). HRESIMS calcd for [M+H]^+^ C_23_H_27_N_2_O_3_ 379.2016, found 379.2024. HPLC purity: 98.8% [flow rate 1.0 mL/min, 46% MeCN/H_2_O (0.08 M NH_4_H_2_PO_4_, 0.2% Et_3_N)].

### (13a*R*, 14*R*)-14-amino-3, 6, 7-trimethoxyphenanthro[9, 10-*b*]-indolizidine (35)

75% yield. Light yellow solid (from acetone/H_2_O (1∶2)). mp 133–135°C. [α]20 D −156 (*c* 0.1, CH_2_Cl_2_). 100% *ee* [flow rate 1.0 mL/min, 26% isopropanol/hexane and 0.2% Et_3_N, t_R_ = 20.13 min]. ^1^H-NMR (500 MHz, C_5_D_5_N): *δ* 8.53 (1H, d, *J* = 9.0 Hz), 7.45 (1H, d, *J* = 9.0 Hz), 8.37 (2H, brs), 7.43 (1H, s), 4.76 (1H, d, *J* = 15.0 Hz), 3.68 (1H, d, *J* = 15.0 Hz), 4.48 (1H, brs), 3.99 (3H, s), 3.93 (3H, s), 3.92 (3H, s), 3.33(1H, brs), 2.59 (1H, m), 2.33–2.35 (2H, m), 2.13–2.20 (2H, m), 1.77–1.84 (3H, m). HRESIMS calcd for [M+H]^+^ C_23_H_27_N_2_O_3_ 379.2016, found 379.2019. HPLC purity: 99.7% [flow rate 1.0 mL/min, 46% MeCN/H_2_O (0.08 M NH_4_H_2_PO_4_, 0.2% Et_3_N)].

### Cell culture

HCT8, U251, HepG2, A549, A2780, BGC823, and Capan2 were maintained in the RPMI-1640 medium containing 2 g/L sodium bicarbonate, 10% (v/v) fetal bovine serum (FBS; HyClone, Logan, UT) supplemented with 100 units/mL of penicillin and streptomycin (Sigma-Aldrich, St. Louis, MO). All cells were cultured at 37°C in a humidified incubator with 5% CO_2_. Test compounds were dissolved in DMSO (Sigma-Aldrich) to prepare 5 mM stock for the following experiments. The stock was diluted with culture medium to desired concentrations for drug treatment.

### MTT cytotoxicity assay

Doxorubicin (Sigma-Aldrich) was used as the positive control cytotoxic drug in this experiment. The cytotoxicity of deoxytylophorinine and its derivatives on cells were assessed using MTT method. HCT8, U251, HepG2, A549, A2780, BGC823 and Capan2 cells were seeded on 96-well polystyrene cell culture plates at a density of 2×10^4^ cells/mL (100 µL). After 24 h attachment, the cells were treated with six different concentrations of test compounds for 72 h. After that, the drug containing medium was removed and replaced by the culture medium with 100 µL of 0.5 mg/mL MTT (Sigma-Aldrich) solution for 4 h. After that, formazan formed from MTT was extracted by adding 180 µL of DMSO. Absorbance was then determined using a Spectra Max190 (MD, USA) at 570 nm.

### Western blot assay in A549 cells

**1**, **9**, **12**, **16**, **32**, **33**, and **35** were added at a final concentration of 500 nM, and the cell pellets were isolated 24 hours later. And then A549 cells were washed with PBS and were lysed in 100 µL of ice-cold lysis buffer (150 mM NaCl, 50 mM Tris base, 0.5% sodium deoxycholate, 0.5 mM sodium orthovanadate, 1% NP-40, and 0.1% SDS) containing protease inhibitor cocktail and phosphatase inhibitor cocktail. Clear lysates were obtained by centrifugation (12,000 rpm for 20 min). Whole-cell lysates were mixed with 5× sample buffer and heated at 96°C for 10 min. Then equal amounts of protein per lane were separated by SDS-PAGE (4% stacker and 10% resolving). Proteins were then transferred onto nitrocellulose membranes by electroblotting. Nonspecific binding of the membranes was blocked with Tris-buffered saline (TBS) containing 5%(w/v) skim milk and 0.1% (v/v) Tween-20 (TBST) for more than 2 h. And then, the transblotted membranes were incubated with antibodies of Akt (1∶1,000, rabbit monoclonal antibody; Cell Signaling), ERK (1∶500, rabbit polyclonal antibody; Santa Cruz Biotechnology), cyclin A (1∶2,000, mouse monoclonal antibody; Cell Signaling), cyclin B1 (1∶1,000, rabbit monoclonal antibody; oncogene), cyclin D1 (1∶500, mouse monoclonal antibody; Santa Cruz Biotechnology), cyclin E (1∶200, rabbit polyclonal antibody; Beijing Biosynthesis Biotechnology), CDK2 (1∶500, rabbit polyclonal antibody; Santa Cruz Biotechnology), β-actin (1∶500, mouse monoclonal antibody; Santa Cruz Biotechnology) in TBST containing 5% skim milk or phospho-Akt (Ser473) (1∶1,000, rabbit monoclonal antibody; Cell Signaling), phospho-ERK (Tyr204) (1∶500, rabbit polyclonal antibody; Santa Cruz Biotechnology) in TBST containing 3% bovine serum albumin (BSA; Sigma-Aldrich) overnight at 4°C. Subsequently, the membranes were washed with TBST and incubated for 1.5 h with an appropriate secondary antibody (1∶3,000, horseradish peroxidase-conjugated goat anti-mouse or anti-rabbit IgG) at room temperature. After washing the membrane three times for 10 min in TBST, the immunoblots were visualized by enhanced chemiluminescence using ECL Western blotting detection reagents and exposed ECL hyperfilm in Las-3000 (FUJFILM, JP). Multi Gauge v3.0 was used for image acquisition and data analysis.

### Flow cytometric analysis

After 24 h administration of fresh media containing test sample (**1**, **9**, **32**, and **33** at 500 nM), A549 cells were harvested, washed twice with phospho-buffered saline (PBS) and fixed overnight in 70% EtOH at 4°C. After washing twice with cold PBS, the fixed cells were then resuspended in 1 ml of cell cycle buffer (50 µg/mL RNase and 10 µg/mL propidium iodide) for DNA staining at room temperature for 1 h. DNA content was measured on a EPICS XL flow cytometer (Beckman Coulter, USA) and the distribution of cells in the cell cycle was calculated using SYSTEM II software (BD Biosciences, USA).

## Supporting Information

Figure S1 **Apoptosis of A549 cells could be induced by high concentrations of 1.**
(DOC)

Figure S2 **NOE experiments for compounds l and k.** In the NOE experiments, the NOE association between H-14 (*δ*_H_ 5.19, brs) and H-13a (*δ*_H_ 3.96, m) indicated that the H-14 was *cis* to H-13a in compound l. However, for k, the *trans*-orientation of H-14 and H-13a was suggested by the NOE association between H-14 (*δ*_H_ 5.15, d, *J* = 7.0 Hz) and H-13 (*δ*_H_, 2.23, m) and the larger coupling contant of H-14 (*J* = 7.0 Hz).
(DOC)

Figure S3 **NMR spectra for compounds 1–35.**
(DOC)

Figure S4 **Purity data for compounds 1–35.**
(DOC)

Table S1 **Cytotoxic activities of Compounds 1, 9, 12, 16, 32, 33, 35 in A549 cells for 24 h treatment.** These data represent the mean values ± standard deviation of three dependent experiments performed in triplicate.
(DOC)
