# Supplementary figures and images for: Synthesis, Biological Evaluation and Mechanism Studies of Deoxytylophorinine and Its Derivatives as Potential Anticancer Agents
Source: PLoS One. 2012 Jan 19;7(1):e30342. doi: 10.1371/journal.pone.0030342 (PMC3261902; doi:10.1371/journal.pone.0030342)

**Figure S1. Apoptosis of A549 cells could be induced by high concentrations of 1**

**
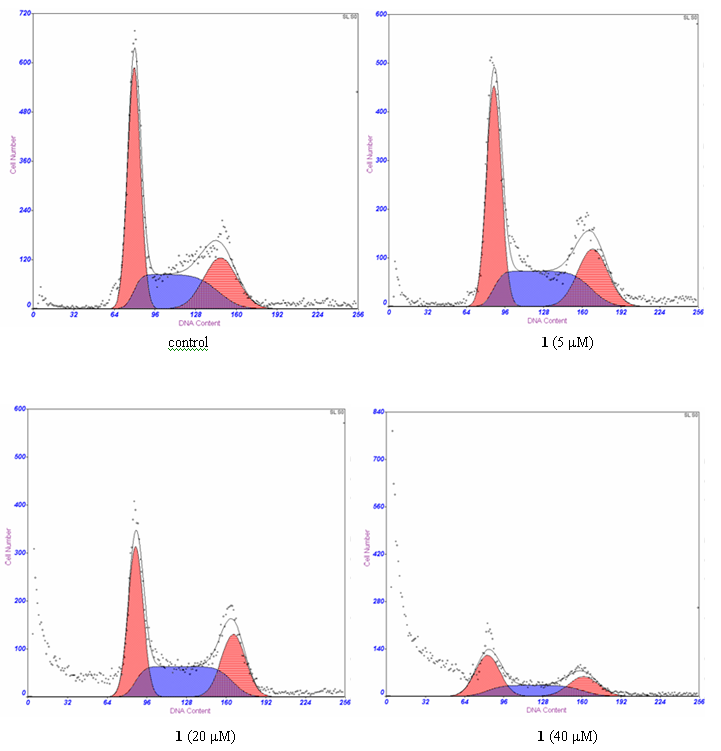
**

|  | SubG0/G1 (%) |
| --- | --- |
| control | 1.95 |
| **1** (5 μM) | 3.58 |
| **1** (20 μM) | 21.0 |
| **1** (40 μM) | 51.2 |

Supplement: Figure S1 — Apoptosis of A549 cells could be induced by high concentrations of 1. (DOC) [file pone.0030342.s001.doc]
