# Supplementary material for: Synthesis, Biological Evaluation and Mechanism Studies of Deoxytylophorinine and Its Derivatives as Potential Anticancer Agents
Source: PLoS One. 2012 Jan 19;7(1):e30342. doi: 10.1371/journal.pone.0030342 (PMC3261902; doi:10.1371/journal.pone.0030342)
Supplement: Figure S2 — NOE experiments for compounds l and k. In the NOE experiments, the NOE association between H-14 (δ H 5.19, brs) and H-13a (δ H 3.96, m) indicated that the H-14 was cis to H-13a in compound l. However, for k, the trans-orientation of H-14 and H-13a was suggested by the NOE association between H-14 (δ H 5.15, d, J = 7.0 Hz) and H-13 (δ H, 2.23, m) and the larger coupling contant of H-14 (J = 7.0 Hz). (DOC) [file pone.0030342.s002.doc]

**Figure S2. NOE experiments for compounds l and k**

In the NOE experiments, the NOE association between H-14 (*δ*_H_ 5.19, brs) and H-13a (*δ*_H_ 3.96, m) indicated that the H-14 was *cis* to H-13a in compound l. However, for k, the *trans*-orientation of H-14 and H-13a was suggested by the NOE association between H-14 (*δ*_H_ 5.15, d, *J* = 7.0 Hz) and H-13 (*δ*_H_, 2.23, m) and the larger coupling contant of H-14 (*J* = 7.0 Hz).

NOE results of **l** and **k**.
