# Supplementary material for: Synthesis, Biological Evaluation and Mechanism Studies of Deoxytylophorinine and Its Derivatives as Potential Anticancer Agents
Source: PLoS One. 2012 Jan 19;7(1):e30342. doi: 10.1371/journal.pone.0030342 (PMC3261902; doi:10.1371/journal.pone.0030342)
Supplement: Figure S3 — NMR spectra for compounds 1–35. (DOC) [file pone.0030342.s003.doc]

**Figure S3. NMR spectra for the compounds 1-35**

**Compound 1**

**
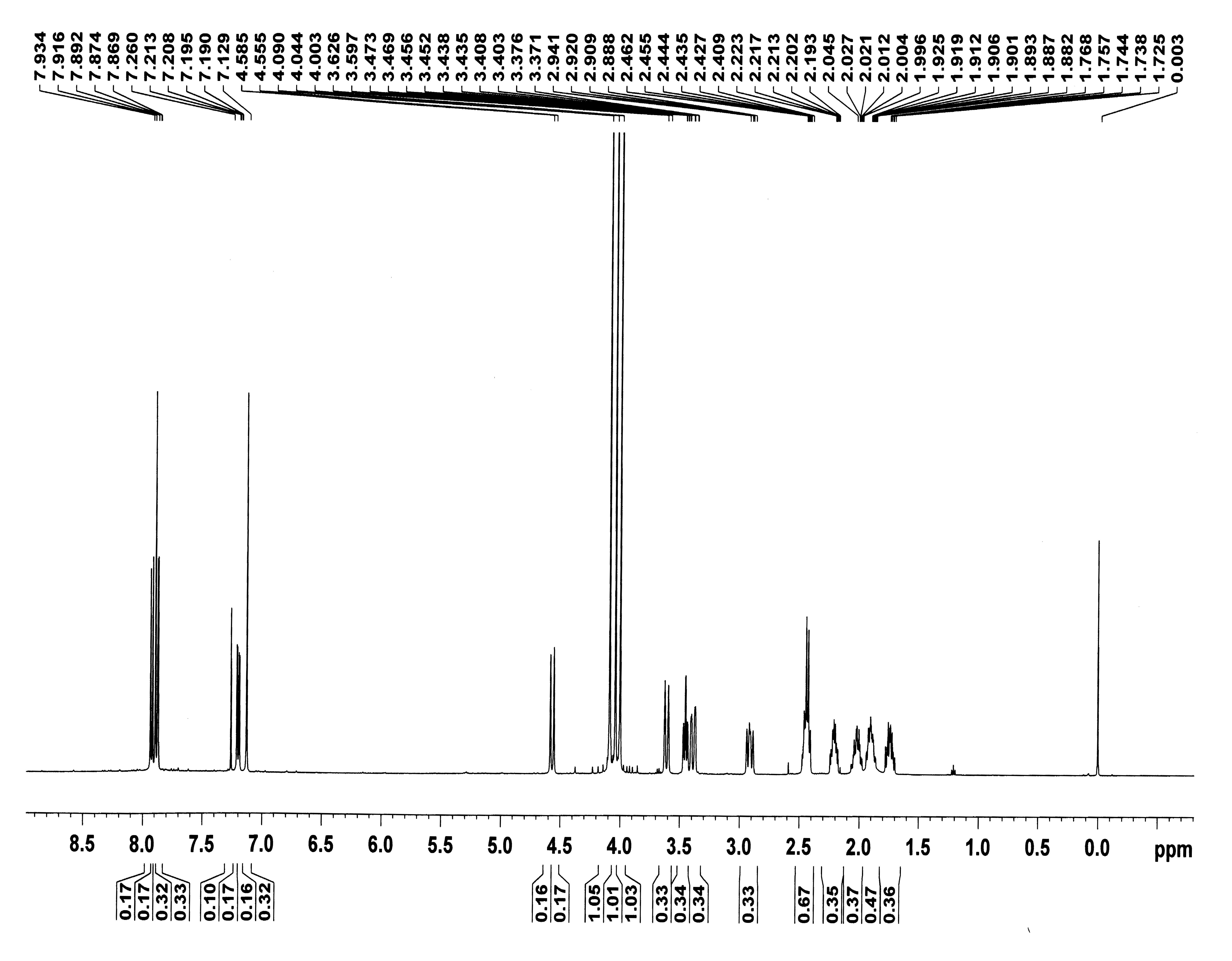

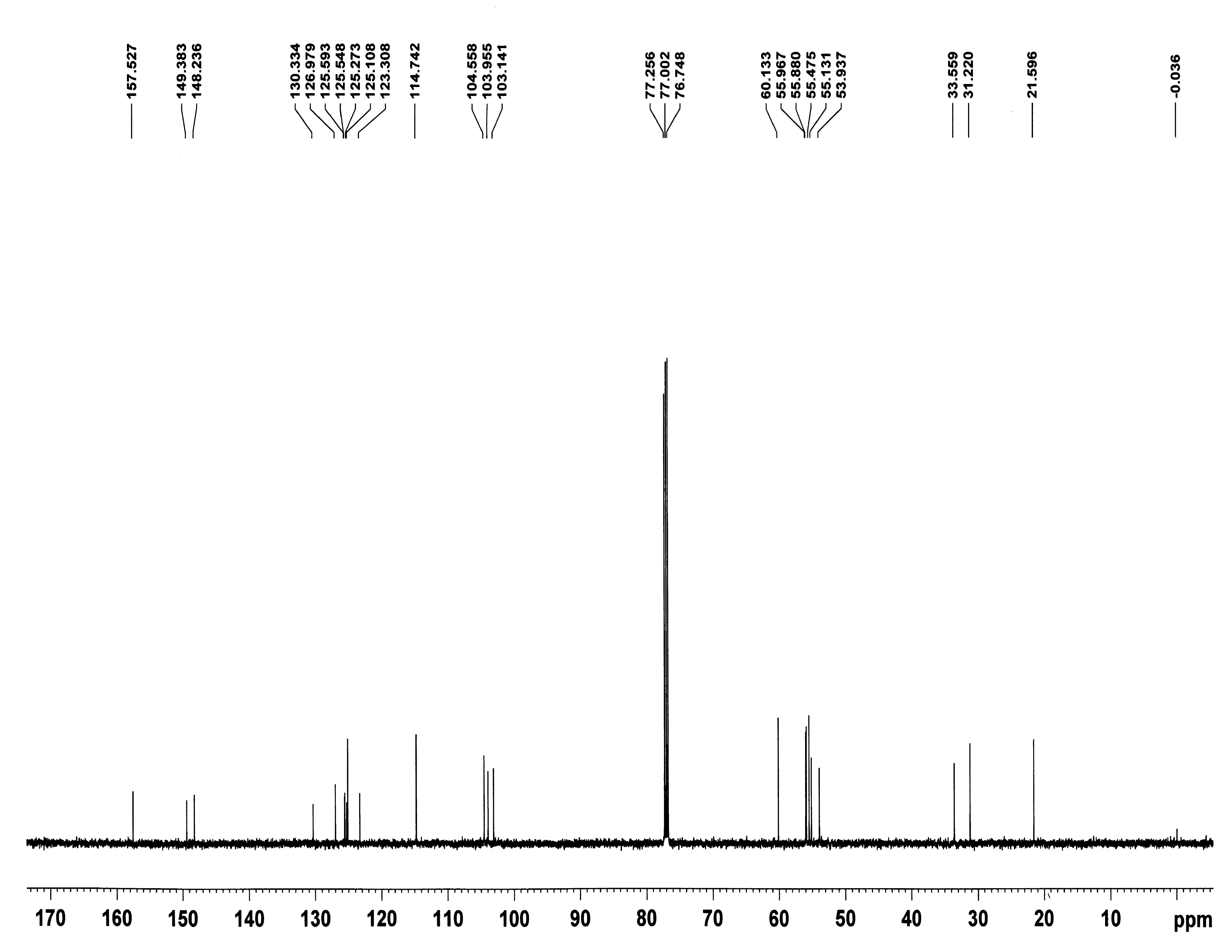
**

**Compound 2**

**
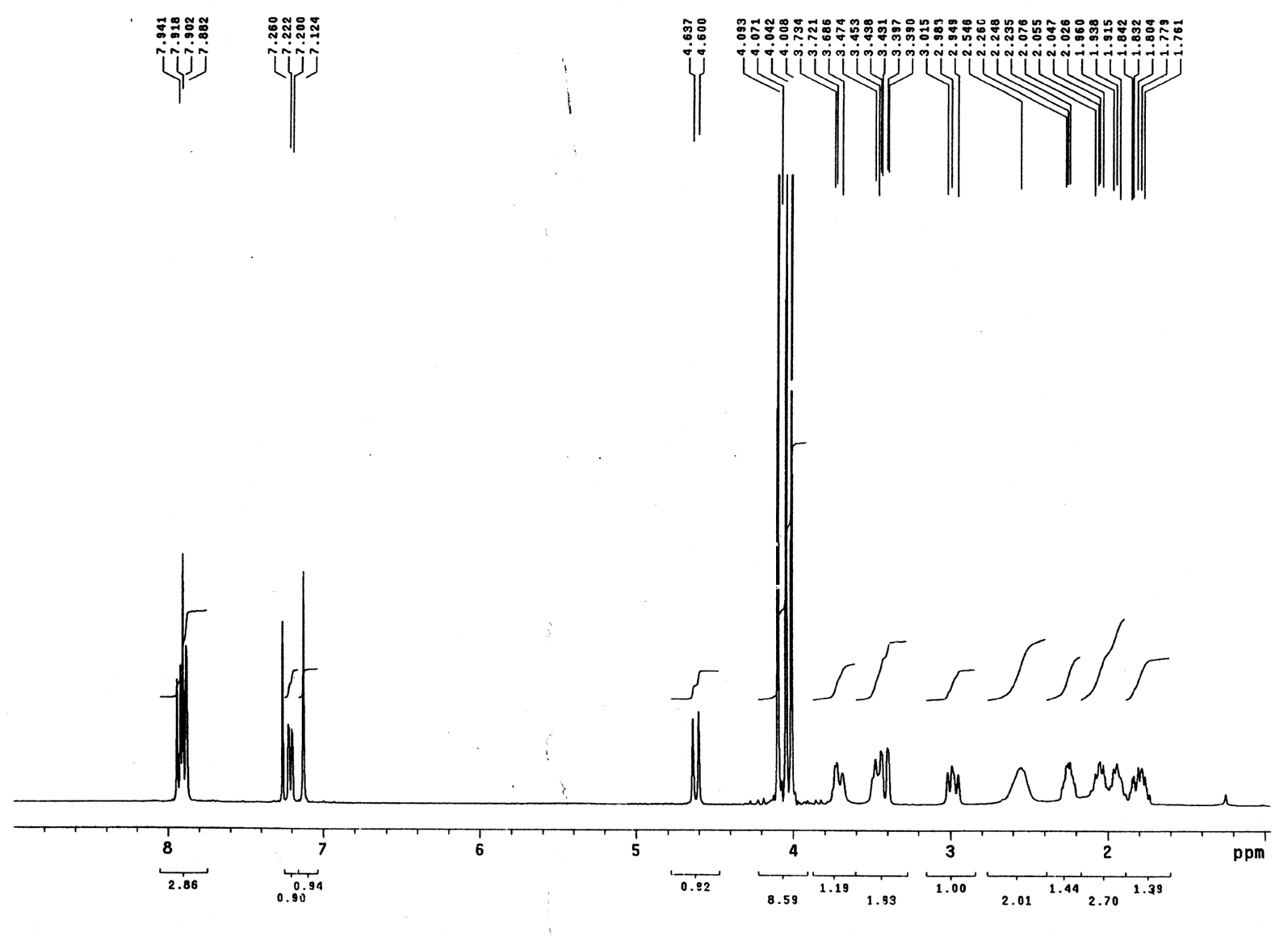
**

**
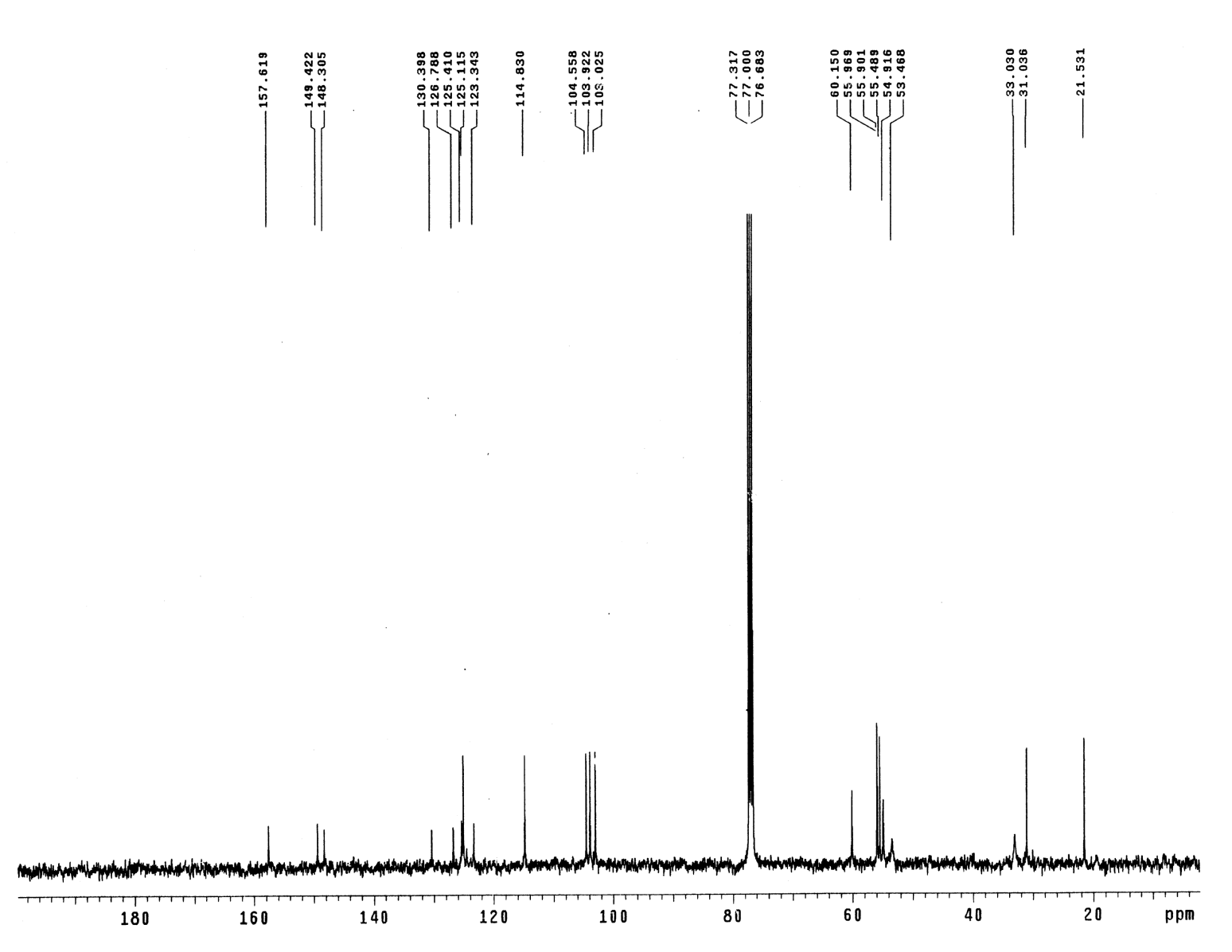
**

**Compound 3**

**
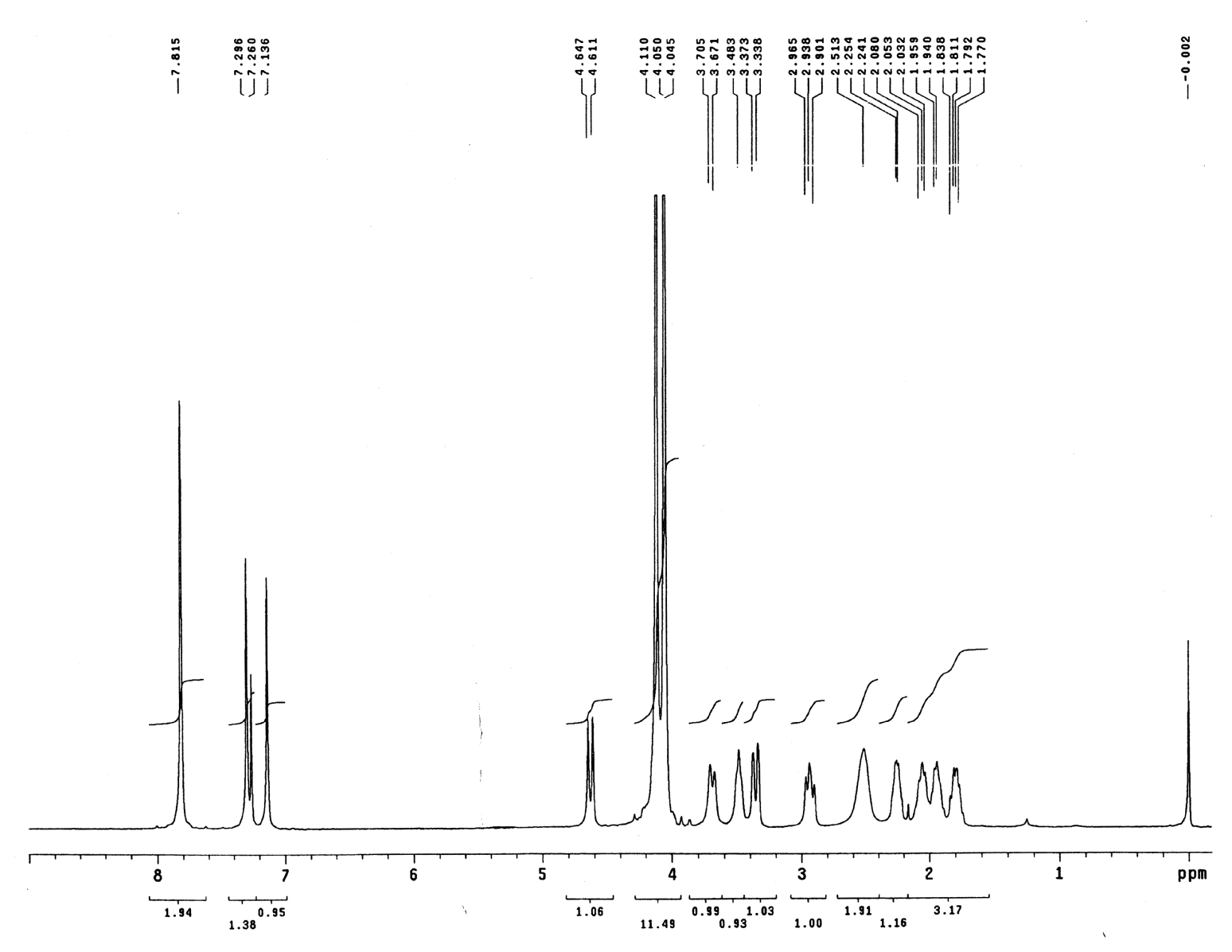
**

**Compound 4**

**
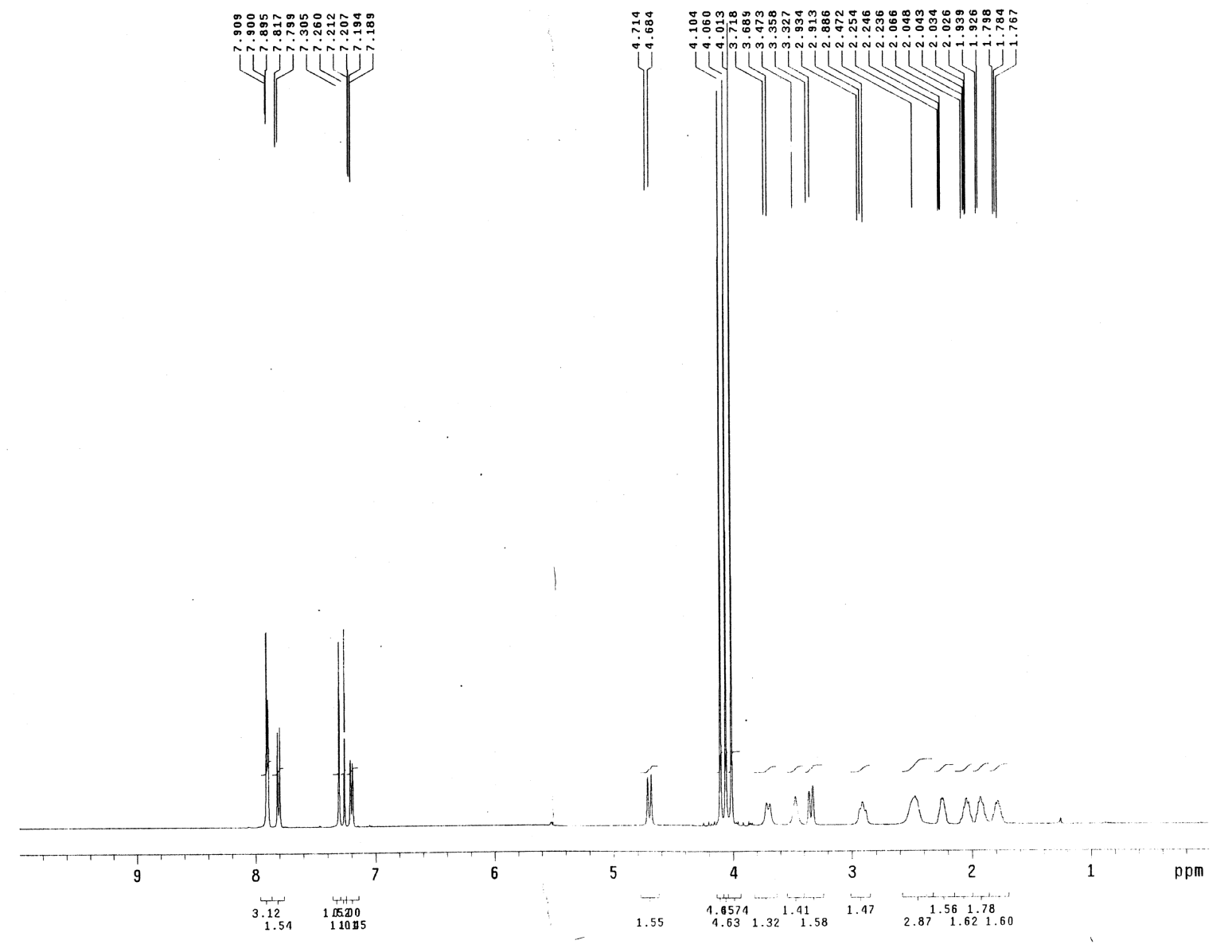
**

**Compound 5**

**
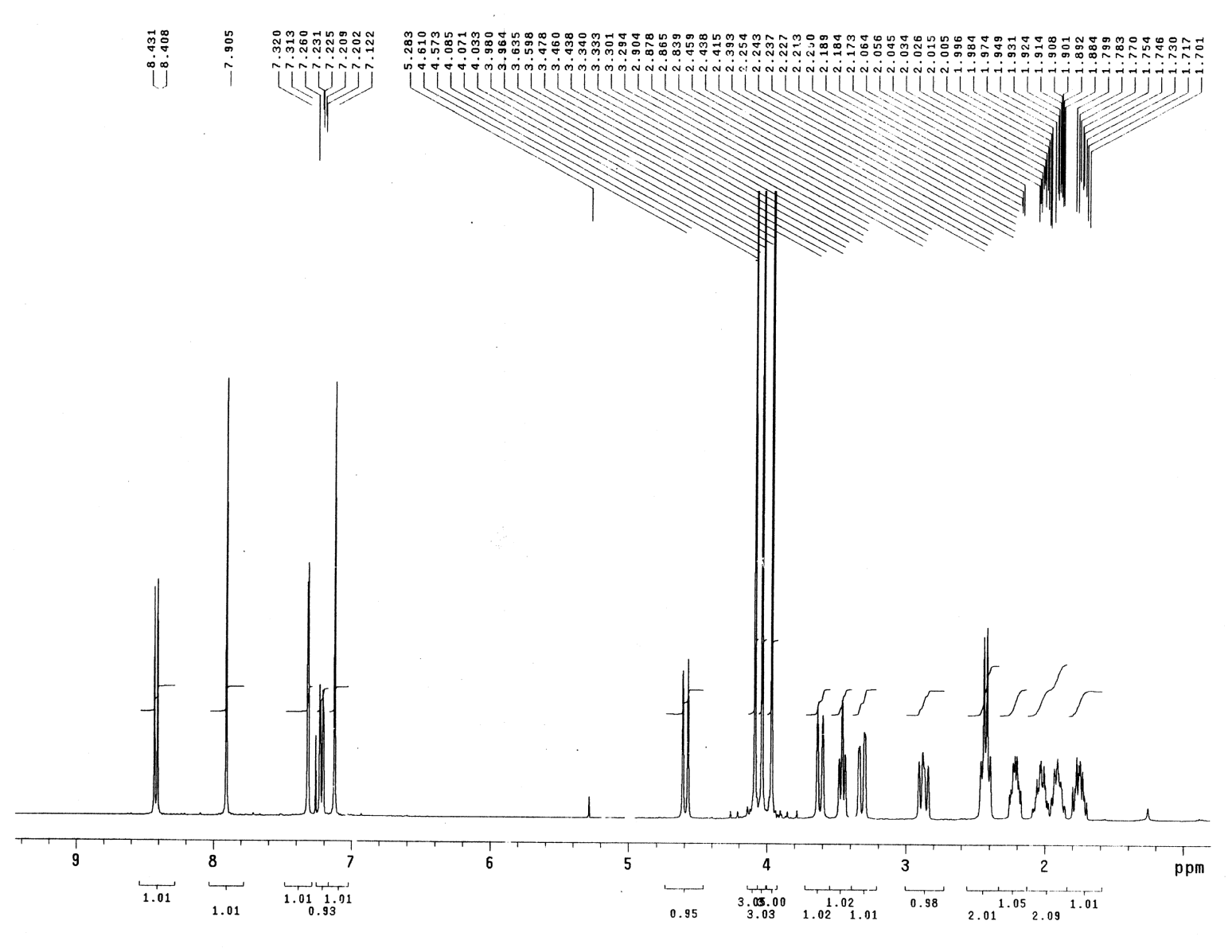
**

**Compound 6**

**
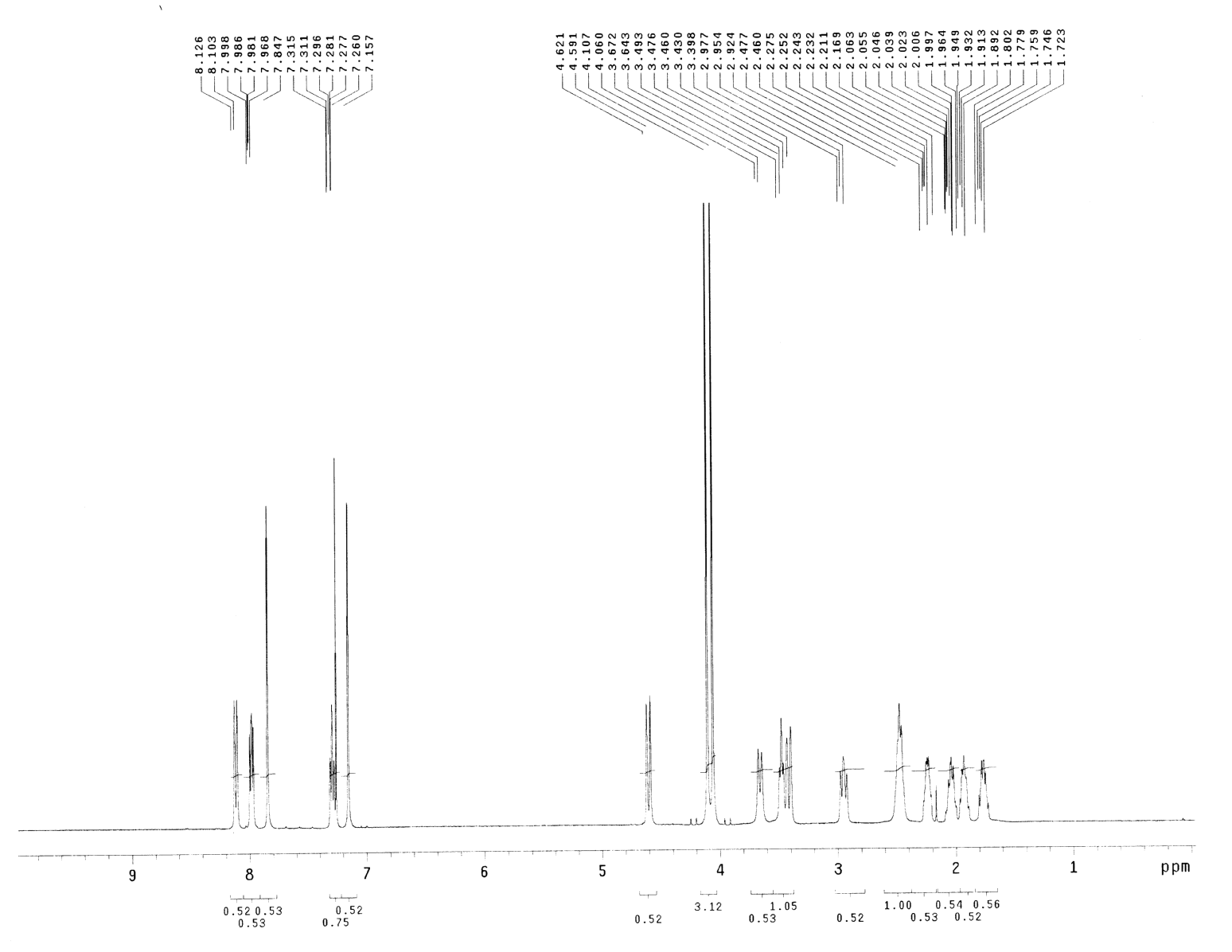
**

**Compound 7**

**
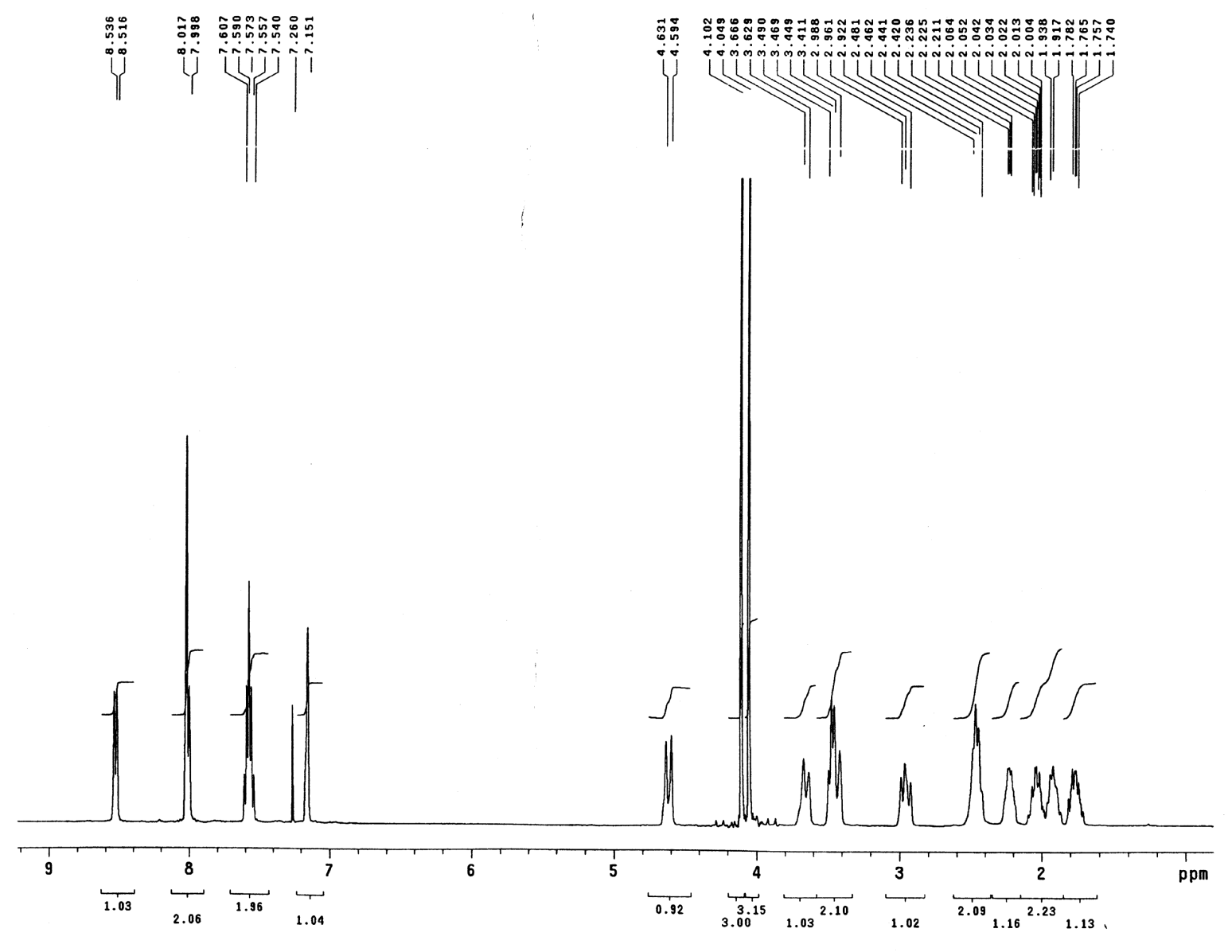
**

**Compound 8**

**
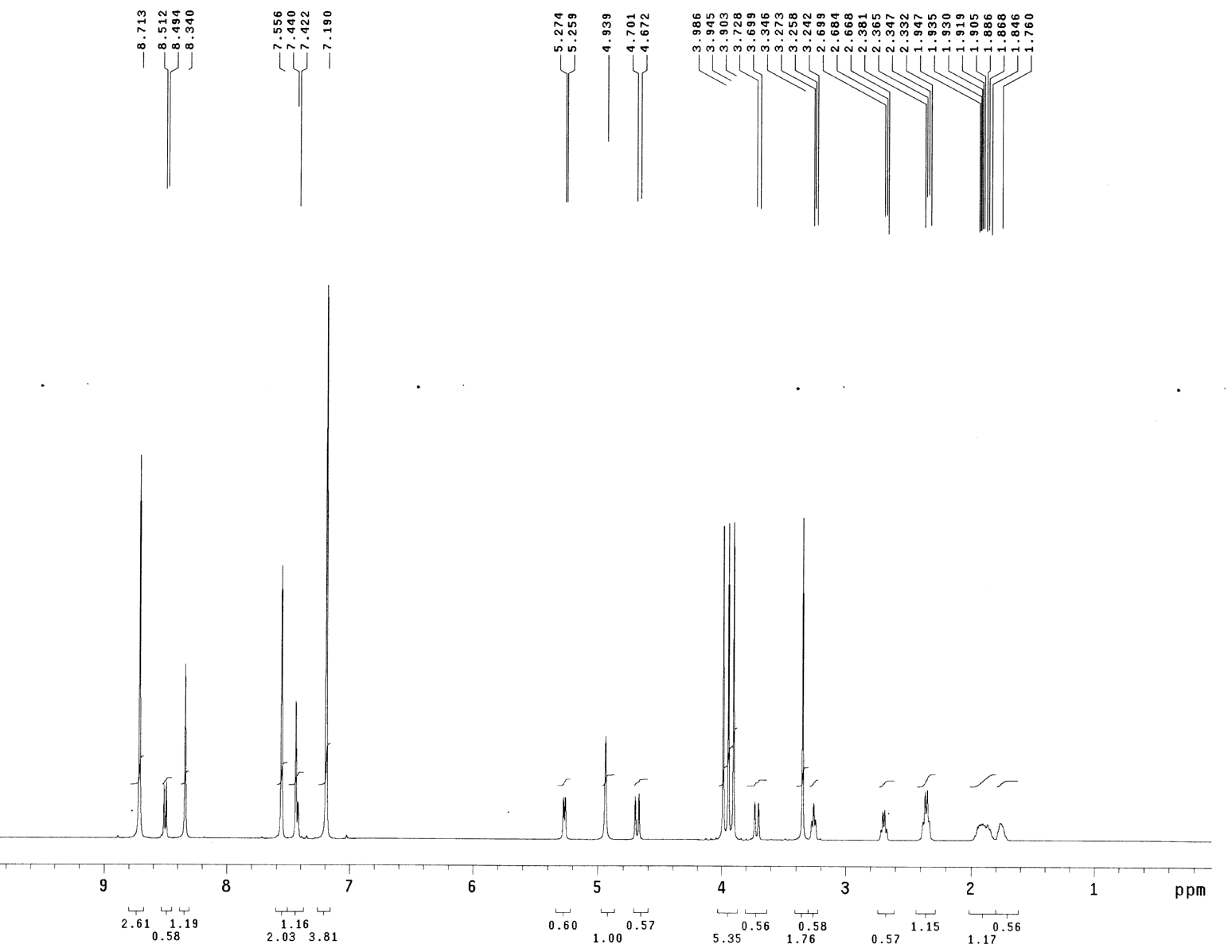
**

**
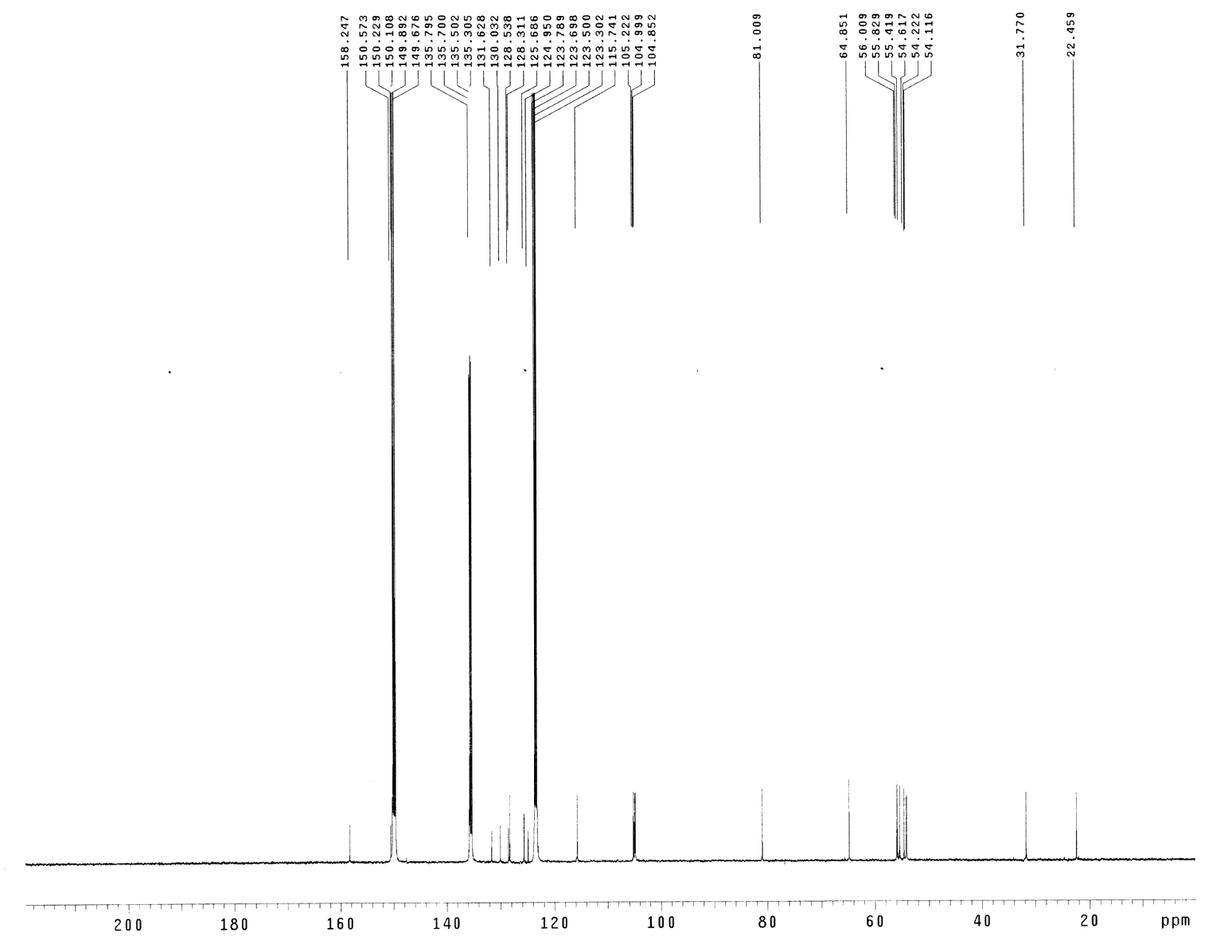
**

**Compound 9**

**
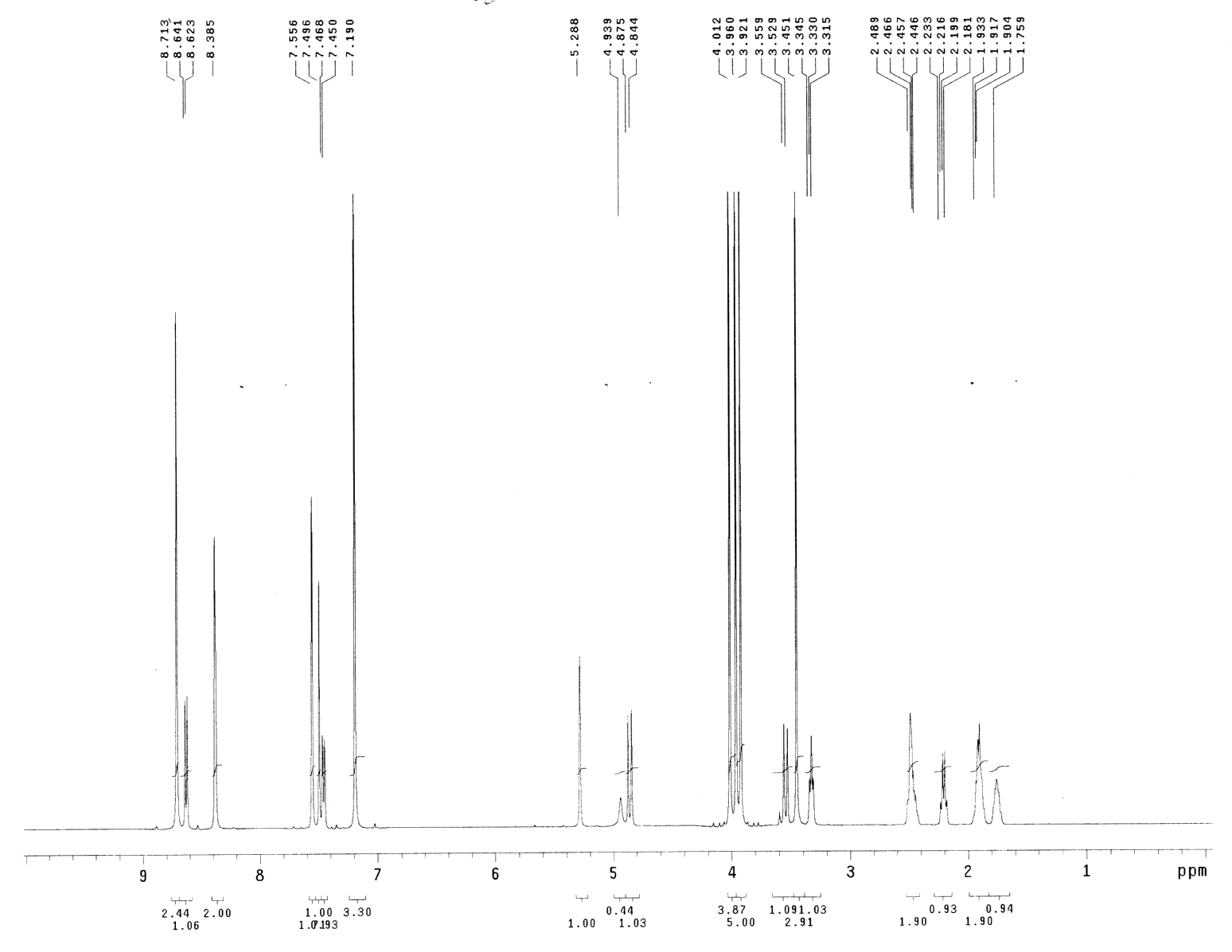
**

**
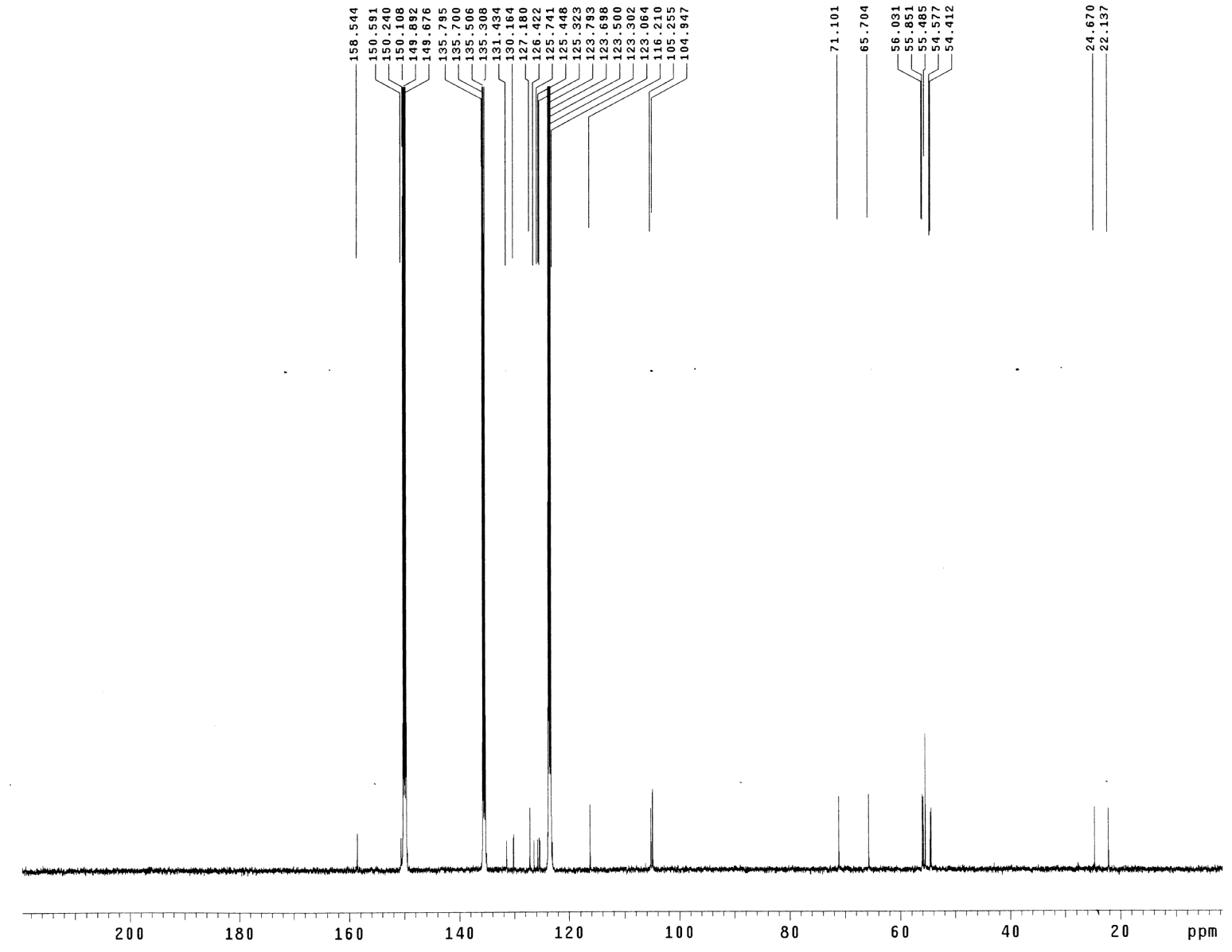
**

**Compound 10**

**
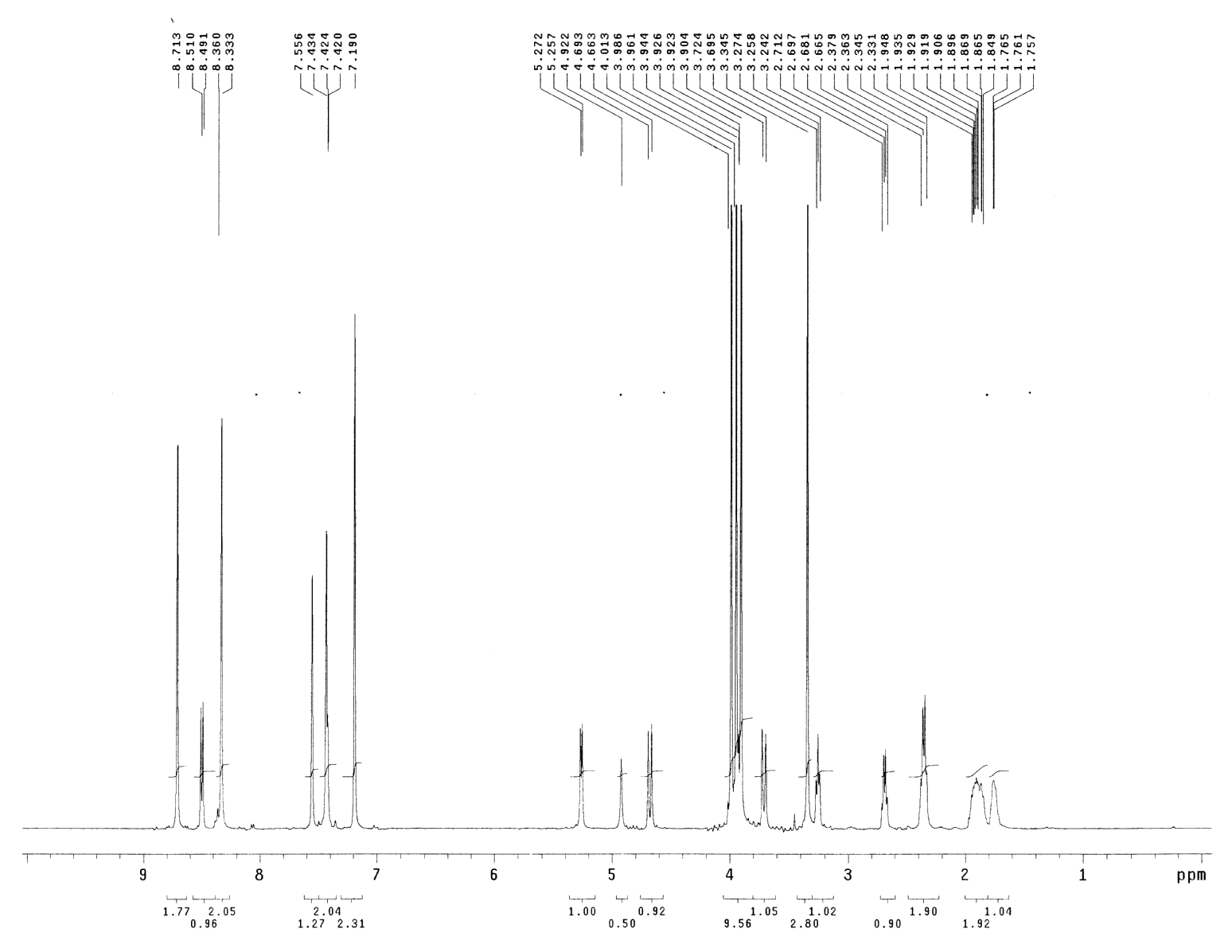
**

**Compound 11**

**
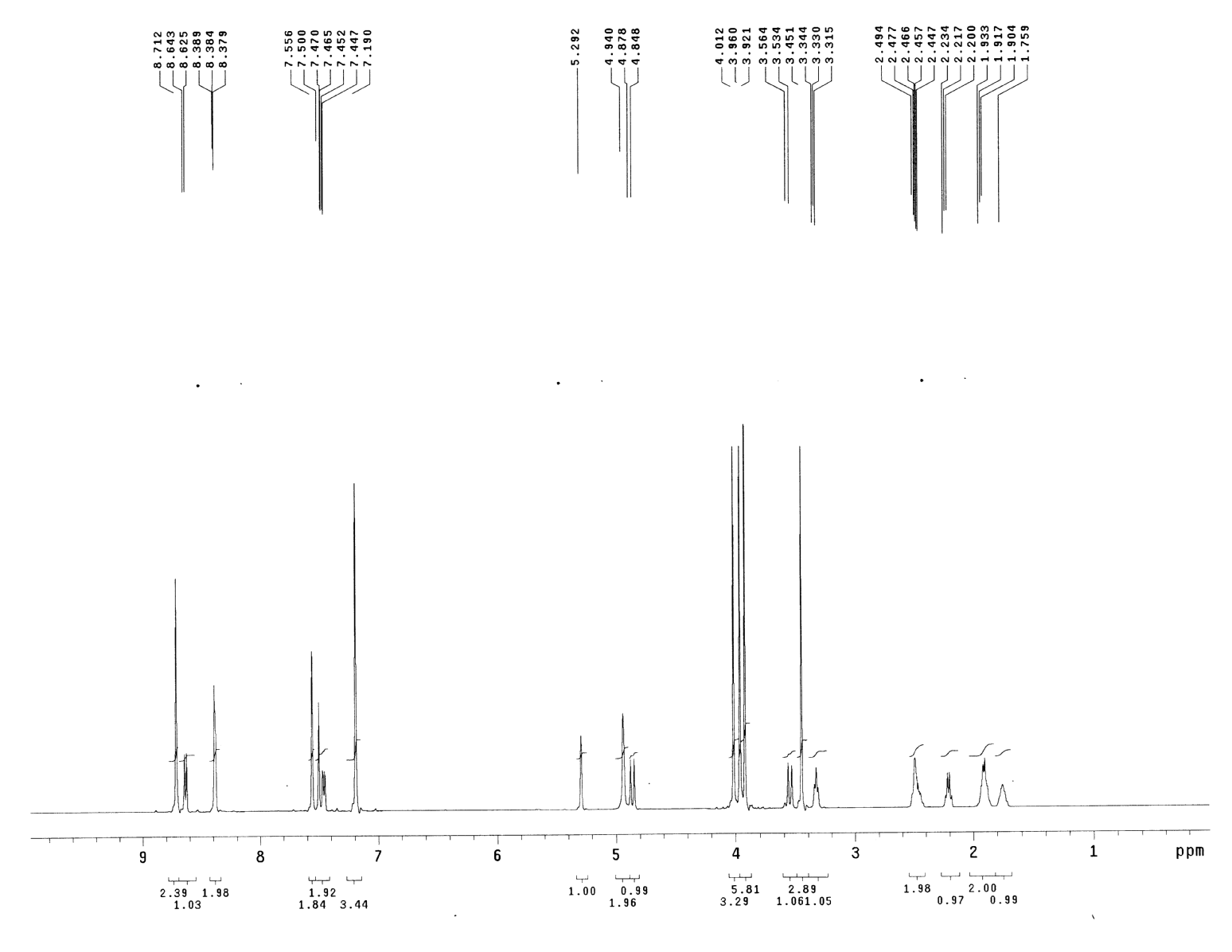
**

**Compound 12**

**
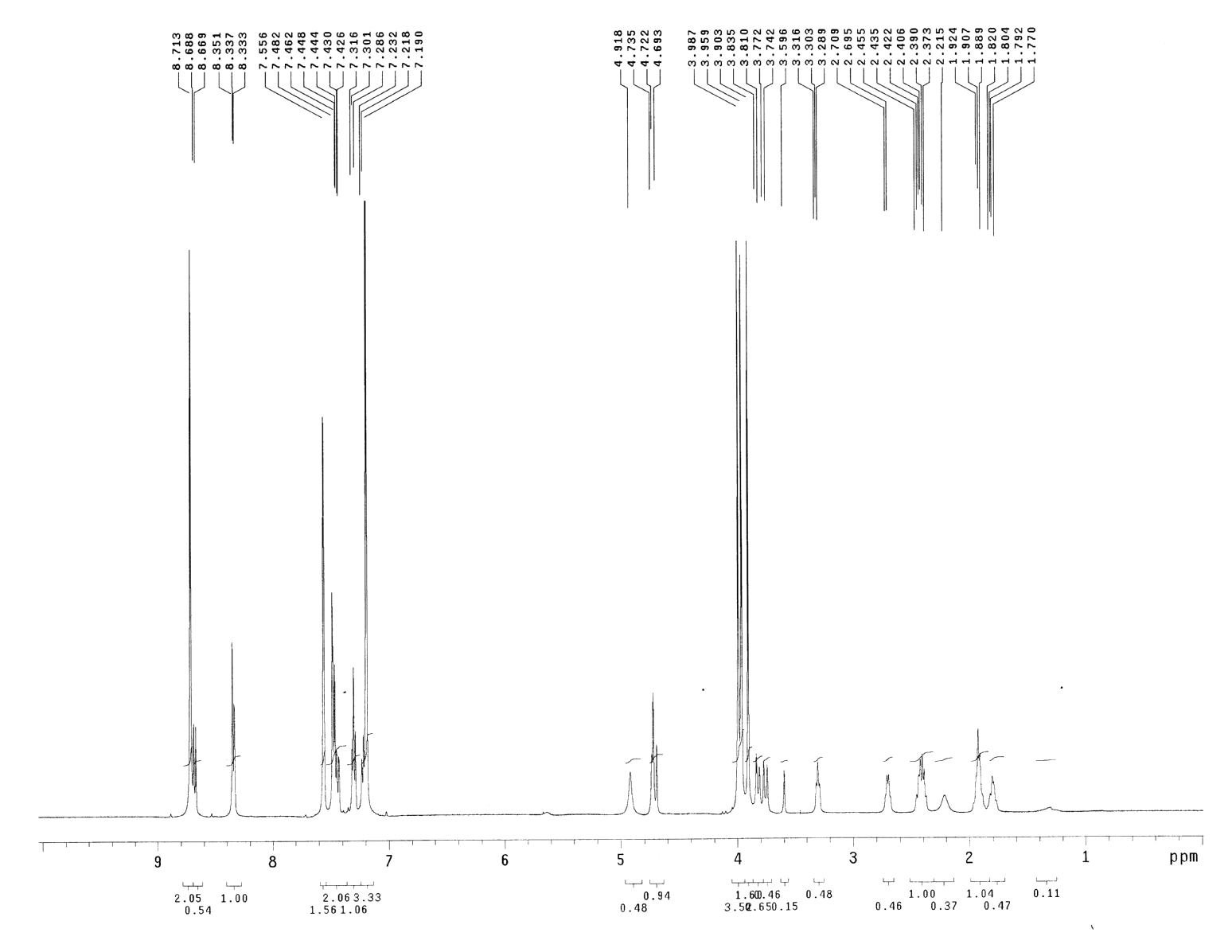
**

**
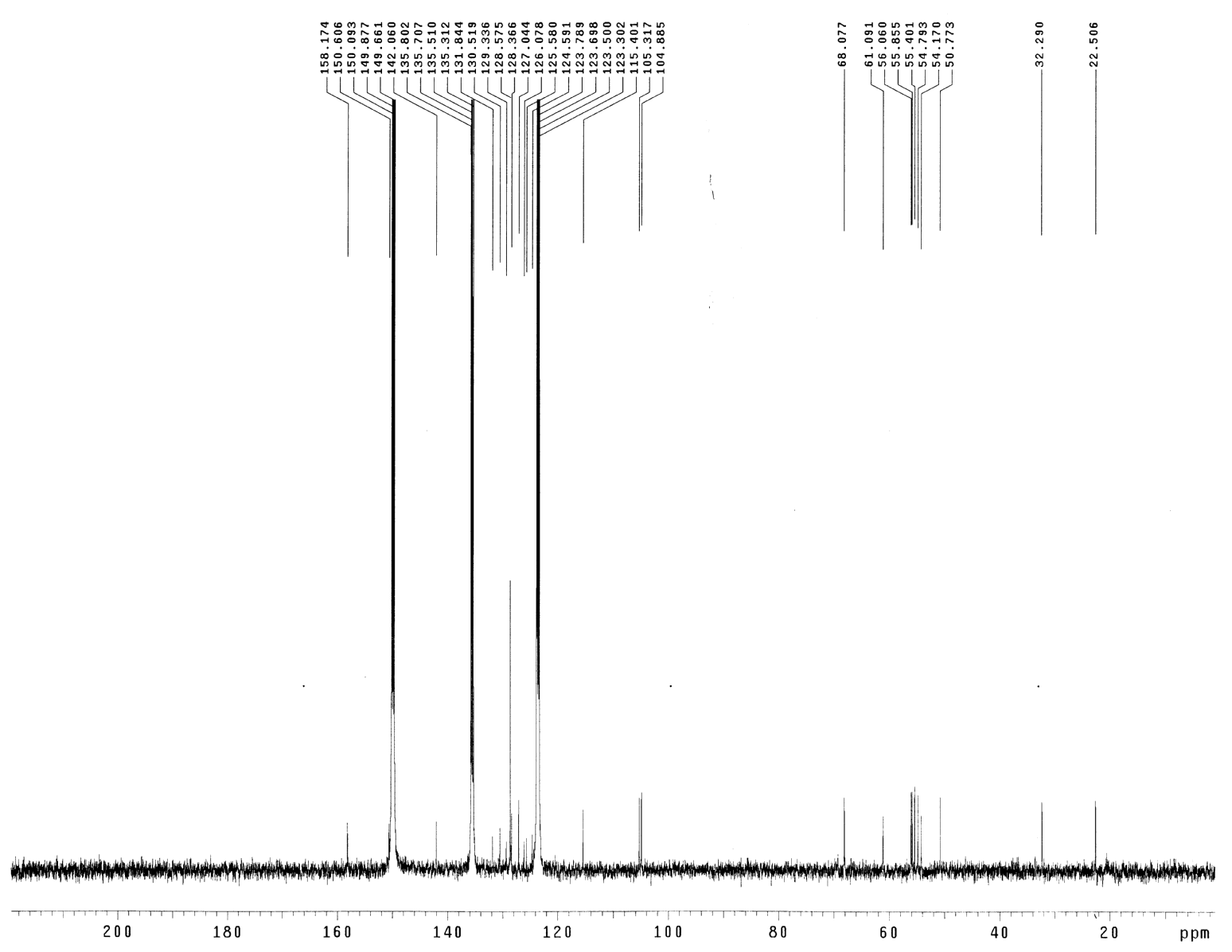
**

**Compound 13**

**
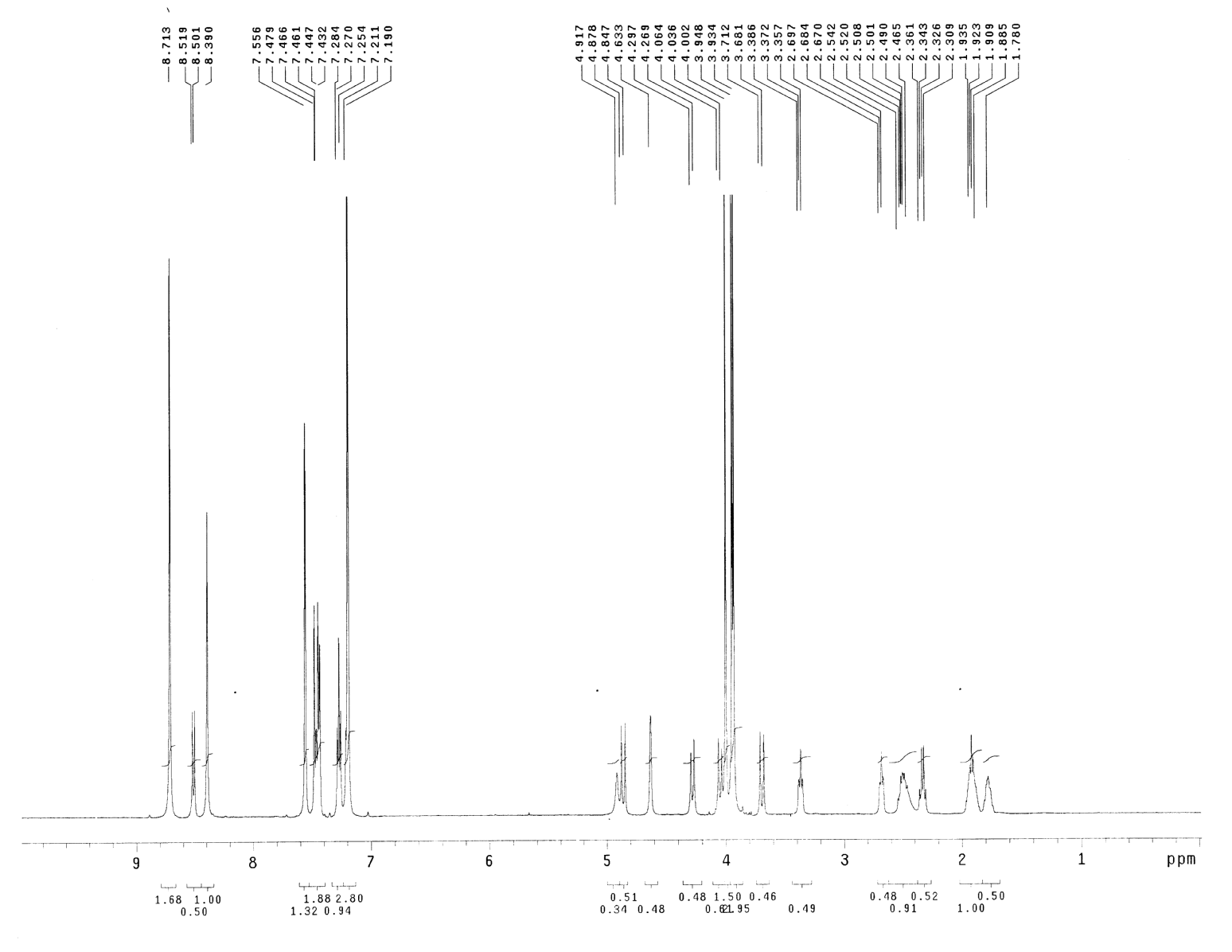
**

**
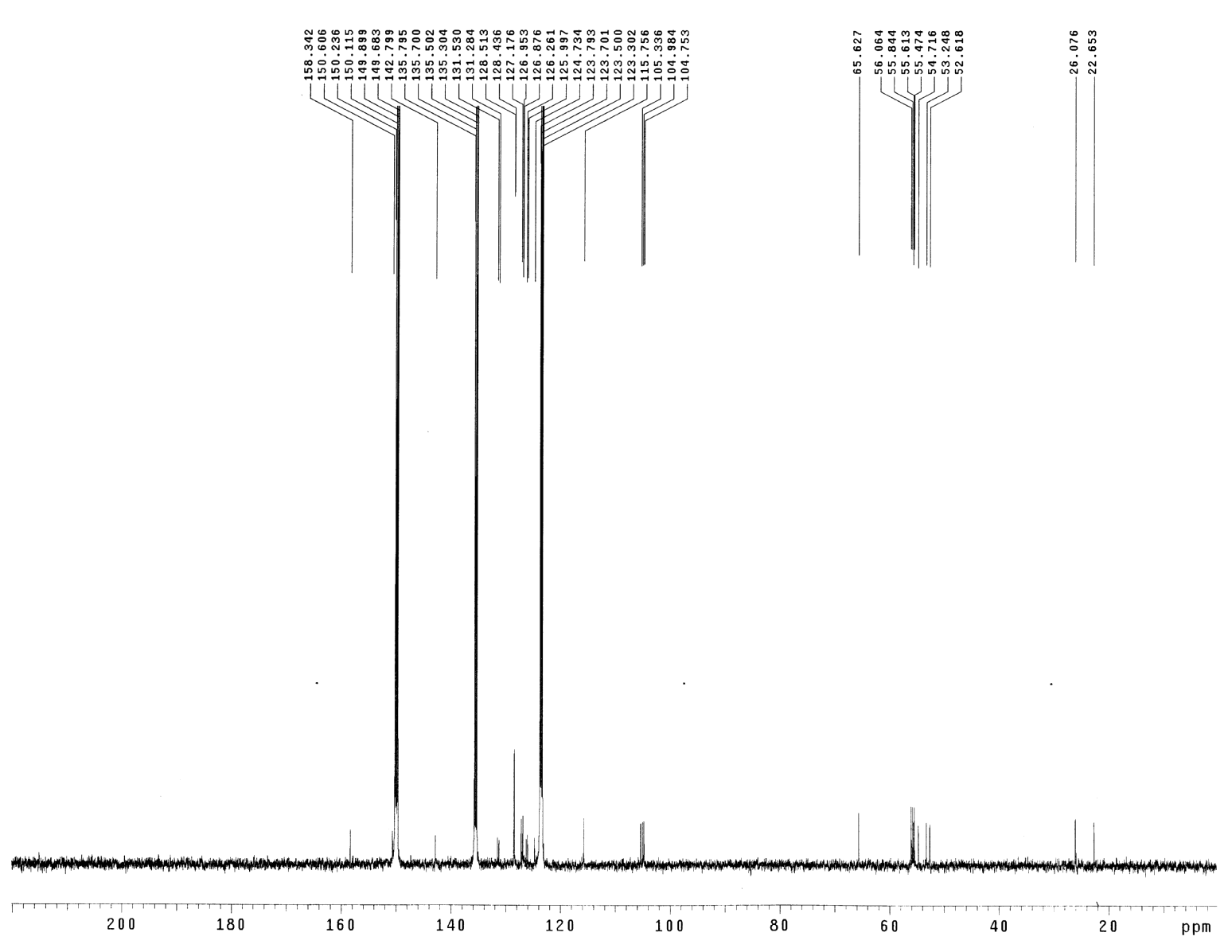
**

**Compound 14**

**
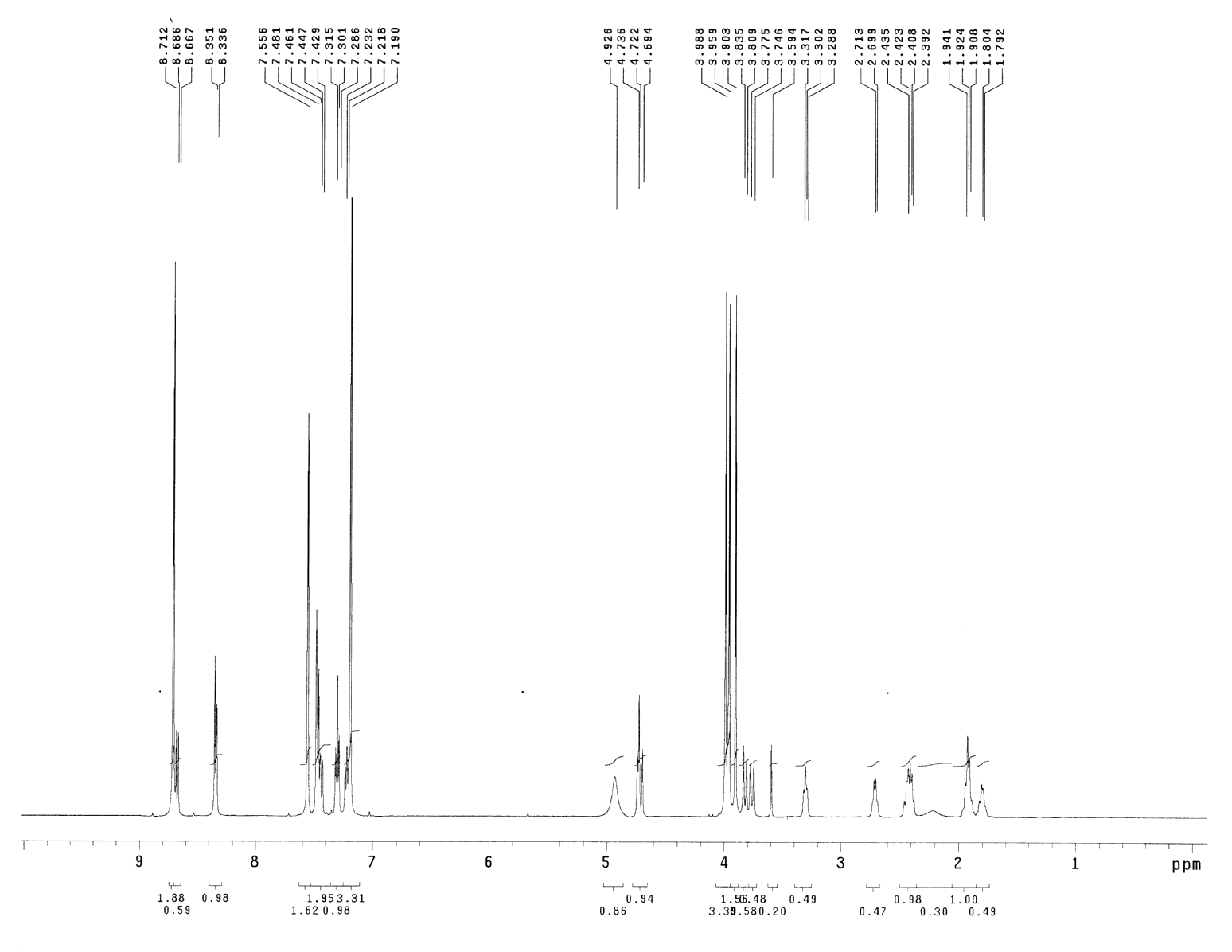
**

**Compound 15**

**
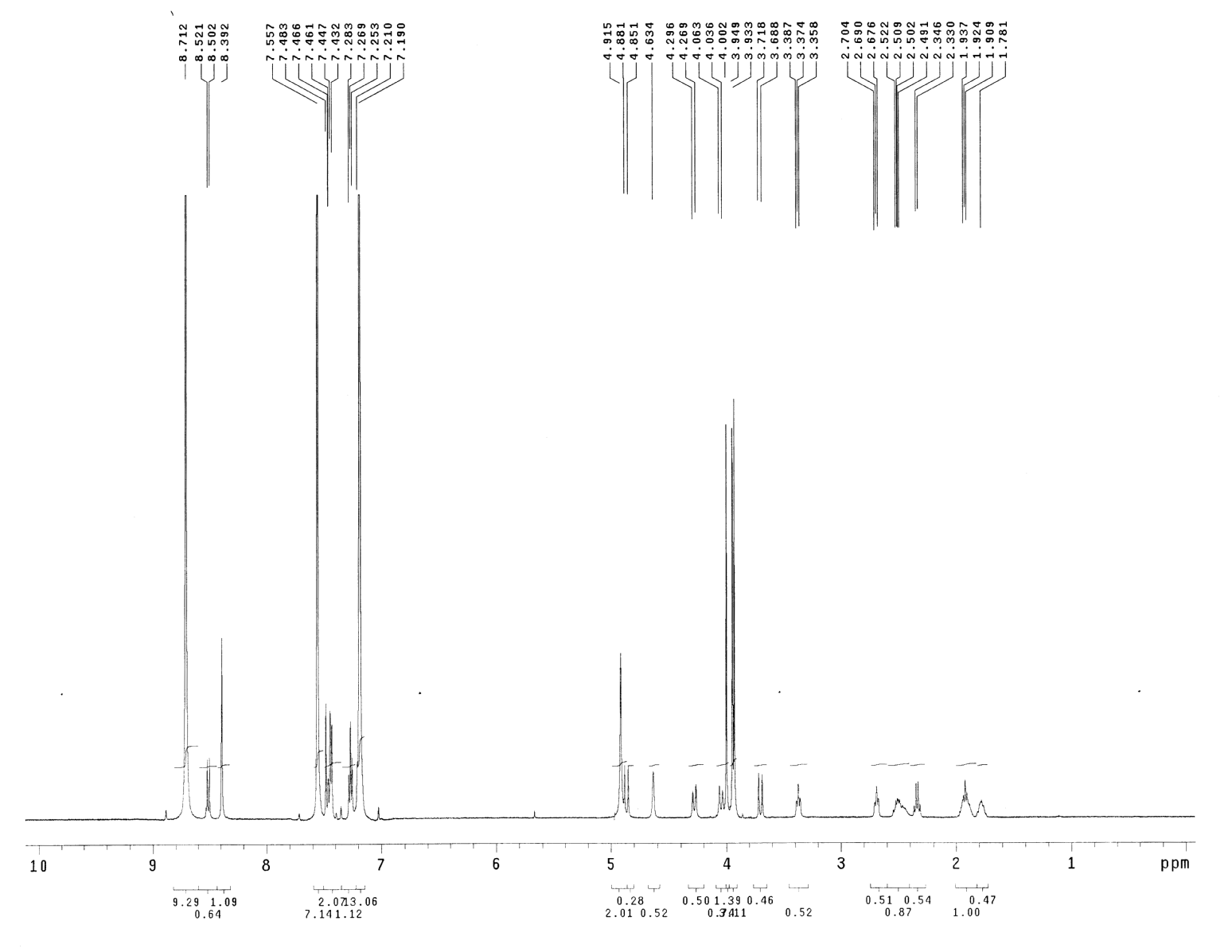
**

**Compound 16**

**
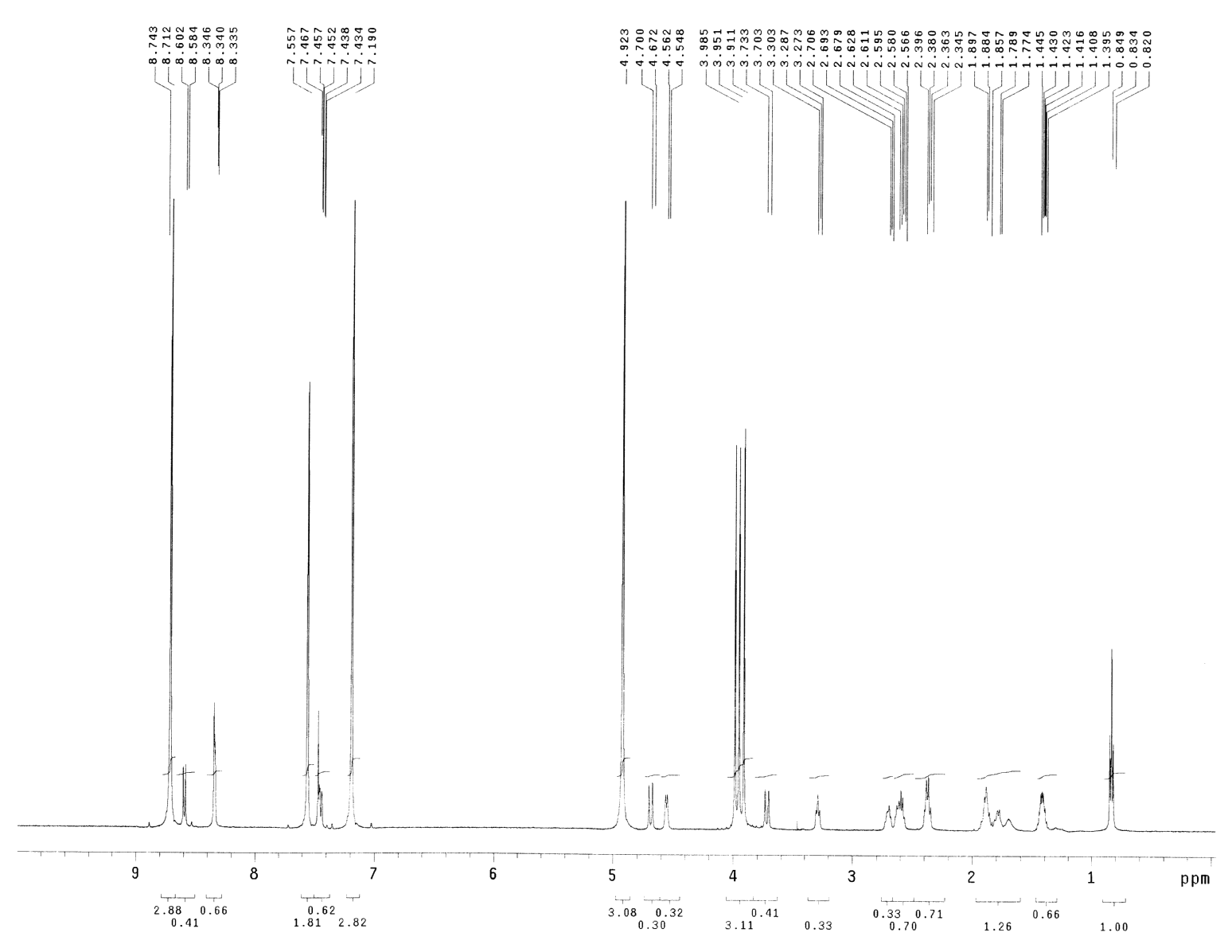
**

**
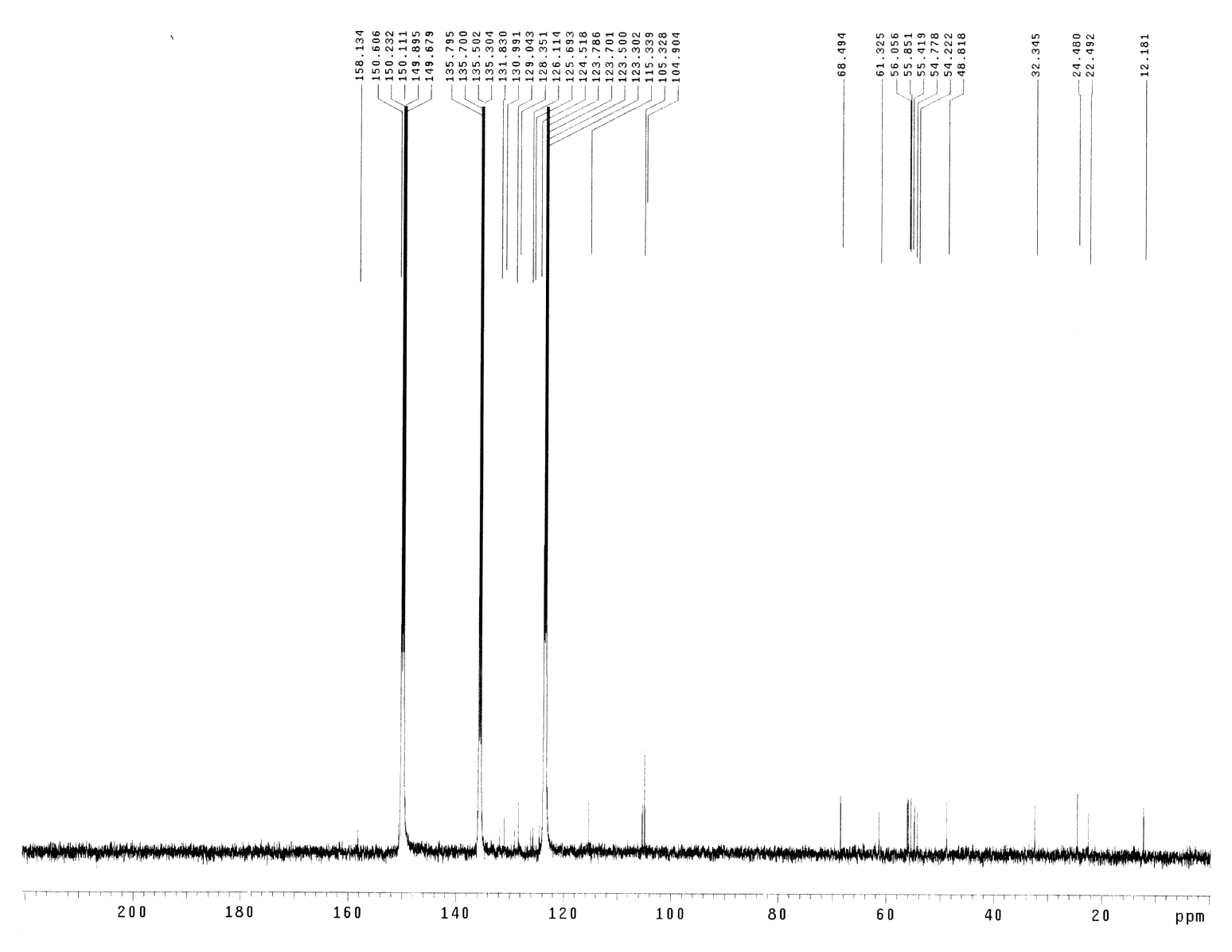
**

**Compound 17**

**
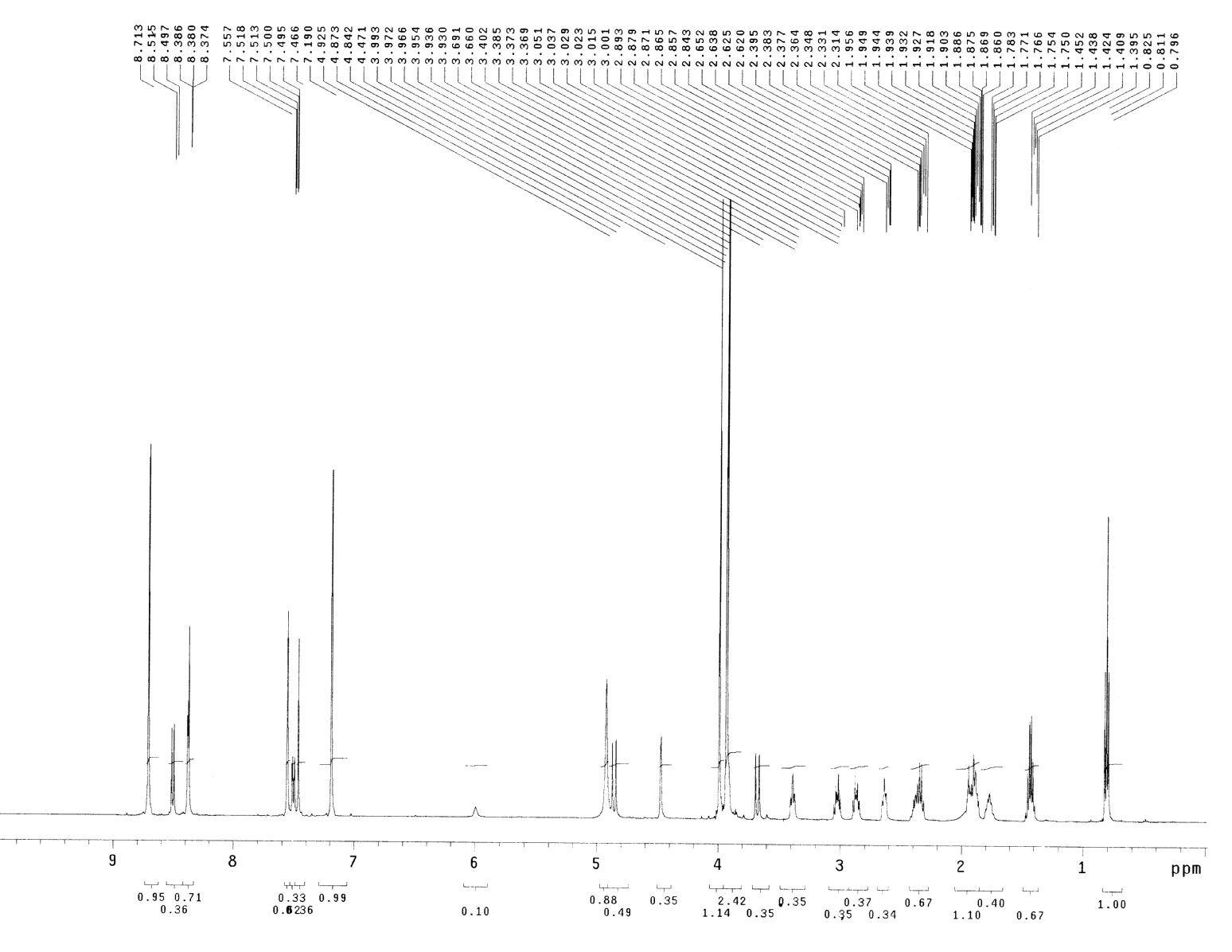
**

**
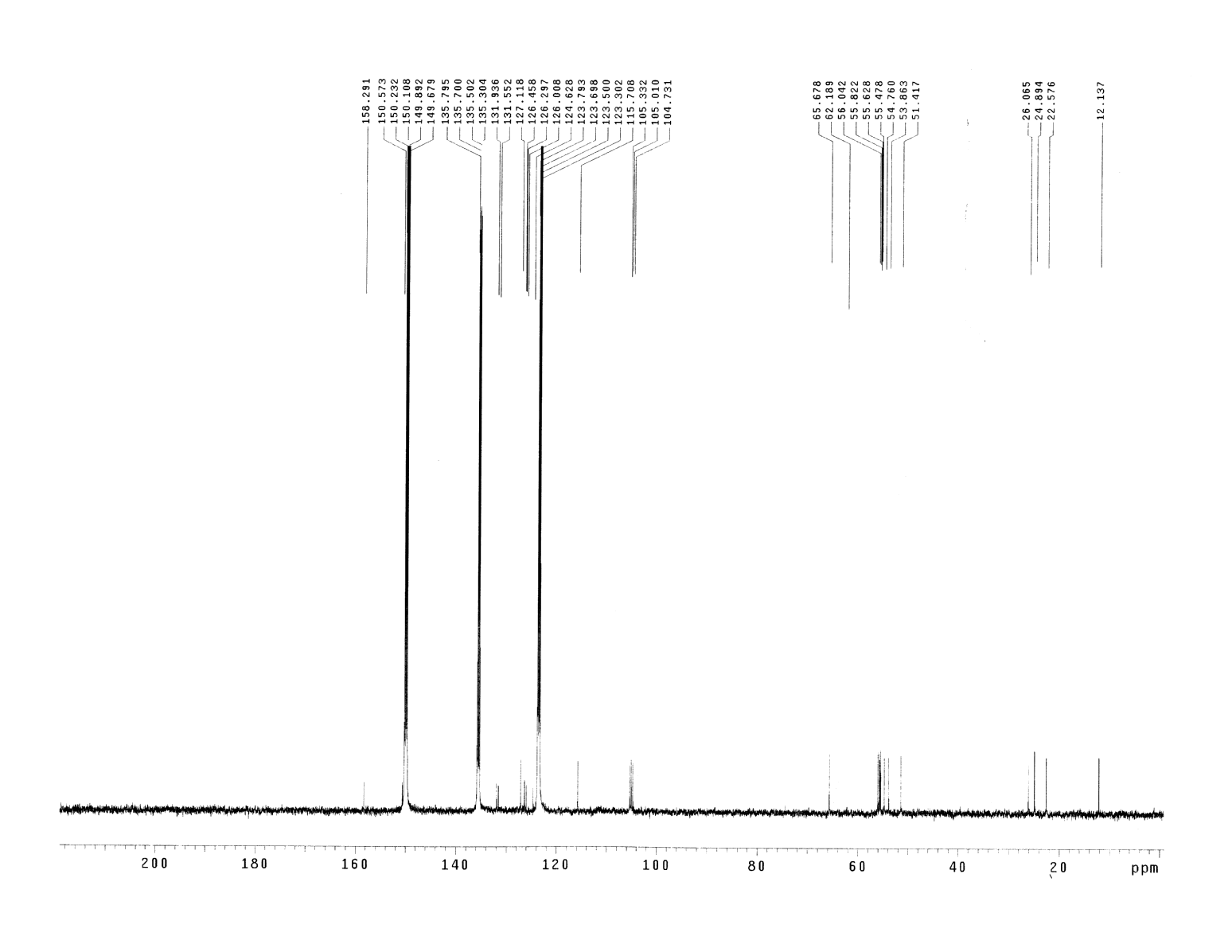
**

**Compound 18**

**
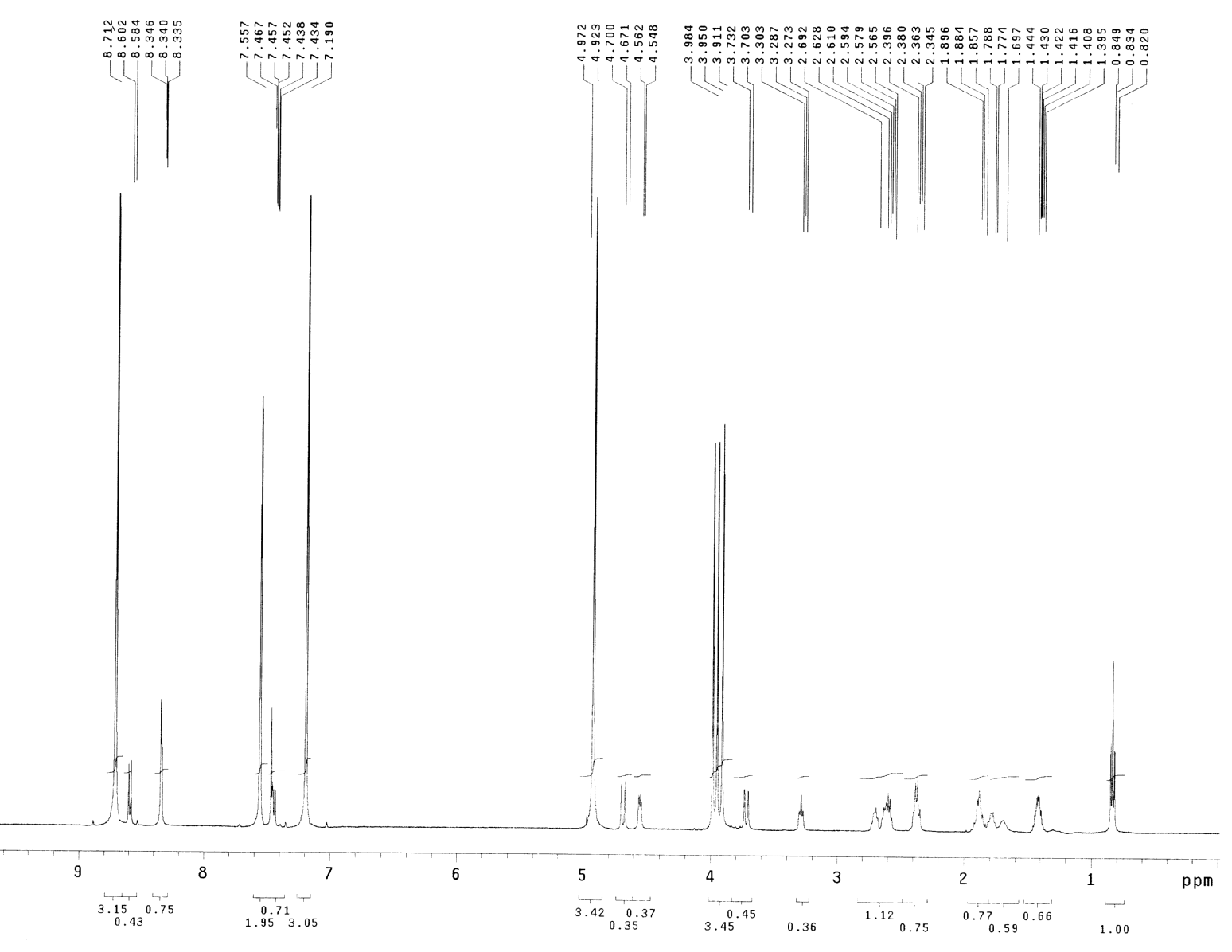
**

**Compound 19**

**
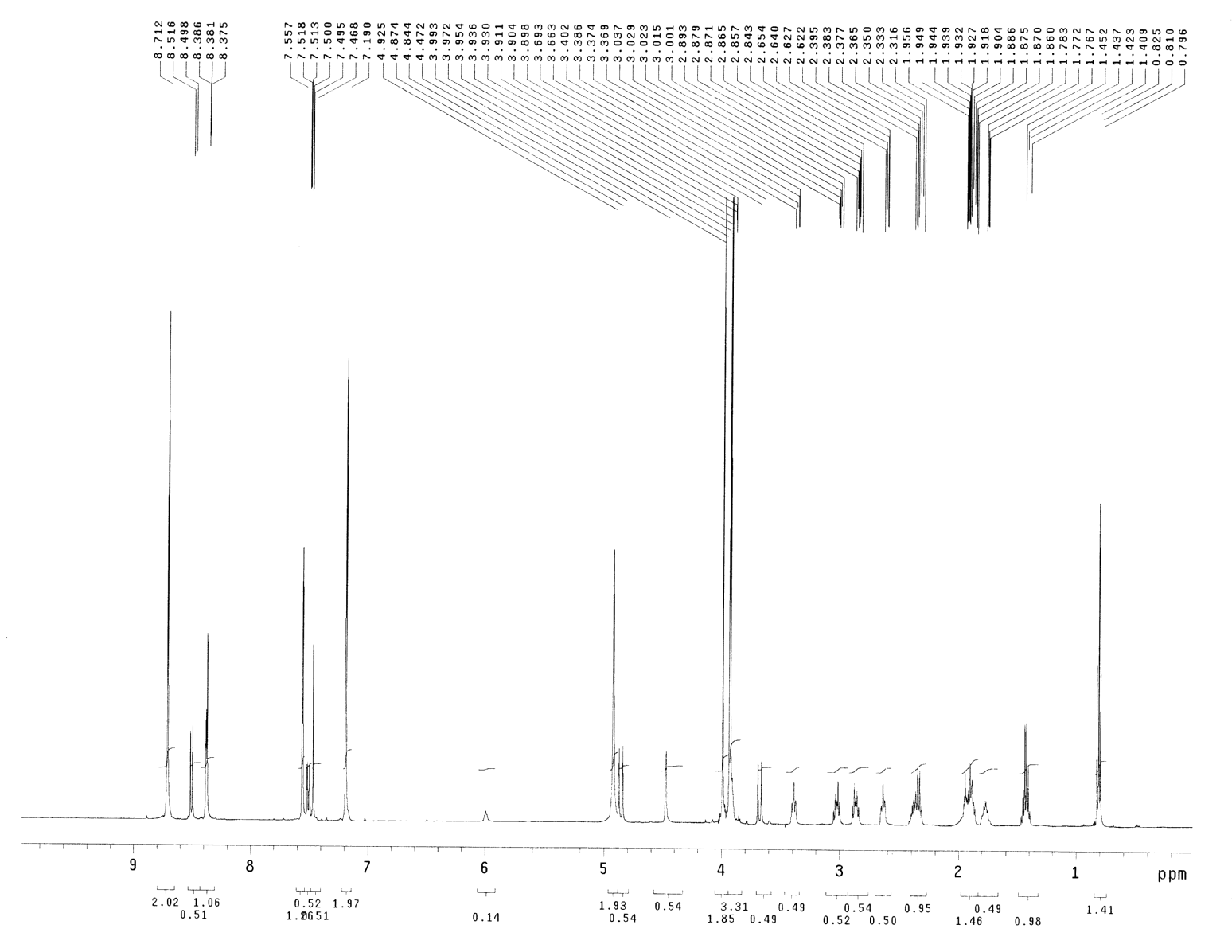
**

**Compound 20**

**
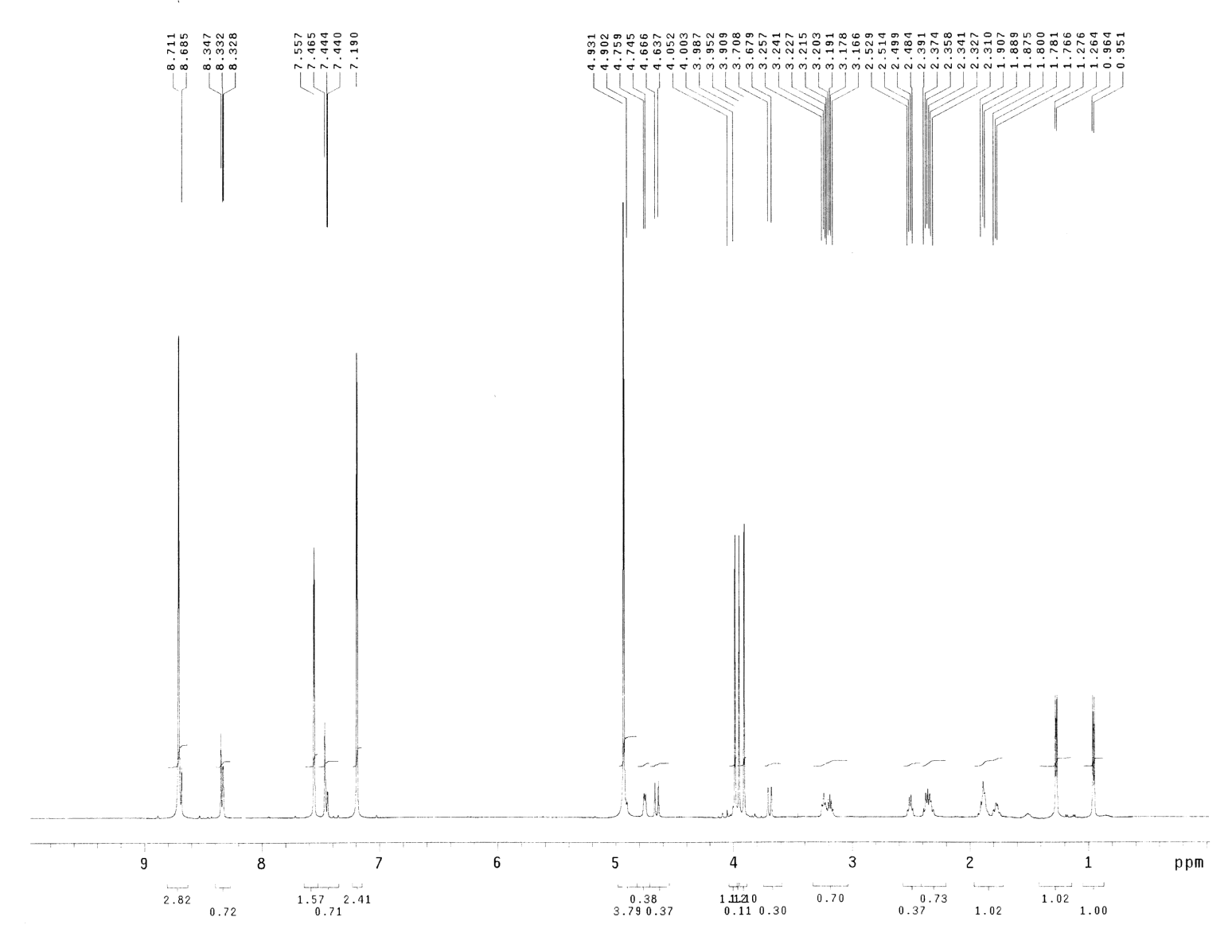
**

**
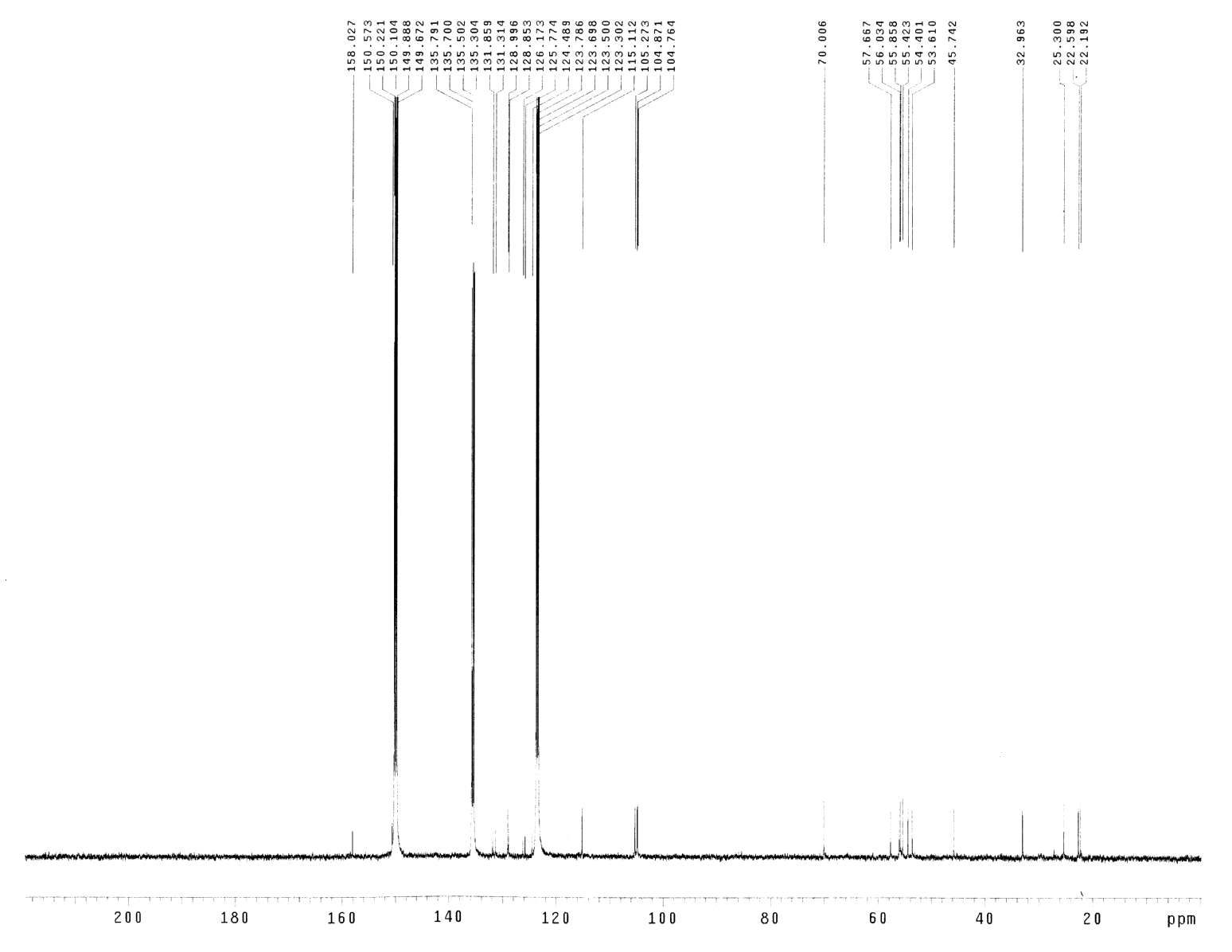
**

**Compound 21**

**
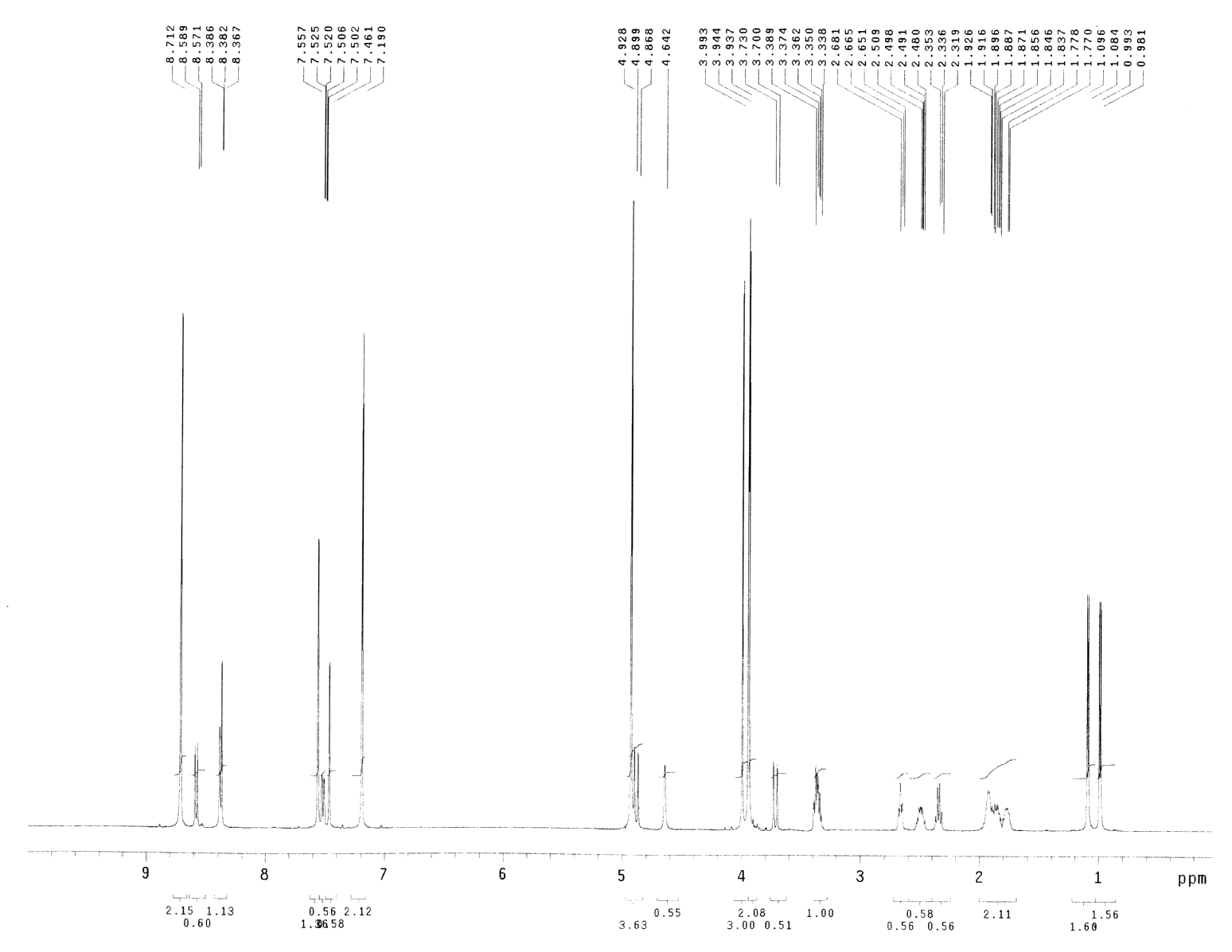
**

**
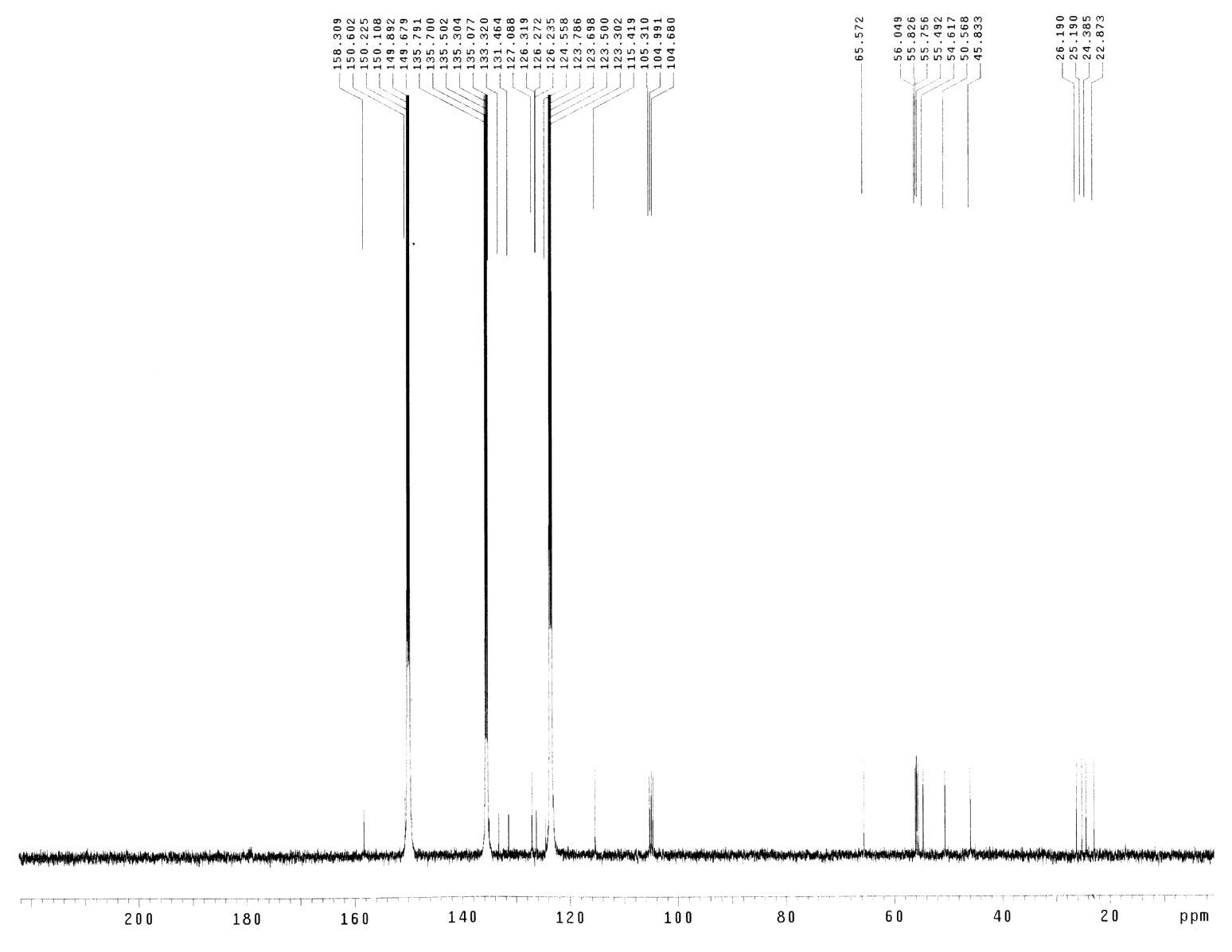
**

**Compound 22**

**
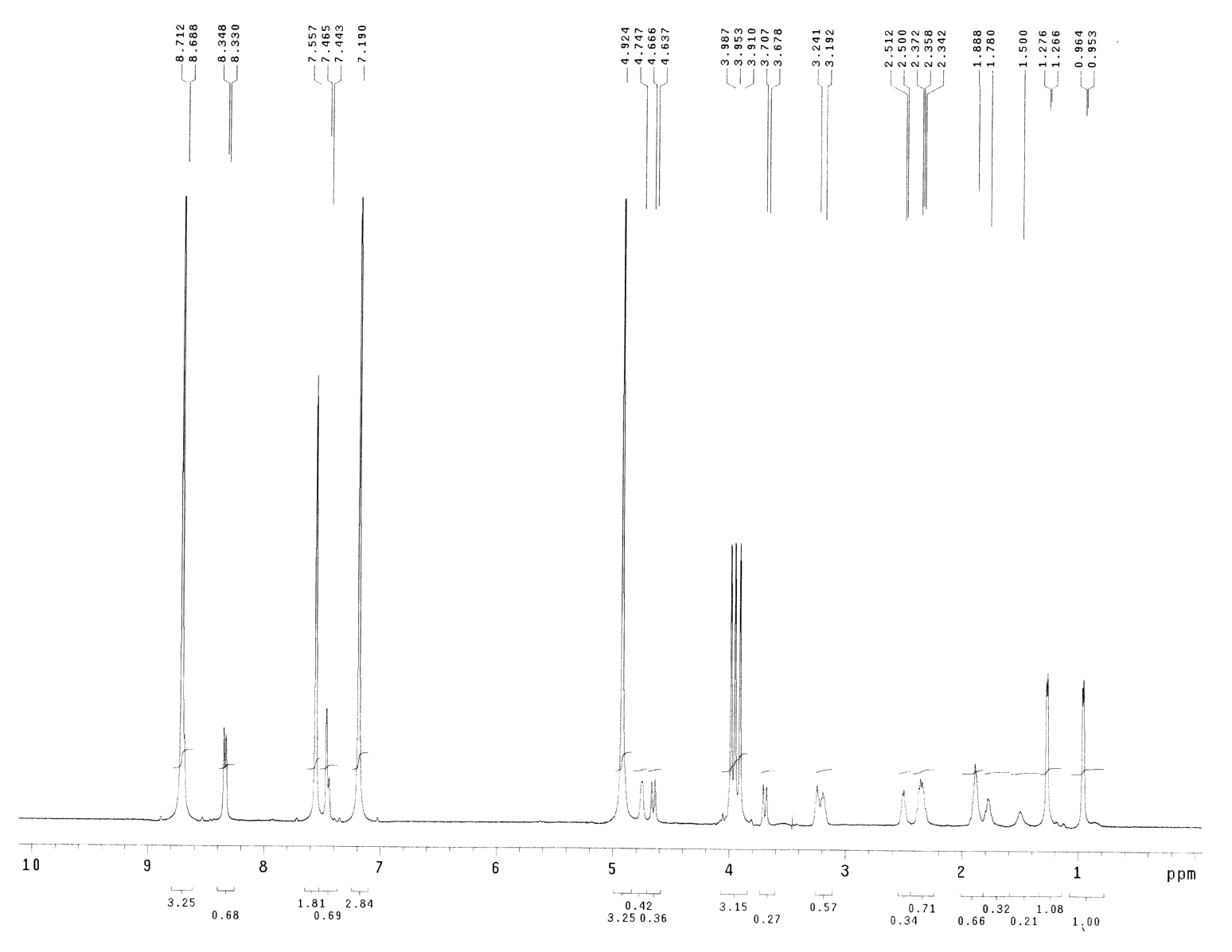
**

**Compound 23**

**
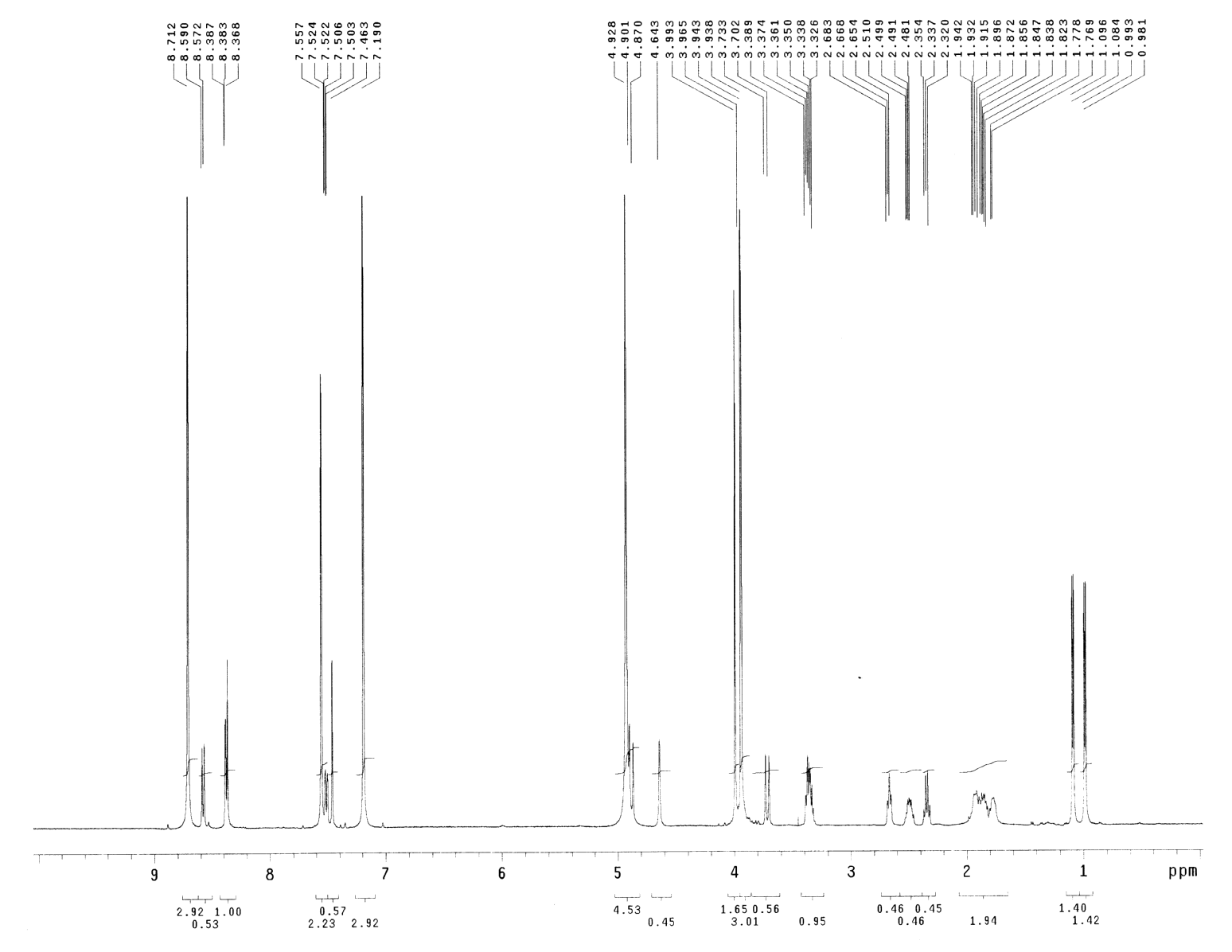
**

**Compound 24**

**
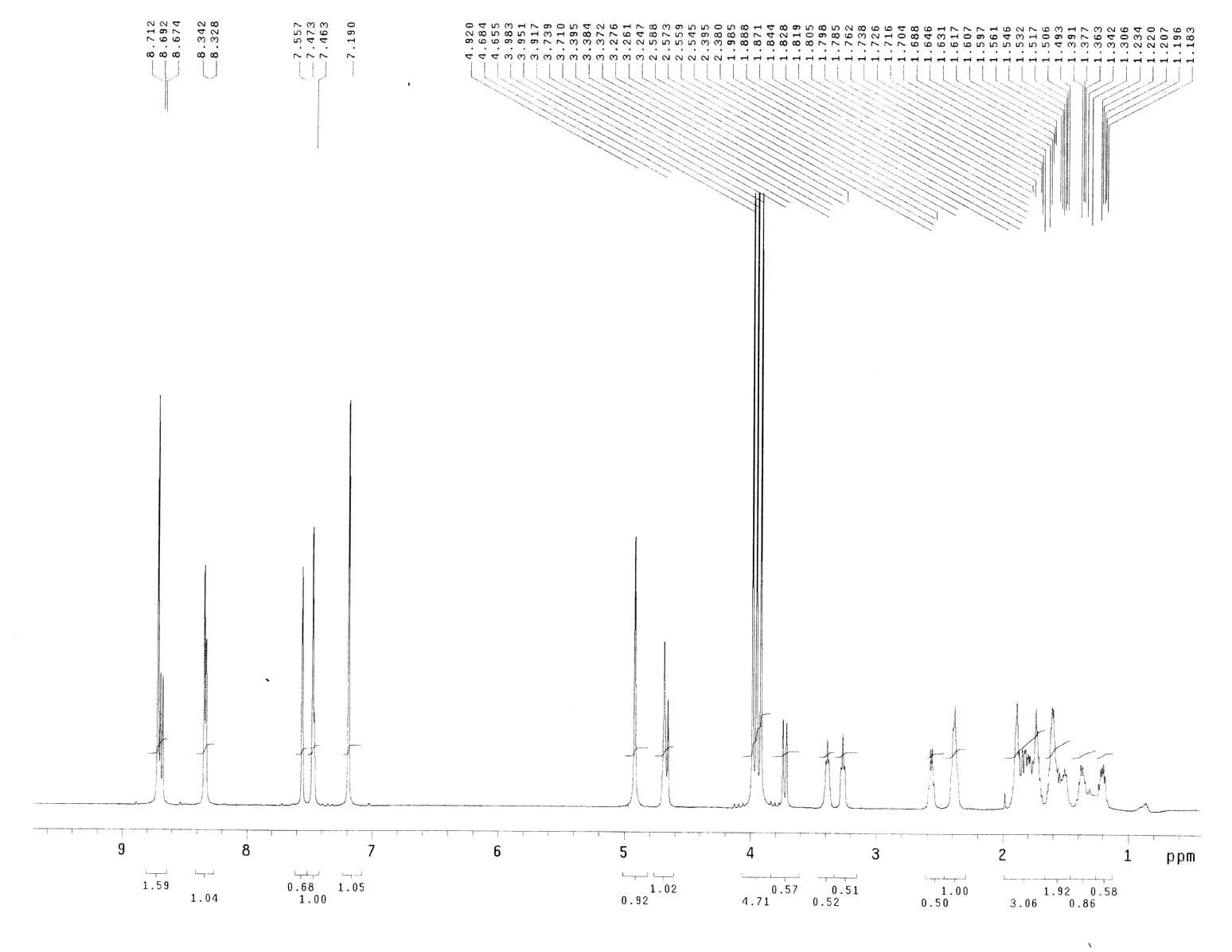
**

**
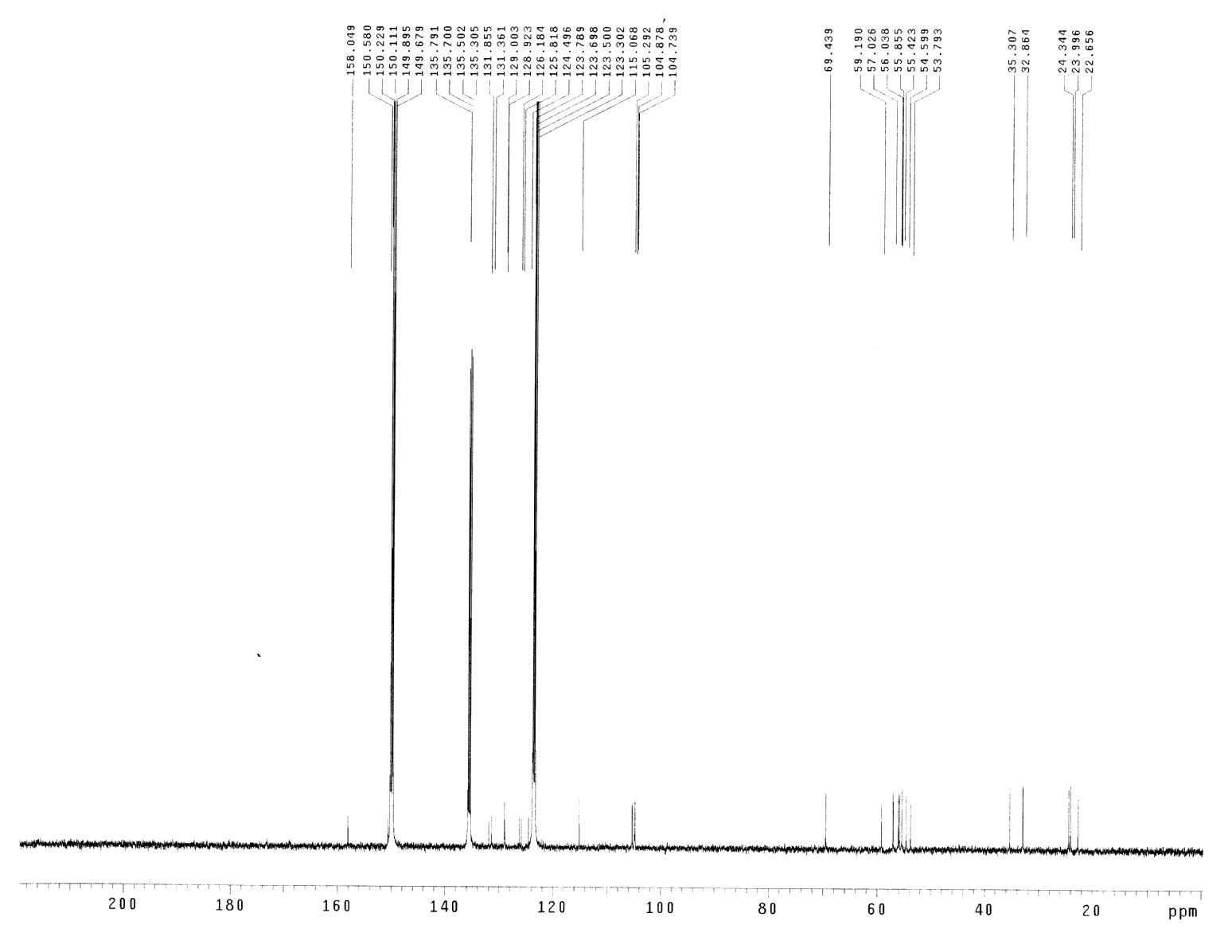
**

**Compound 25**

**
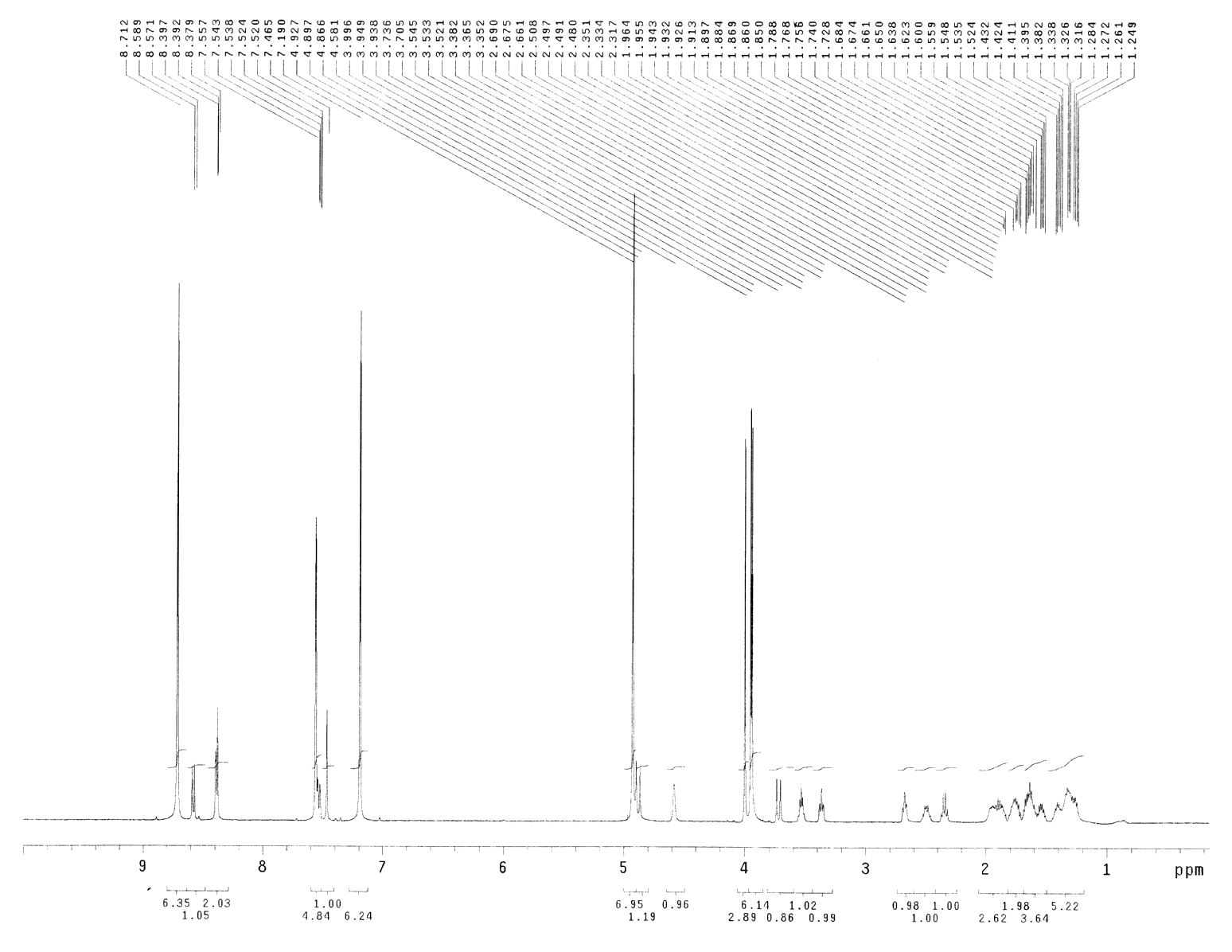
**

**
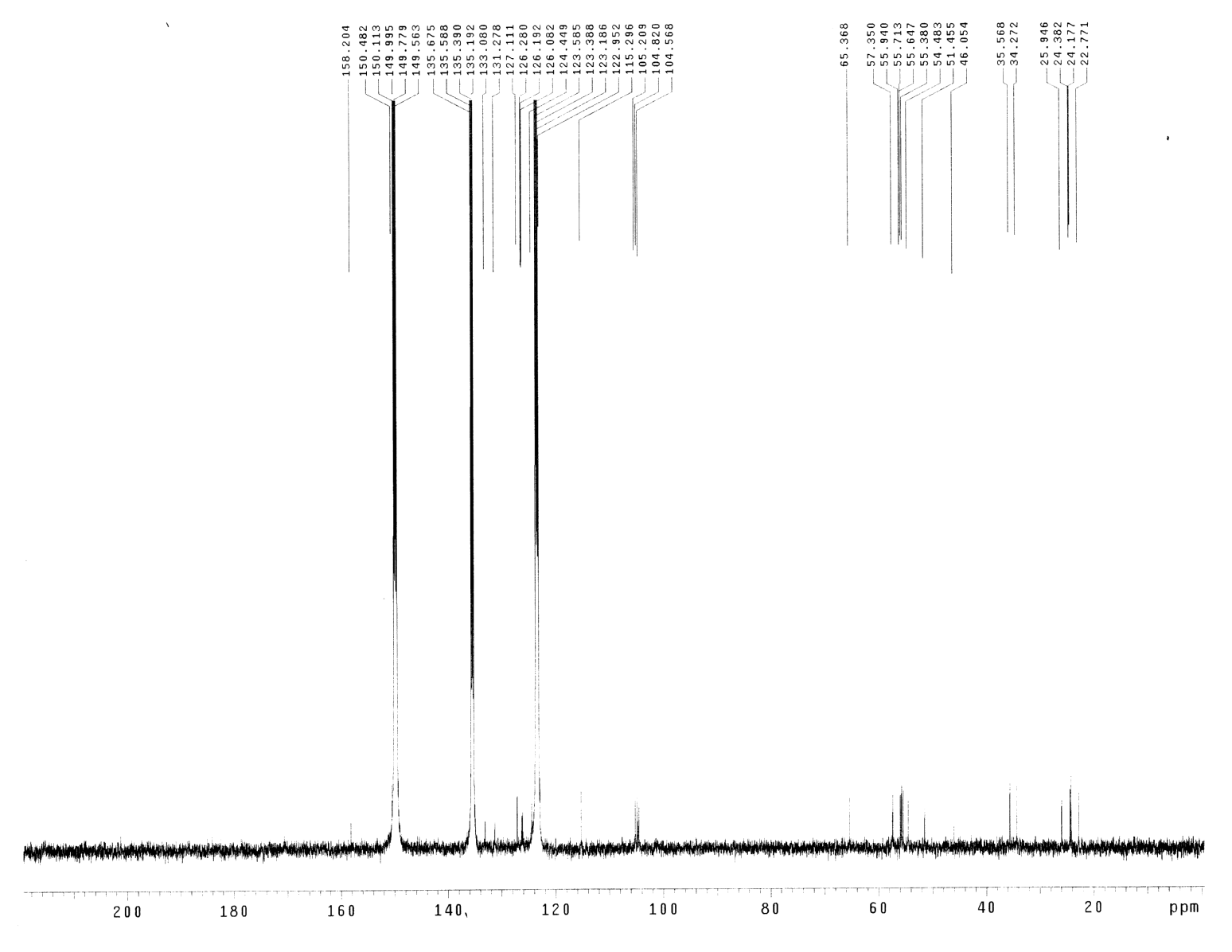
**

**Compound 26**

**
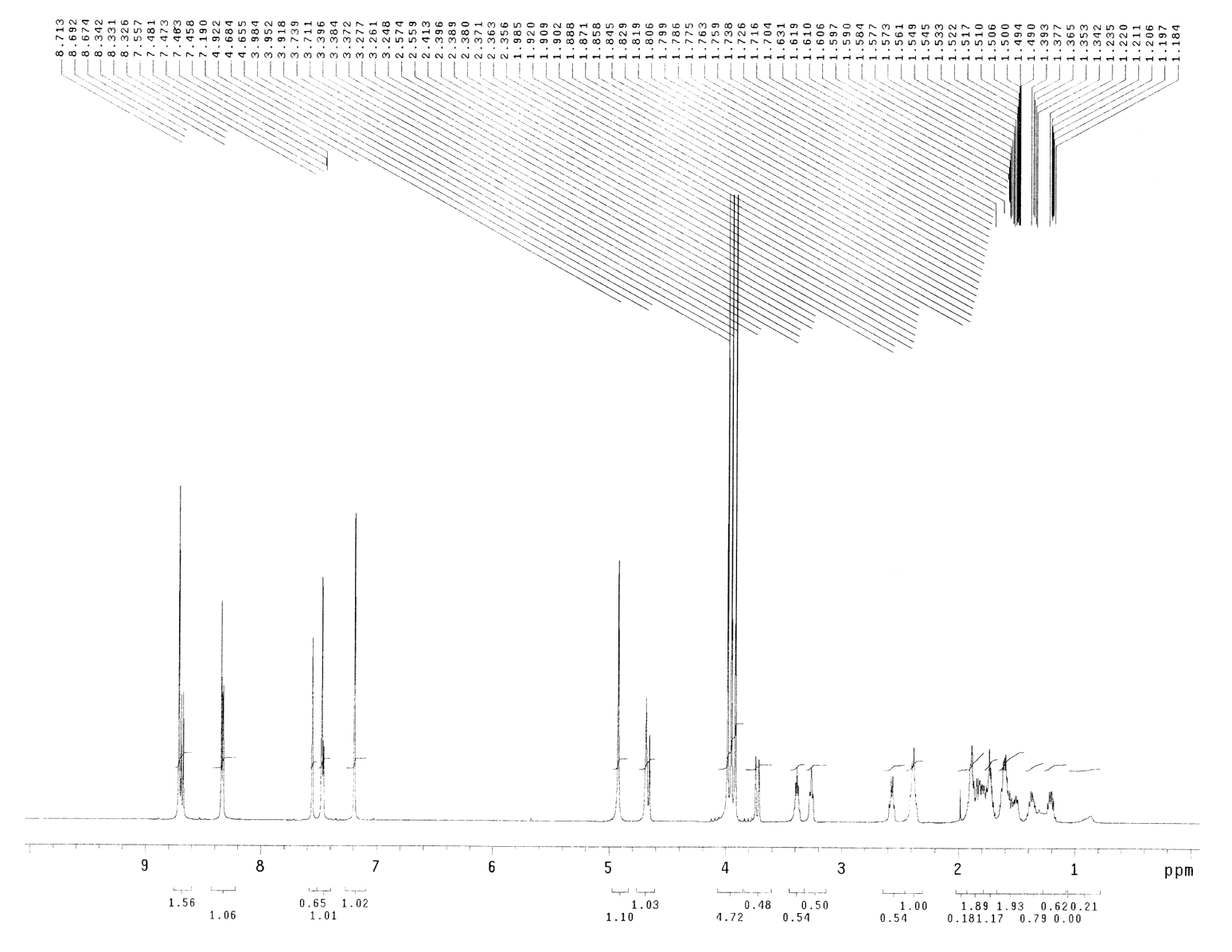
**

**Compound 27**

**
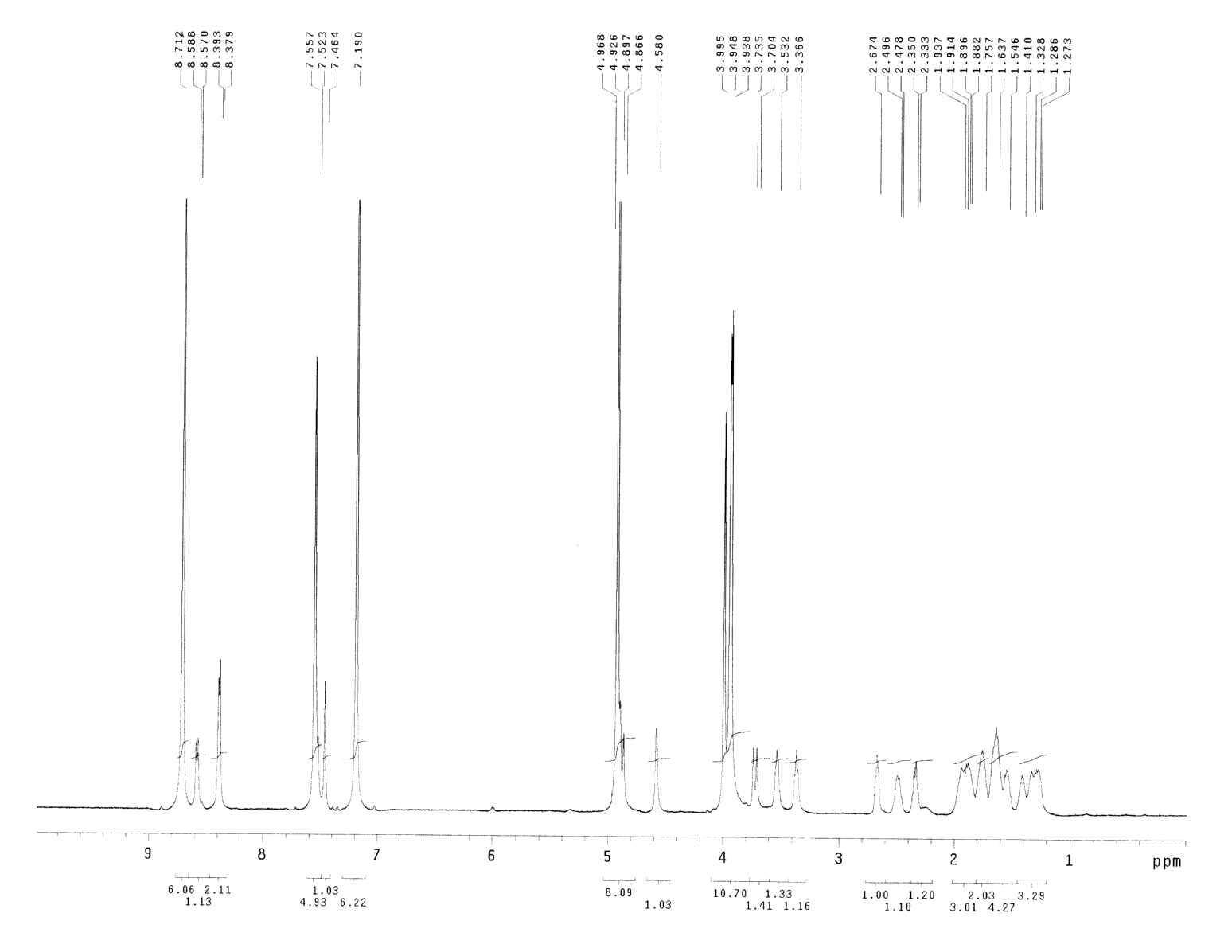
**

**Compound 28**

**
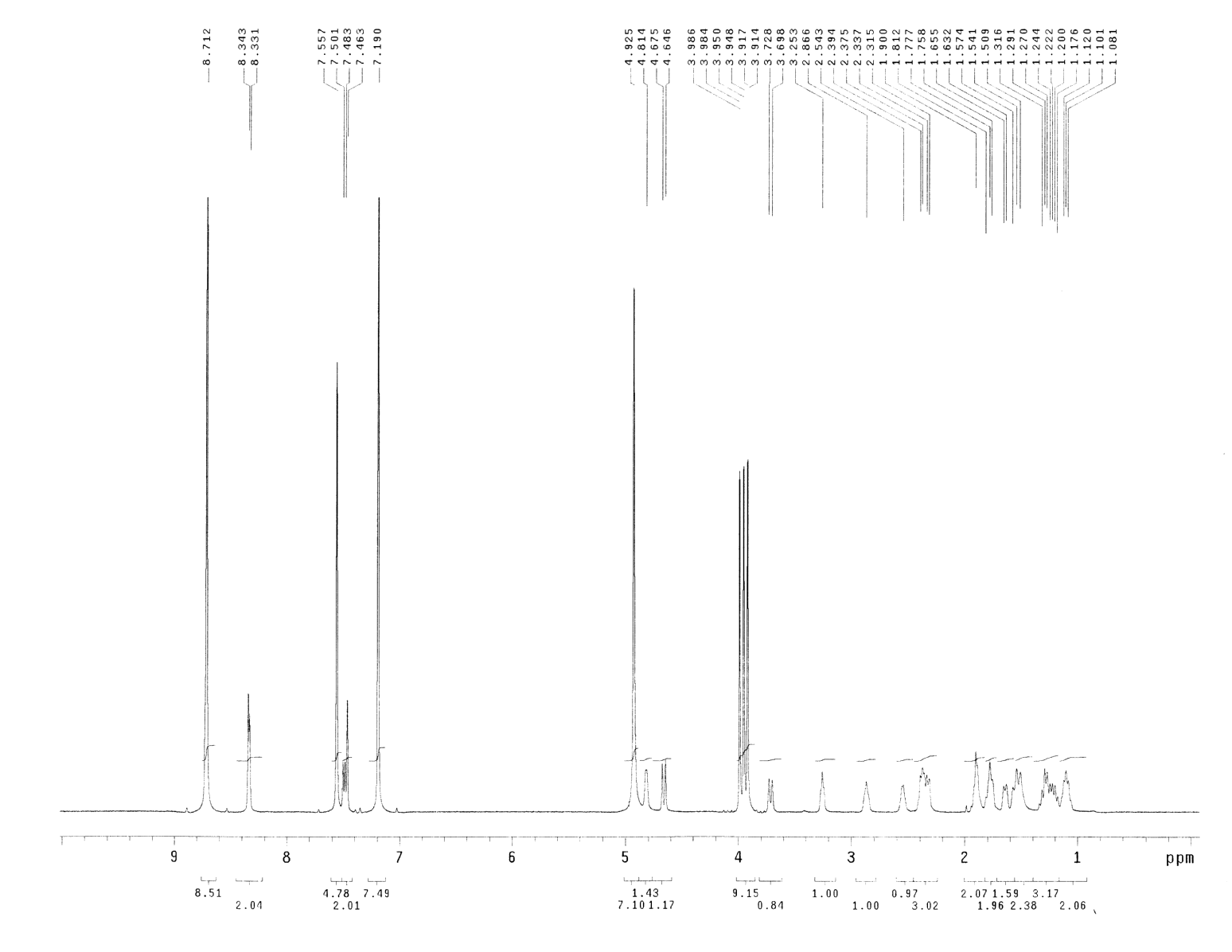
**

**
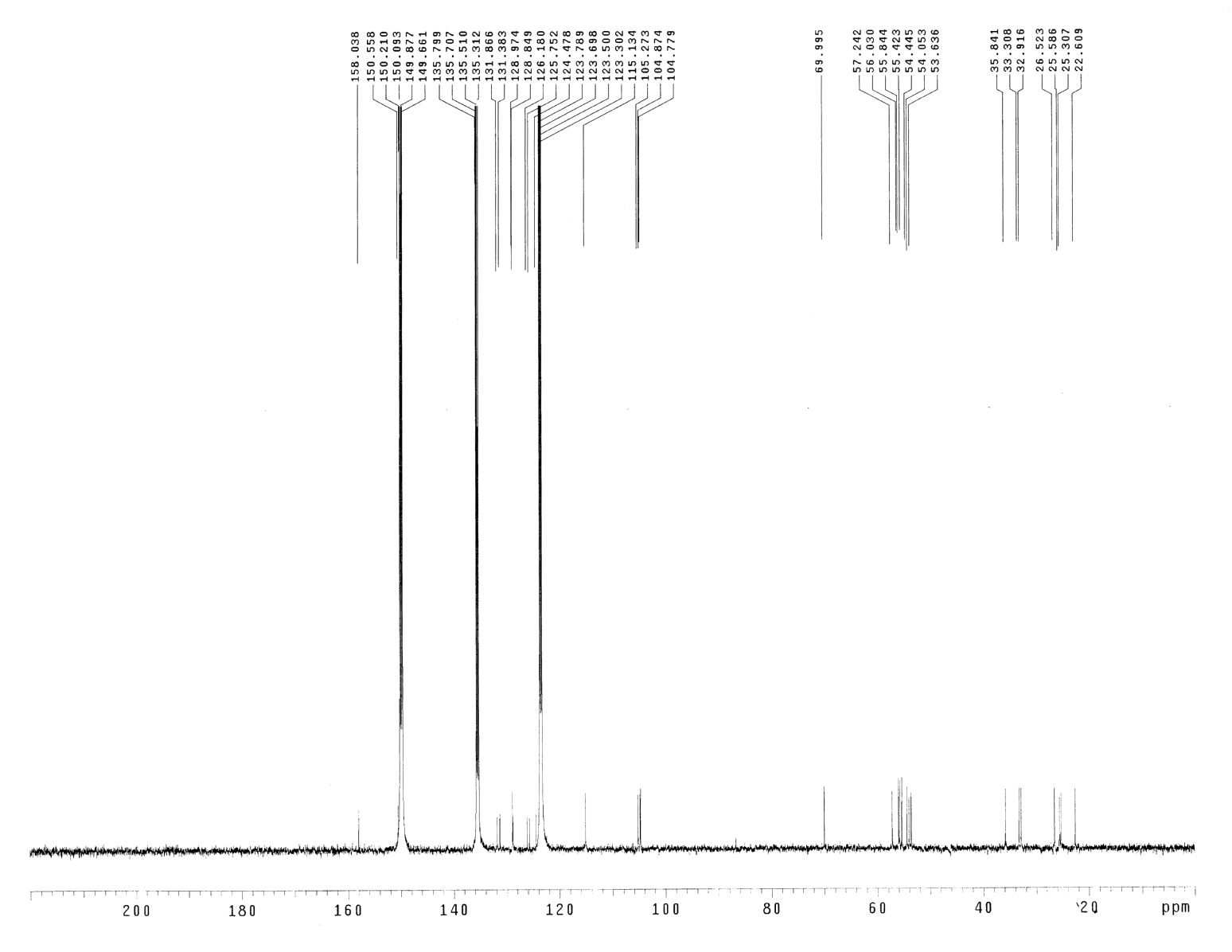
**

**Compound 29**

**
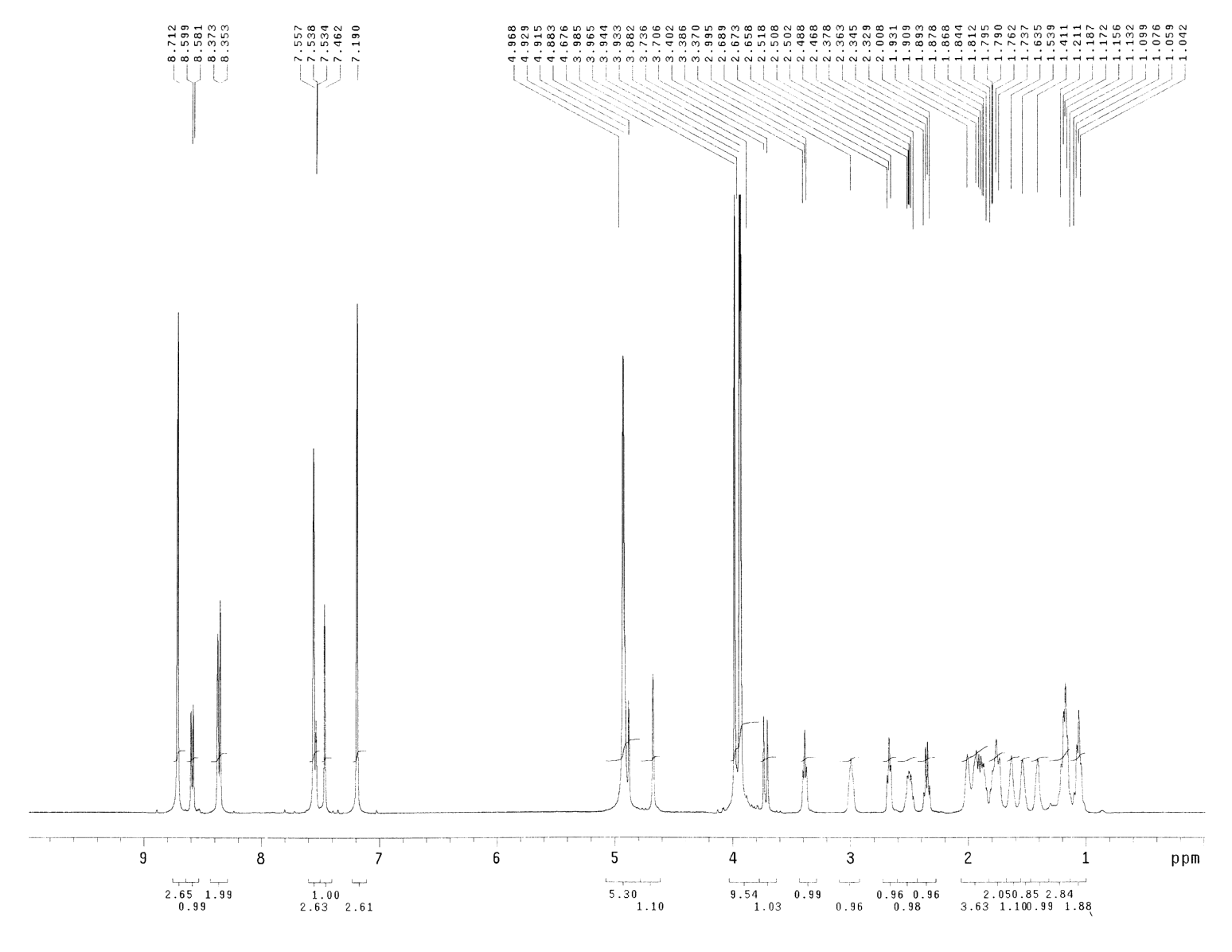
**

**
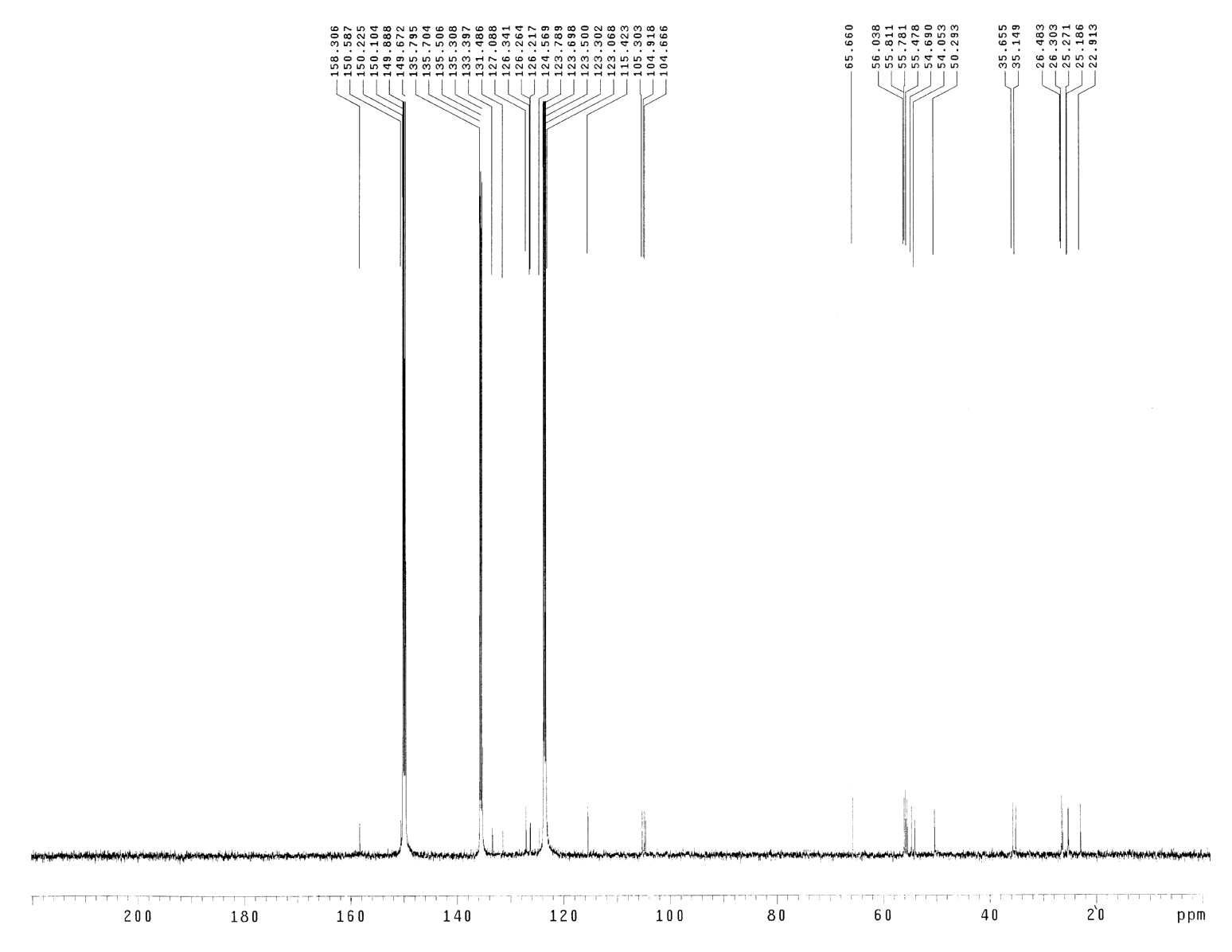
**

**Compound 30**

**
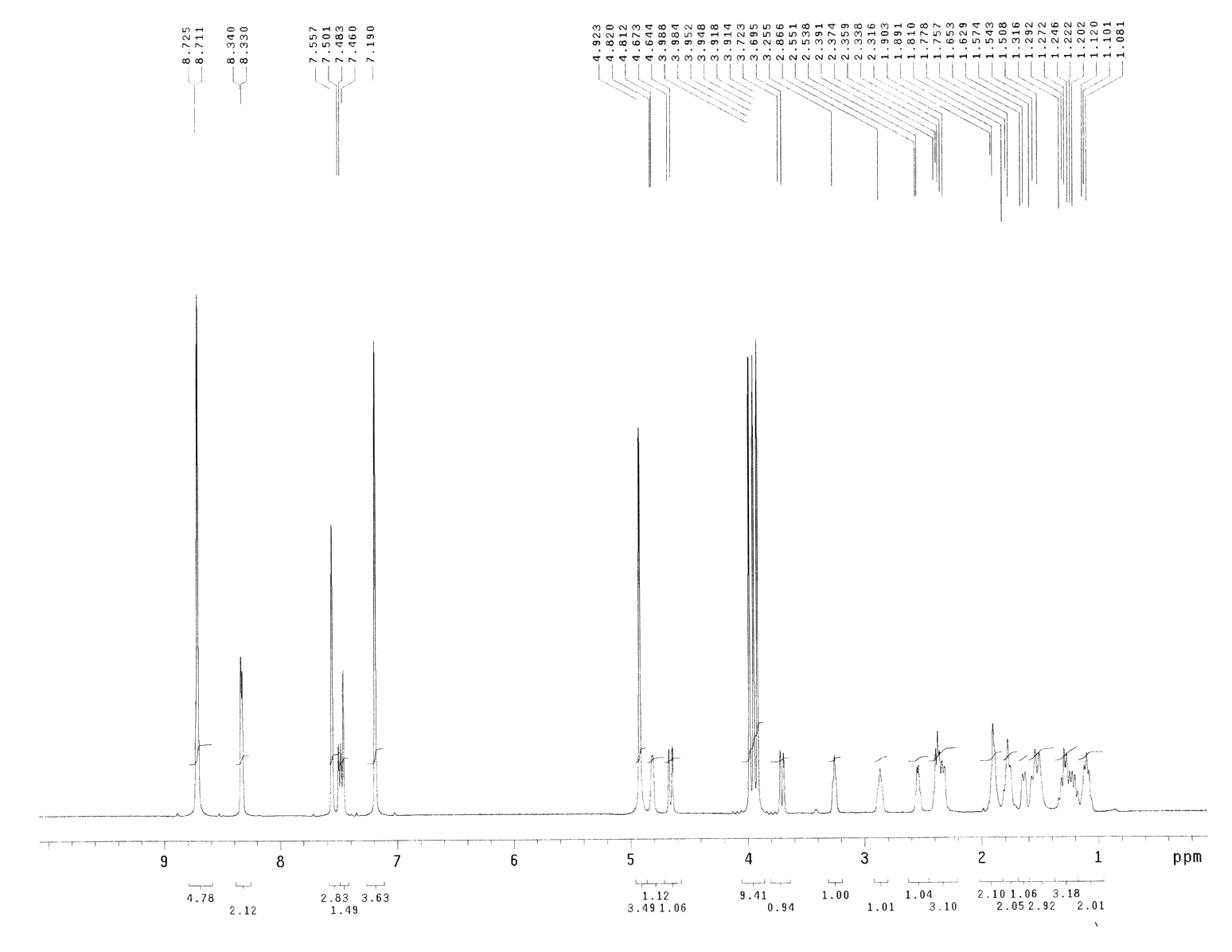
**

**Compound 31**

**
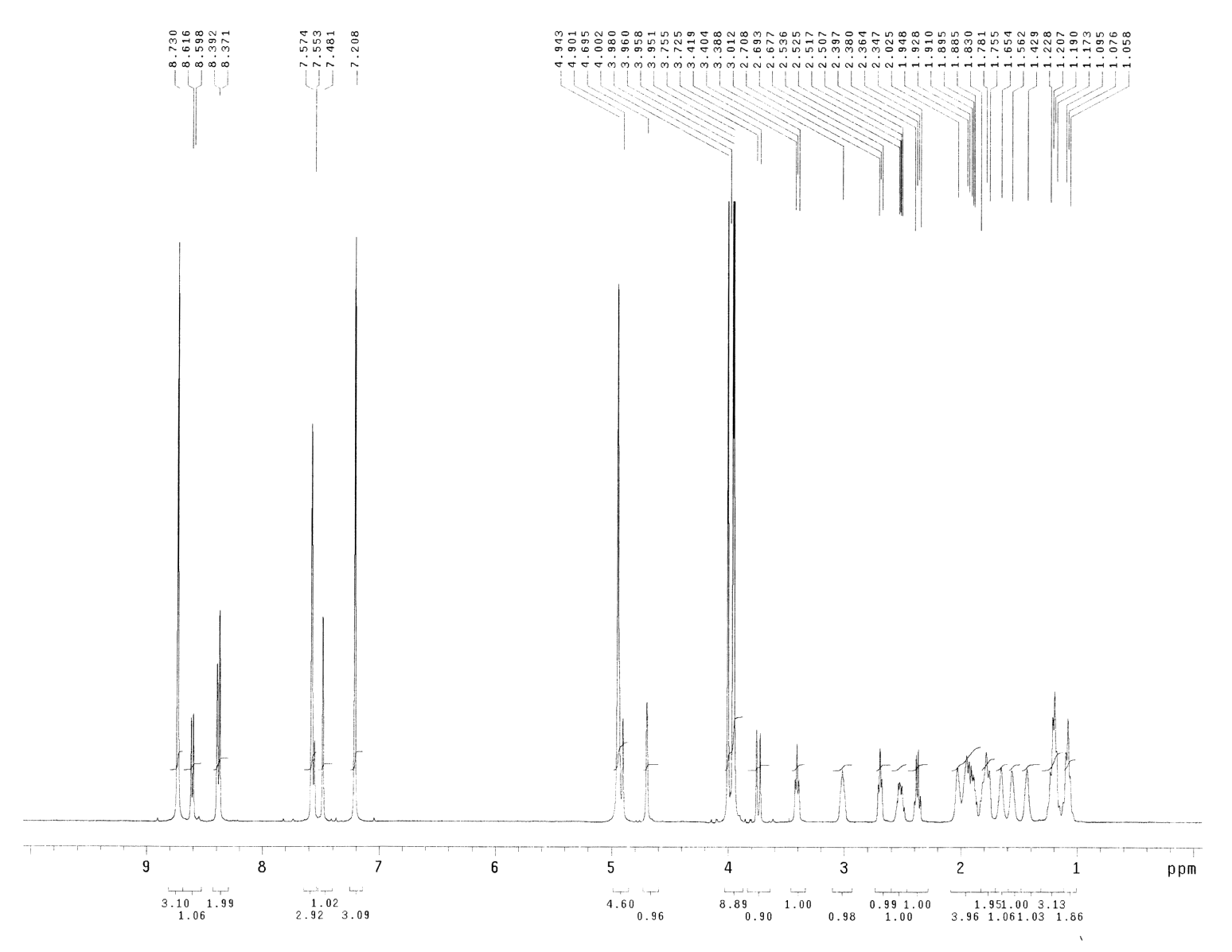
**

**Compound 32**

**
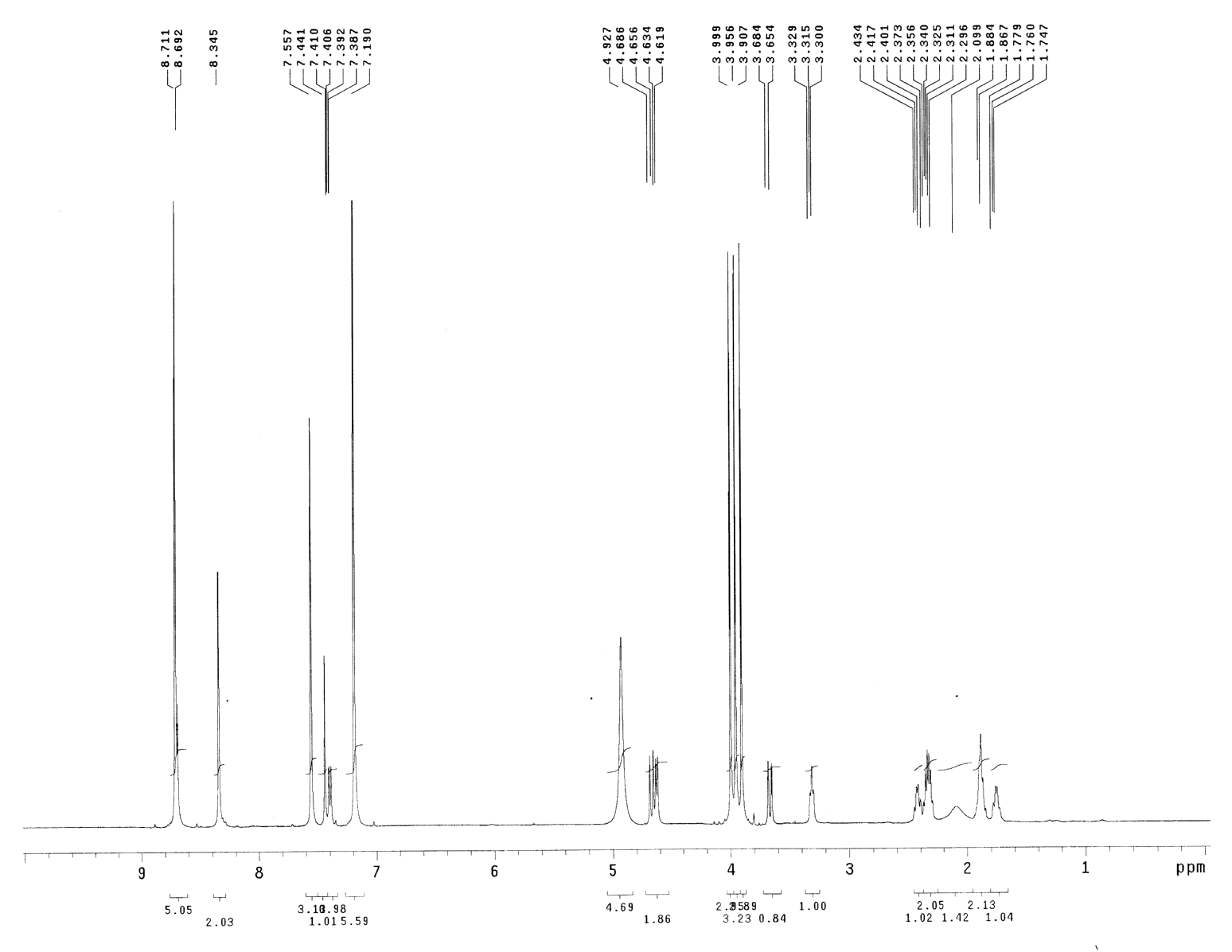
**

**
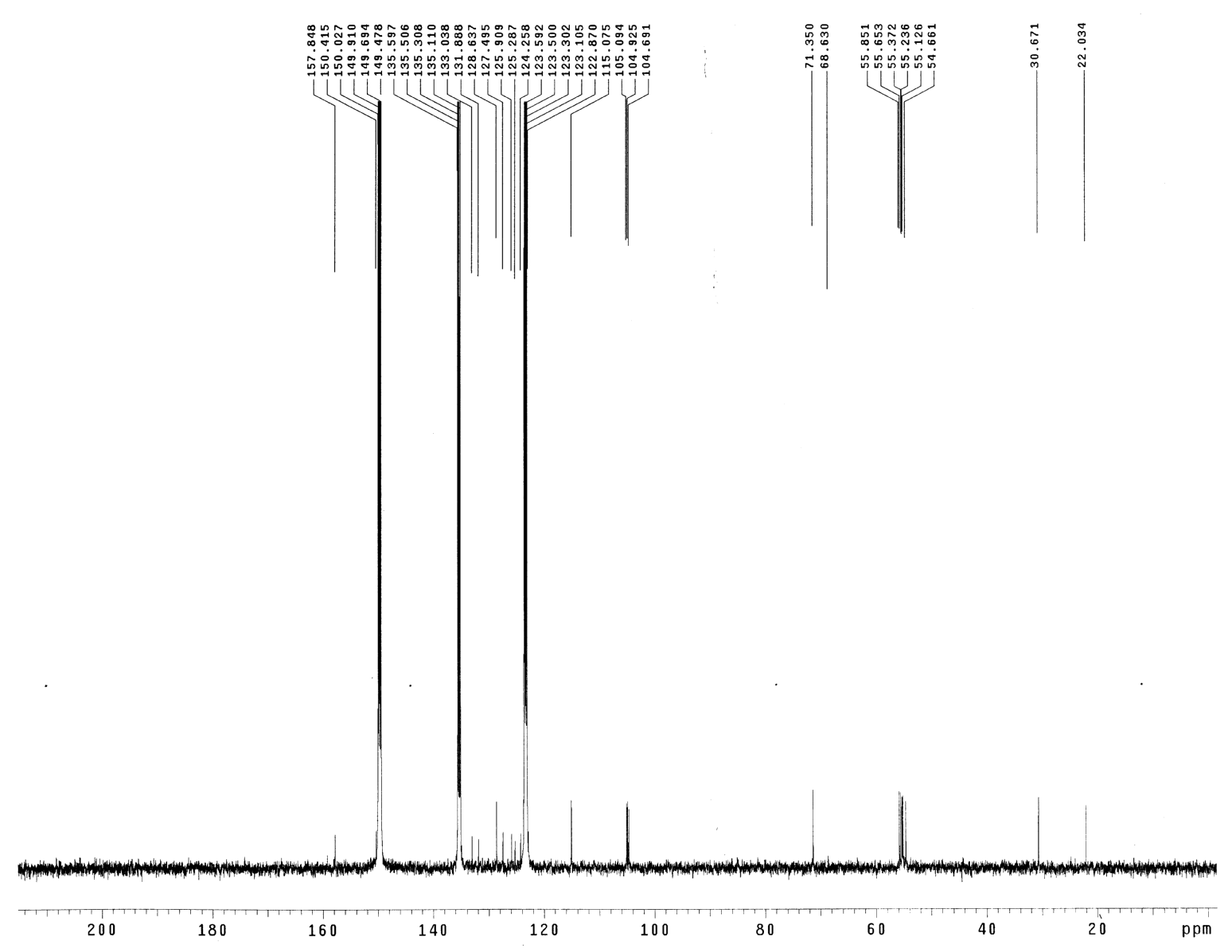
**

**Compound 33**

**
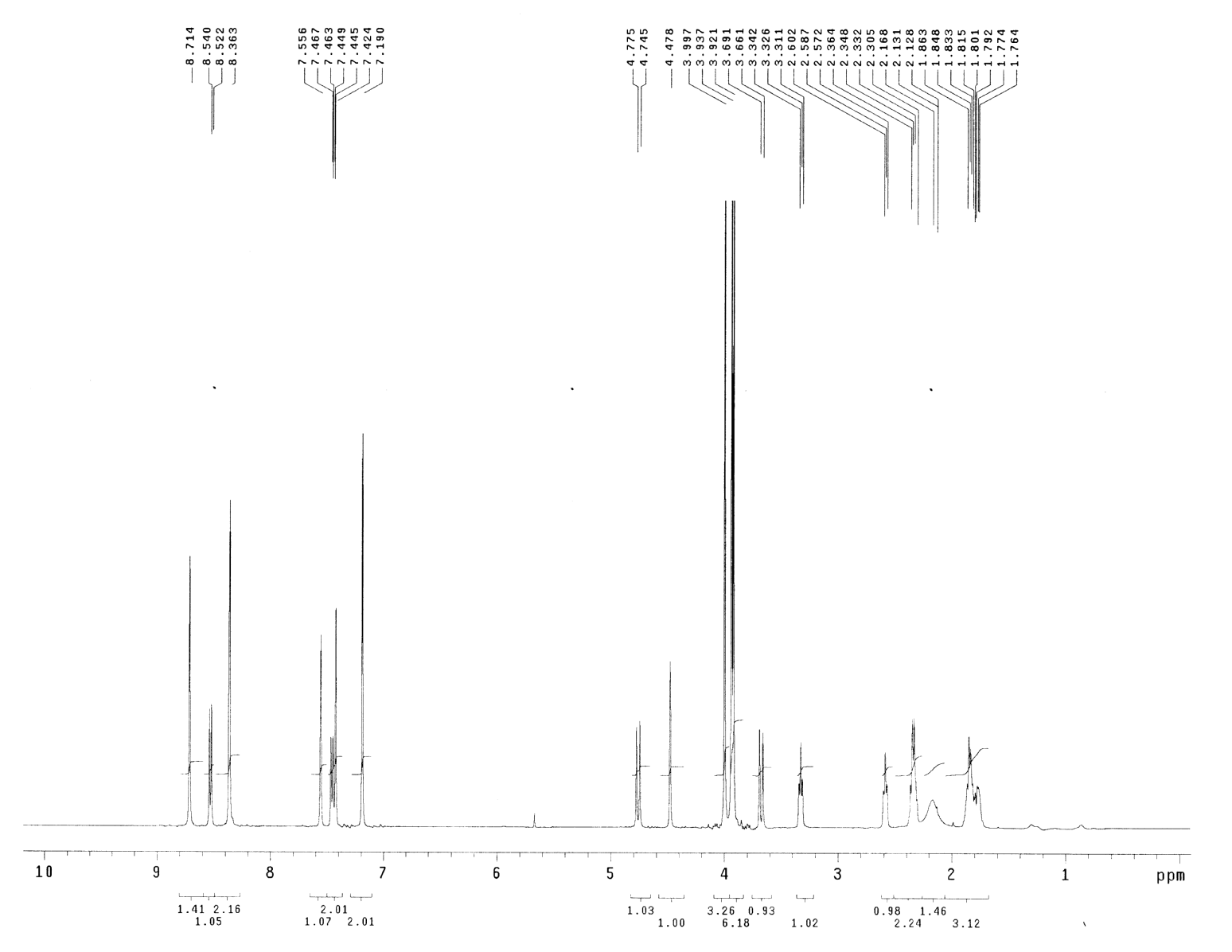
**

**
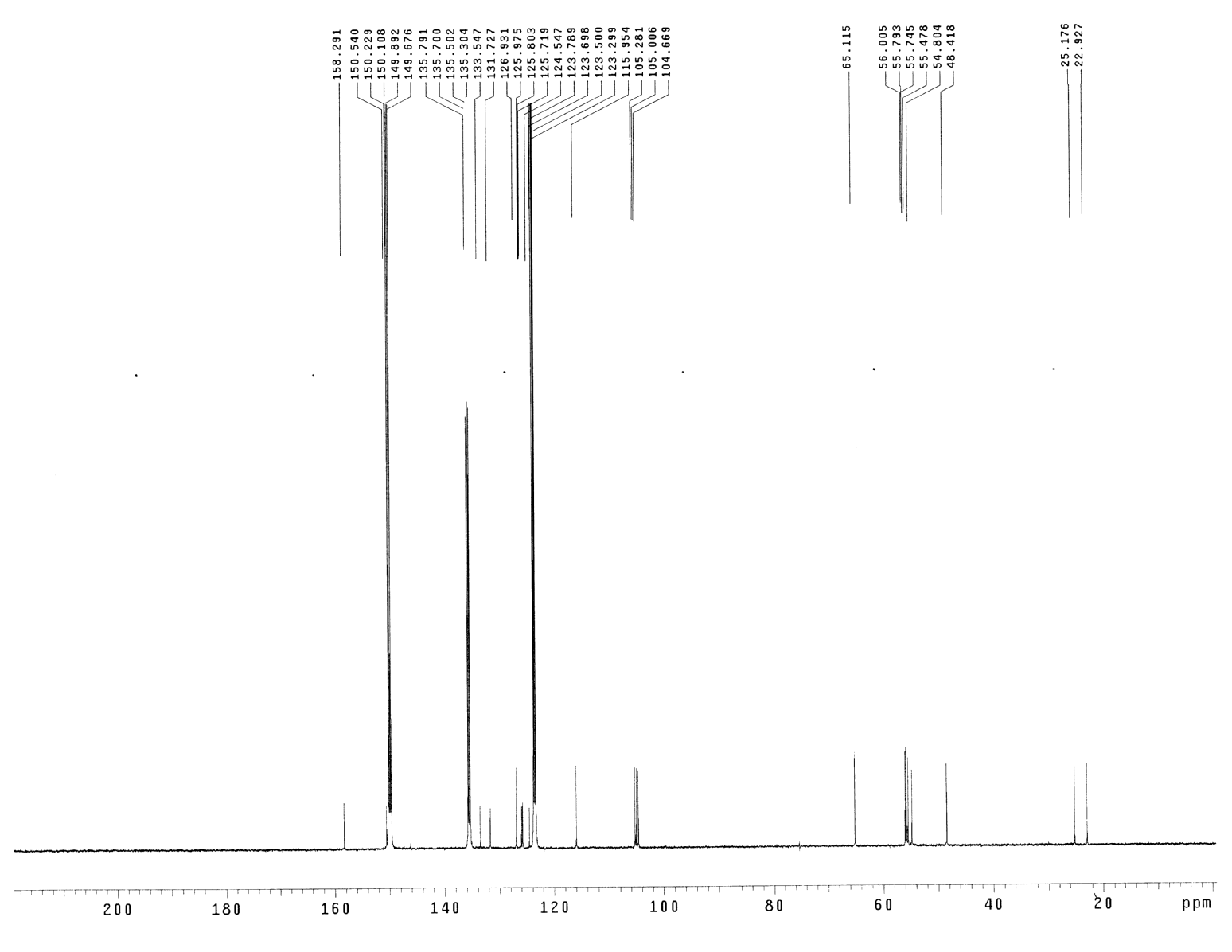
**

**Compound 34**

**
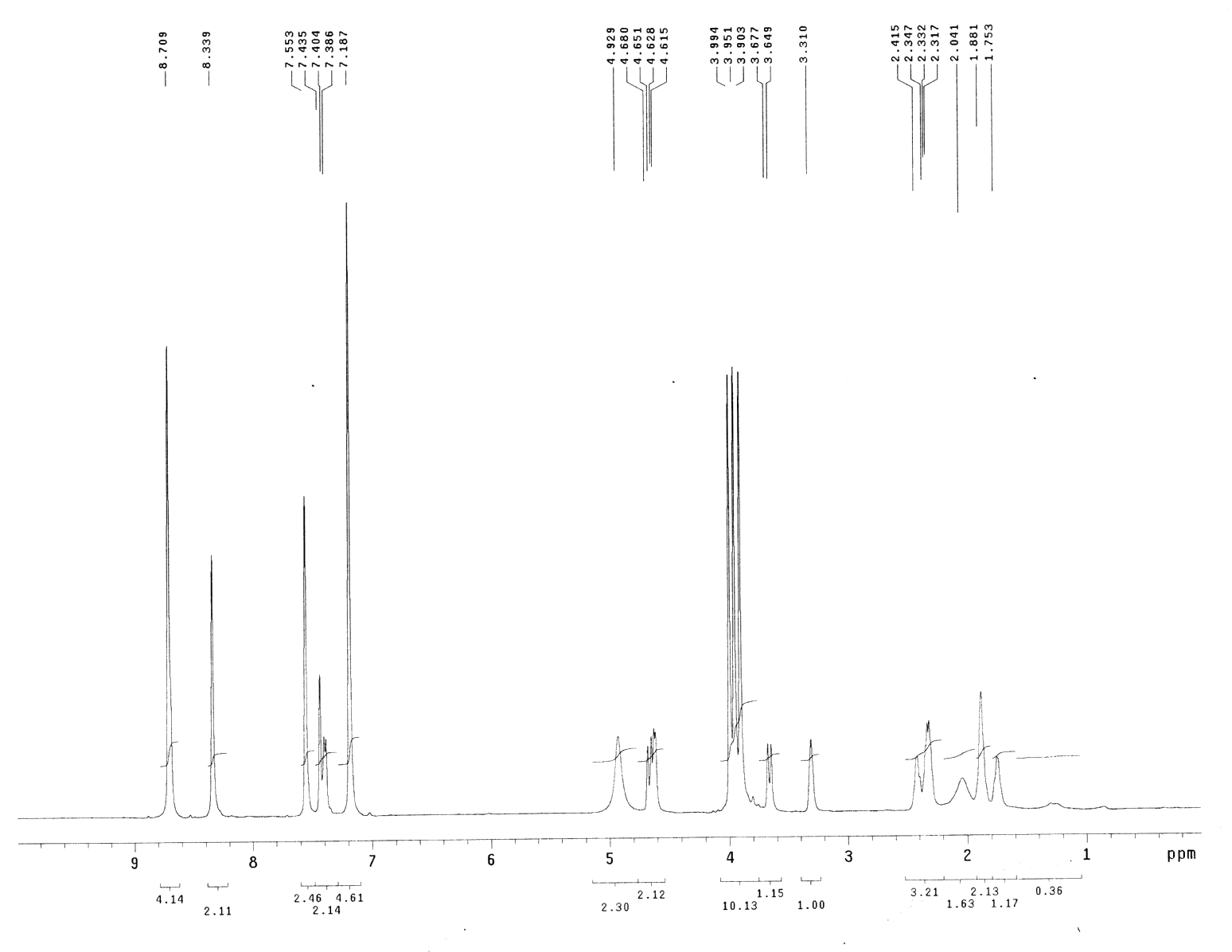
**

**Compound 35**

**
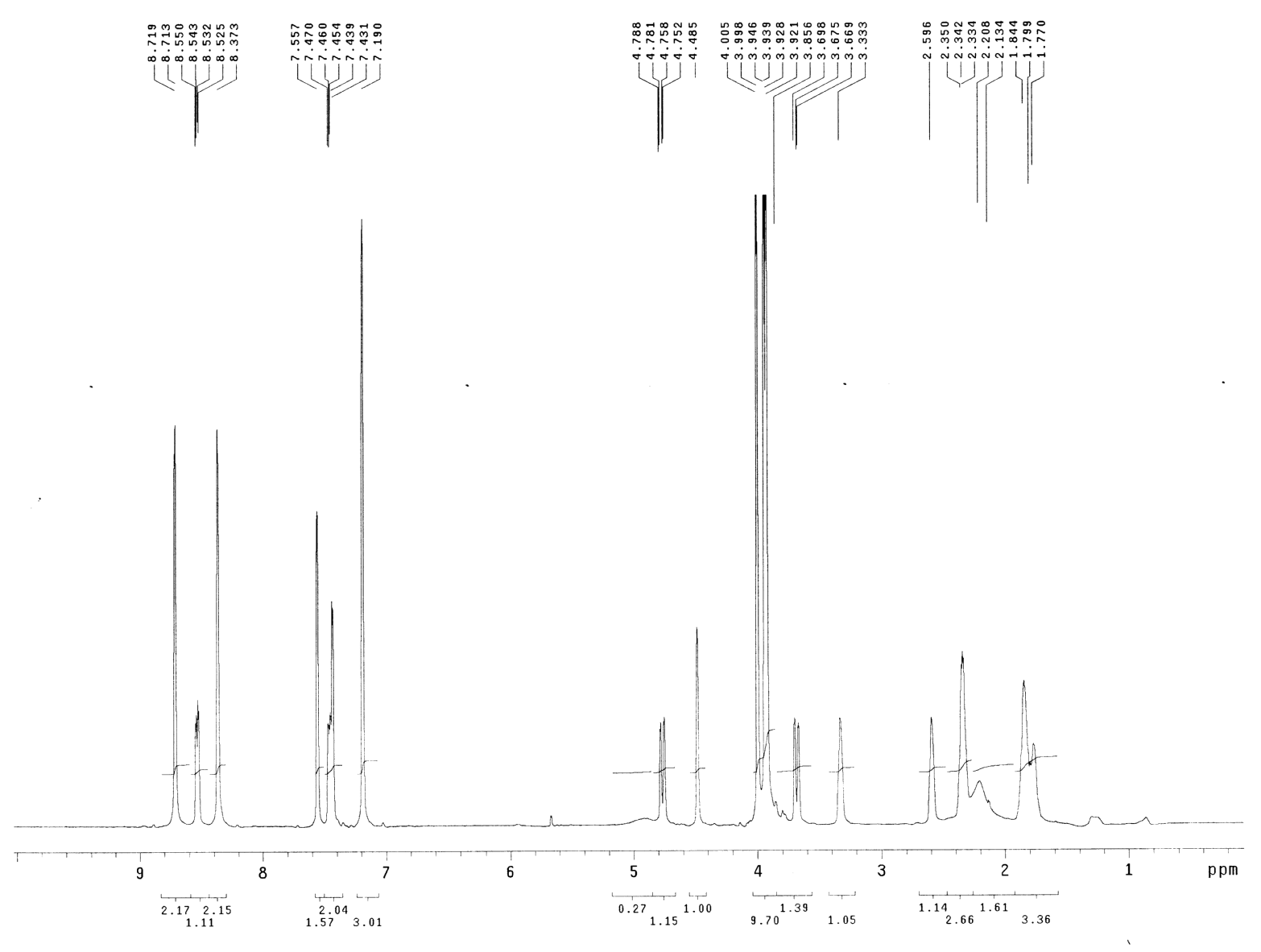
**
