# Supplementary material for: Synthesis, Biological Evaluation and Mechanism Studies of Deoxytylophorinine and Its Derivatives as Potential Anticancer Agents
Source: PLoS One. 2012 Jan 19;7(1):e30342. doi: 10.1371/journal.pone.0030342 (PMC3261902; doi:10.1371/journal.pone.0030342)
Supplement: Figure S4 — Purity data for compounds 1–35. (DOC) [file pone.0030342.s004.doc]

**Figure S4. HPLC data for the compounds 1-35**

**Compound 1**

Xtimate C_18_ 4.6 × 250 mm, 5 μm. 38% MeCN/H_2_O (0.06 M NH_4_H_2_PO_4_, 0.2% Et_3_N, 2.5% THF)

| Peak | Area | Time | Tailing | Resolution | Area% |
| --- | --- | --- | --- | --- | --- |
| 1 | 3551 | 4.236 | 1.330 | -- | 0.0091 |
| 2 | 5661 | 7.311 | 1.430 | 13.041 | 0.0145 |
| 3 | 9700 | 8.541 | 1.056 | 4.329 | 0.0248 |
| 4 | 20826 | 11.484 | 1.113 | 8.555 | 0.0533 |
| 5 | 39029070 | 15.710 | 1.060 | 8.975 | 99.8401 |
| 6 | 22787 | 21.498 | 1.059 | 9.785 | 0.0583 |

**Compound 2**

Xtimate C_18_ 4.6 × 250 mm, 5 μm. 38% MeCN/H_2_O (0.06 M NH_4_H_2_PO_4_, 0.2% Et_3_N, 2.5% THF)

| Peak | Area | Time | Tailing | Resolution | Area% |
| --- | --- | --- | --- | --- | --- |
| 1 | 2040 | 4.268 | 2.255 | -- | 0.0127 |
| 2 | 4256 | 8.545 | 0.997 | 17.172 | 0.0265 |
| 3 | 7037 | 11.466 | 1.199 | 8.511 | 0.0438 |
| 4 | 16058846 | 15.710 | 1.051 | 9.335 | 99.8672 |
| 5 | 8020 | 21.512 | 1.013 | 10.474 | 0.0499 |

**Compound 3**

Xtimate C_18_ 4.6 × 250 mm, 5 μm. 46% MeCN/H_2_O (0.08 M NH_4_H_2_PO_4_, 0.2% Et_3_N)

| Peak | Area | Time | Tailing | Resolution | Area% |
| --- | --- | --- | --- | --- | --- |
| 1 | 47750 | 4.577 | 2.040 | -- | 0.4406 |
| 2 | 10691360 | 5.775 | 1.306 | 5.374 | 98.6422 |
| 3 | 99417 | 8.659 | 1.048 | 11.704 | 0.9173 |

**Compound 4**

Xtimate C_18_ 4.6 × 250 mm, 5 μm. 46% MeCN/H_2_O (0.08 M NH_4_H_2_PO_4_, 0.2% Et_3_N)

| Peak | Area | Time | Tailing | Resolution | Area% |
| --- | --- | --- | --- | --- | --- |
| 1 | 24458 | 5.021 | 2.288 | -- | 0.2739 |
| 2 | 8851832 | 8.394 | 1.216 | 10.356 | 99.1147 |
| 3 | 22396 | 10.560 | 0.969 | 6.007 | 0.2508 |
| 4 | 32209 | 11.600 | 1.072 | 2.711 | 0.3606 |

**Compound 5**

Xtimate C_18_ 4.6 × 250 mm, 5 μm. 46% MeCN/H_2_O (0.08 M NH_4_H_2_PO_4_, 0.2% Et_3_N)

| Peak | Area | Time | Tailing | Resolution | Area% |
| --- | --- | --- | --- | --- | --- |
| 1 | 24879 | 4.332 | 0.960 | -- | 0.1151 |
| 2 | 13992 | 4.604 | 1.165 | 1.231 | 0.0647 |
| 3 | 6926 | 6.090 | 1.006 | 6.950 | 0.0320 |
| 4 | 1335 | 6.880 | -- | 1.648 | 0.0062 |
| 5 | 79205 | 7.156 | 1.202 | 0.573 | 0.3665 |
| 6 | 21444451 | 8.981 | 1.127 | 6.364 | 99.2199 |
| 7 | 24580 | 13.314 | 1.009 | 12.115 | 0.1137 |
| 8 | 17685 | 15.247 | 1.075 | 4.530 | 0.0818 |

**Compound 6**

Xtimate C_18_ 4.6 × 250 mm, 5 μm. 46% MeCN/H_2_O (0.08 M NH_4_H_2_PO_4_, 0.2% Et_3_N)

| Peak | Area | Time | Tailing | Resolution | Area% |
| --- | --- | --- | --- | --- | --- |
| 1 | 42606 | 4.422 | 1.029 | -- | 0.2002 |
| 2 | 138564 | 7.577 | 1.538 | 13.553 | 0.6510 |
| 3 | 101871 | 9.201 | 1.223 | 5.690 | 0.4786 |
| 4 | 62163 | 9.761 | 1.118 | 1.846 | 0.2920 |
| 5 | 20940850 | 12.759 | 1.010 | 8.594 | 98.3783 |

**Compound 7**

Xtimate C_18_ 4.6 × 250 mm, 5 μm. 46% MeCN/H_2_O (0.08 M NH_4_H_2_PO_4_, 0.2% Et_3_N)

| Peak | Area | Time | Tailing | Resolution | Area% |
| --- | --- | --- | --- | --- | --- |
| 1 | 4833 | 4.267 | 1.611 | -- | 0.0172 |
| 2 | 13975 | 7.199 | -- | 11.780 | 0.0496 |
| 3 | 28150399 | 9.598 | 1.113 | 7.123 | 99.8926 |
| 4 | 11454 | 13.250 | 0.944 | 9.558 | 0.0406 |

**Compound 8**

Xtimate C_18_ 4.6 × 250 mm, 5 μm. 46% MeCN/H_2_O (0.08 M NH_4_H_2_PO_4_, 0.2% Et_3_N)

| Peak | Area | Time | Tailing | Resolution | Area% |
| --- | --- | --- | --- | --- | --- |
| 1 | 16004 | 5.200 | 1.637 | -- | 0.0839 |
| 2 | 19062552 | 14.571 | 0.937 | 27.769 | 99.9161 |

**Compound 9**

Xtimate C_18_ 4.6 × 250 mm, 5 μm. 46% MeCN/H_2_O (0.08 M NH_4_H_2_PO_4_, 0.2% Et_3_N)

| Peak | Area | Time | Tailing | Resolution | Area% |
| --- | --- | --- | --- | --- | --- |
| 1 | 19880 | 5.345 | 1.190 | -- | 0.1328 |
| 2 | 14947620 | 8.556 | 1.189 | 12.968 | 99.8672 |

**Compound 10**

Xtimate C_18_ 4.6 × 250 mm, 5 μm. 46% MeCN/H_2_O (0.08 M NH_4_H_2_PO_4_, 0.2% Et_3_N)

| Peak | Area | Time | Tailing | Resolution | Area% |
| --- | --- | --- | --- | --- | --- |
| 1 | 2117 | 5.157 | 1.625 | -- | 0.0280 |
| 2 | 1997 | 6.969 | -- | 8.091 | 0.0265 |
| 3 | 2193 | 8.260 | 1.635 | 4.497 | 0.0291 |
| 4 | 7537345 | 13.686 | 1.027 | 14.061 | 99.8640 |
| 5 | 3960 | 19.514 | 0.977 | 11.184 | 0.0525 |

**Compound 11**

Xtimate C_18_ 4.6 × 250 mm, 5 μm. 46% MeCN/H_2_O (0.08 M NH_4_H_2_PO_4_, 0.2% Et_3_N)

| Peak | Area | Time | Tailing | Resolution | Area% |
| --- | --- | --- | --- | --- | --- |
| 1 | 7486789 | 8.260 | 1.271 | -- | 99.6106 |
| 2 | 14736 | 12.922 | 1.348 | 12.737 | 0.1961 |
| 3 | 14531 | 13.911 | 0.883 | 1.919 | 0.1933 |

**Compound 12**

Xtimate C_18_ 4.6 × 250 mm, 5 μm. 46% MeCN/H_2_O (0.08 M NH_4_H_2_PO_4_, 0.2% Et_3_N)

| Peak | Area | Time | Tailing | Resolution | Area% |
| --- | --- | --- | --- | --- | --- |
| 1 | 453532 | 5.045 | 2.369 | -- | 1.8291 |
| 2 | 24341386 | 21.536 | 1.319 | 32.509 | 98.1709 |

**Compound 13**

Xtimate C_18_ 4.6 × 250 mm, 5 μm. 46% MeCN/H_2_O (0.08 M NH_4_H_2_PO_4_, 0.2% Et_3_N)

| Peak | Area | Time | Tailing | Resolution | Area% |
| --- | --- | --- | --- | --- | --- |
| 1 | 233487 | 5.007 | 2.567 | -- | 0.4496 |
| 2 | 54858 | 6.886 | 1.126 | 7.494 | 0.1056 |
| 3 | 290144 | 9.555 | 1.086 | 9.889 | 0.5587 |
| 4 | 51261421 | 13.333 | 2.614 | 7.906 | 98.7066 |
| 5 | 93212 | 20.825 | 1.096 | 11.778 | 0.1795 |

**Compound 14**

Xtimate C_18_ 4.6 × 250 mm, 5 μm. 46% MeCN/H_2_O (0.08 M NH_4_H_2_PO_4_, 0.2% Et_3_N)

| Peak | Area | Time | Tailing | Resolution | Area% |
| --- | --- | --- | --- | --- | --- |
| 1 | 135038 | 5.041 | 1.781 | -- | 0.4509 |
| 2 | 61446 | 7.180 | 1.179 | 8.790 | 0.2052 |
| 3 | 43292 | 9.866 | 1.026 | 9.255 | 0.1446 |
| 4 | 44947 | 12.581 | 1.092 | 7.998 | 0.1501 |
| 5 | 29662585 | 21.465 | 1.400 | 17.018 | 99.0493 |

**Compound 15**

Xtimate C_18_ 4.6 × 250 mm, 5 μm. 46% MeCN/H_2_O (0.08 M NH_4_H_2_PO_4_, 0.2% Et_3_N)

| Peak | Area | Time | Tailing | Resolution | Area% |
| --- | --- | --- | --- | --- | --- |
| 1 | 9000 | 5.002 | 1.687 | -- | 0.0453 |
| 2 | 8294 | 8.597 | 1.017 | 12.653 | 0.0417 |
| 3 | 19843331 | 13.503 | 1.769 | 12.115 | 99.7983 |
| 4 | 22802 | 20.825 | 1.007 | 13.187 | 0.1147 |

**Compound 16**

Xtimate C_18_ 4.6 × 250 mm, 5 μm. 46% MeCN/H_2_O (0.08 M NH_4_H_2_PO_4_, 0.2% Et_3_N)

| Peak | Area | Time | Tailing | Resolution | Area% |
| --- | --- | --- | --- | --- | --- |
| 1 | 73958 | 5.039 | 1.341 | -- | 0.1217 |
| 2 | 29108 | 6.872 | 1.305 | 7.908 | 0.0479 |
| 3 | 55040 | 8.580 | -- | 6.201 | 0.0906 |
| 4 | 60533043 | 9.480 | 3.294 | 1.849 | 99.5982 |
| 5 | 55002 | 14.531 | 1.064 | 9.084 | 0.0905 |
| 6 | 31068 | 15.391 | 0.914 | 1.825 | 0.0511 |

**Compound 17**

Xtimate C_18_ 4.6 × 250 mm, 5 μm. 46% MeCN/H_2_O (0.08 M NH_4_H_2_PO_4_, 0.2% Et_3_N)

| Peak | Area | Time | Tailing | Resolution | Area% |
| --- | --- | --- | --- | --- | --- |
| 1 | 20035 | 4.081 | 1.665 | -- | 0.0695 |
| 2 | 30275 | 4.372 | 2.017 | 1.558 | 0.1050 |
| 3 | 12173 | 5.187 | 1.332 | 4.238 | 0.0422 |
| 4 | 28774277 | 7.399 | 1.936 | 8.447 | 99.7833 |

**Compound 18**

Xtimate C_18_ 4.6 × 250 mm, 5 μm. 46% MeCN/H_2_O (0.08 M NH_4_H_2_PO_4_, 0.2% Et_3_N)

| Peak | Area | Time | Tailing | Resolution | Area% |
| --- | --- | --- | --- | --- | --- |
| 1 | 131414 | 6.520 | 0.981 | -- | 0.8960 |
| 2 | 14535987 | 9.751 | 1.859 | 9.532 | 99.1040 |

**Compound 19**

Xtimate C_18_ 4.6 × 250 mm, 5 μm. 46% MeCN/H_2_O (0.08 M NH_4_H_2_PO_4_, 0.2% Et_3_N)

| Peak | Area | Time | Tailing | Resolution | Area% |
| --- | --- | --- | --- | --- | --- |
| 1 | 103524 | 4.359 | 1.214 | -- | 0.4027 |
| 2 | 25574443 | 7.371 | 1.873 | 12.184 | 99.4760 |
| 3 | 16007 | 10.711 | 1.193 | 8.868 | 0.0623 |
| 4 | 13790 | 14.688 | 1.115 | 8.749 | 0.0536 |
| 5 | 1401 | 24.666 | 0.876 | 21.850 | 0.0054 |

**Compound 20**

Xtimate C_18_ 4.6 × 250 mm, 5 μm. 46% MeCN/H_2_O (0.08 M NH_4_H_2_PO_4_, 0.2% Et_3_N)

| Peak | Area | Time | Tailing | Resolution | Area% |
| --- | --- | --- | --- | --- | --- |
| 1 | 96863 | 6.165 | 0.928 | -- | 0.5357 |
| 2 | 17664263 | 9.294 | 1.861 | 8.768 | 97.6870 |
| 3 | 182044 | 13.781 | 1.377 | 10.992 | 1.0067 |
| 4 | 139340 | 14.635 | 1.077 | 1.909 | 0.7706 |

**Compound 21**

Xtimate C_18_ 4.6 × 250 mm, 5 μm. 46% MeCN/H_2_O (0.08 M NH_4_H_2_PO_4_, 0.2% Et_3_N)

| Peak | Area | Time | Tailing | Resolution | Area% |
| --- | --- | --- | --- | --- | --- |
| 1 | 260515 | 4.985 | 1.682 | -- | 0.8723 |
| 2 | 29589670 | 6.522 | 2.202 | 5.763 | 99.0787 |
| 3 | 14624 | 10.645 | 1.371 | 12.341 | 0.0490 |

**Compound 22**

Xtimate C_18_ 4.6 × 250 mm, 5 μm. 46% MeCN/H_2_O (0.08 M NH_4_H_2_PO_4_, 0.2% Et_3_N)

| Peak | Area | Time | Tailing | Resolution | Area% |
| --- | --- | --- | --- | --- | --- |
| 1 | 154773 | 6.215 | 1.221 | -- | 0.7207 |
| 2 | 21320806 | 9.328 | 1.990 | 9.098 | 99.2793 |

**Compound 23**

Xtimate C_18_ 4.6 × 250 mm, 5 μm. 46% MeCN/H_2_O (0.08 M NH_4_H_2_PO_4_, 0.2% Et_3_N)

| Peak | Area | Time | Tailing | Resolution | Area% |
| --- | --- | --- | --- | --- | --- |
| 1 | 19645766 | 6.558 | 1.924 | -- | 98.3171 |
| 2 | 336281 | 21.643 | 1.069 | 32.346 | 1.6829 |

**Compound 24**

Xtimate C_18_ 4.6 × 250 mm, 5 μm. 46% MeCN/H_2_O (0.08 M NH_4_H_2_PO_4_, 0.2% Et_3_N)

| Peak | Area | Time | Tailing | Resolution | Area% |
| --- | --- | --- | --- | --- | --- |
| 1 | 57668 | 4.865 | 1.037 | -- | 0.3744 |
| 2 | 92622 | 9.821 | 1.110 | 21.024 | 0.6013 |
| 3 | 15252811 | 14.143 | 1.846 | 10.537 | 99.0243 |

**Compound 25**

Xtimate C_18_ 4.6 × 250 mm, 5 μm. 46% MeCN/H_2_O (0.08 M NH_4_H_2_PO_4_, 0.2% Et_3_N)

| Peak | Area | Time | Tailing | Resolution | Area% |
| --- | --- | --- | --- | --- | --- |
| 1 | 71756 | 4.852 | -- | -- | 0.3060 |
| 2 | 100203 | 5.038 | -- | 0.779 | 0.4274 |
| 3 | 23184343 | 10.100 | 2.259 | 14.595 | 98.8830 |
| 4 | 89938 | 14.377 | 1.012 | 10.103 | 0.3836 |

**Compound 26**

Xtimate C_18_ 4.6 × 250 mm, 5 μm. 46% MeCN/H_2_O (0.08 M NH_4_H_2_PO_4_, 0.2% Et_3_N)

| Peak | Area | Time | Tailing | Resolution | Area% |
| --- | --- | --- | --- | --- | --- |
| 1 | 231024 | 8.860 | 0.861 | -- | 1.5158 |
| 2 | 15010220 | 14.119 | 1.839 | 11.544 | 98.4842 |

**Compound 27**

Xtimate C_18_ 4.6 × 250 mm, 5 μm. 46% MeCN/H_2_O (0.08 M NH_4_H_2_PO_4_, 0.2% Et_3_N)

| Peak | Area | Time | Tailing | Resolution | Area% |
| --- | --- | --- | --- | --- | --- |
| 1 | 33648426 | 10.026 | 2.624 | -- | 99.6913 |
| 2 | 50042 | 14.912 | 1.424 | 9.707 | 0.1483 |
| 3 | 54140 | 17.043 | 1.353 | 3.847 | 0.1604 |

**Compound 28**

Xtimate C_18_ 4.6 × 250 mm, 5 μm. 46% MeCN/H_2_O (0.08 M NH_4_H_2_PO_4_, 0.2% Et_3_N)

| Peak | Area | Time | Tailing | Resolution | Area% |
| --- | --- | --- | --- | --- | --- |
| 1 | 260866 | 12.228 | 0.926 | -- | 2.0205 |
| 2 | 12650382 | 20.093 | 1.685 | 12.355 | 97.9795 |

**Compound 29**

Xtimate C_18_ 4.6 × 250 mm, 5 μm. 46% MeCN/H_2_O (0.08 M NH_4_H_2_PO_4_, 0.2% Et_3_N)

| Peak | Area | Time | Tailing | Resolution | Area% |
| --- | --- | --- | --- | --- | --- |
| 1 | 8492578 | 14.697 | 1.748 | -- | 99.5184 |
| 2 | 41096 | 20.570 | 1.054 | 10.521 | 0.4816 |

**Compound 30**

Xtimate C_18_ 4.6 × 250 mm, 5 μm. 46% MeCN/H_2_O (0.08 M NH_4_H_2_PO_4_, 0.2% Et_3_N)

| Peak | Area | Time | Tailing | Resolution | Area% |
| --- | --- | --- | --- | --- | --- |
| 1 | 102921 | 4.605 | 1.377 | -- | 0.4677 |
| 2 | 317836 | 12.205 | 0.916 | 19.408 | 1.4443 |
| 3 | 21585306 | 19.787 | 2.151 | 11.783 | 98.0880 |

**Compound 31**

Xtimate C_18_ 4.6 × 250 mm, 5 μm. 46% MeCN/H_2_O (0.08 M NH_4_H_2_PO_4_, 0.2% Et_3_N)

| Peak | Area | Time | Tailing | Resolution | Area% |
| --- | --- | --- | --- | --- | --- |
| 1 | 30946376 | 14.329 | 2.827 | -- | 99.7770 |
| 2 | 69175 | 20.398 | 1.105 | 9.510 | 0.2230 |

**Compound 32**

Xtimate C_18_ 4.6 × 250 mm, 5 μm. 46% MeCN/H_2_O (0.08 M NH_4_H_2_PO_4_, 0.2% Et_3_N)

| Peak | Area | Time | Tailing | Resolution | Area% |
| --- | --- | --- | --- | --- | --- |
| 1 | 13391 | 4.302 | 1.847 | -- | 0.0768 |
| 2 | 27572 | 5.092 | 1.077 | 2.230 | 0.1581 |
| 3 | 17391936 | 7.309 | 1.742 | 5.465 | 99.7242 |
| 4 | 3676 | 10.565 | 0.967 | 8.848 | 0.0211 |
| 5 | 3454 | 11.866 | 1.237 | 2.999 | 0.0198 |

**Compound 33**

Xtimate C_18_ 4.6 × 250 mm, 5 μm. 46% MeCN/H_2_O (0.08 M NH_4_H_2_PO_4_, 0.2% Et_3_N)

| Peak | Area | Time | Tailing | Resolution | Area% |
| --- | --- | --- | --- | --- | --- |
| 1 | 4572140 | 4.876 | 2.562 | -- | 99.7041 |
| 2 | 8526 | 8.731 | -- | 12.920 | 0.1859 |
| 3 | 5044 | 9.128 | -- | 1.047 | 0.1100 |

**Compound 34**

Xtimate C_18_ 4.6 × 250 mm, 5 μm. 46% MeCN/H_2_O (0.08 M NH_4_H_2_PO_4_, 0.2% Et_3_N)

| Peak | Area | Time | Tailing | Resolution | Area% |
| --- | --- | --- | --- | --- | --- |
| 1 | 186912 | 3.691 | 1.356 | -- | 0.7143 |
| 2 | 114644 | 4.305 | 1.234 | 3.513 | 0.4381 |
| 3 | 25866413 | 7.279 | 1.926 | 11.431 | 98.8476 |

**Compound 35**

Xtimate C_18_ 4.6 × 250 mm, 5 μm. 46% MeCN/H_2_O (0.08 M NH_4_H_2_PO_4_, 0.2% Et_3_N)

| Peak | Area | Time | Tailing | Resolution | Area% |
| --- | --- | --- | --- | --- | --- |
| 1 | 3629644 | 4.909 | 2.724 | -- | 99.7118 |
| 2 | 7172 | 8.394 | 1.073 | 13.216 | 0.1970 |
| 3 | 3320 | 9.978 | 1.688 | 4.338 | 0.0912 |
