# Supplementary material for: Synthesis, Biological Evaluation and Mechanism Studies of Deoxytylophorinine and Its Derivatives as Potential Anticancer Agents
Source: PLoS One. 2012 Jan 19;7(1):e30342. doi: 10.1371/journal.pone.0030342 (PMC3261902; doi:10.1371/journal.pone.0030342)
Supplement: Table S1 — Cytotoxic activities of Compounds 1, 9, 12, 16, 32, 33, 35 in A549 cells for 24 h treatment. These data represent the mean values ± standard deviation of three dependent experiments performed in triplicate. (DOC) [file pone.0030342.s005.doc]

**Table S1. Cytotoxic activities of Compounds 1, 9, 12, 16, 32, 33, 35 *in vitro* for 24 h treatment.**

| Compd | **1** | **9** | **12** | **16** | **32** | **33** | **35** |
| --- | --- | --- | --- | --- | --- | --- | --- |
| IC_50_  (µM) | 18.53±2.14 | 23.83±3.75 | 36.60±1.71 | 31.90±1.53 | 9．05±2.31 | 23.33±2.07 | 50.67±2.28 |

IC_50_ values were all measured for 24 h treatment of Compounds **1, 9, 12, 16, 32, 33, 35** in A549 cells. These data represent the mean values ± standard deviation of three dependent experiments performed in triplicate.
